# Supplementary material for: Quantitative determination of the spatial distribution of components in single cells with CellDetail
Source: Nat Commun. 2024 Nov 26;15:10250. doi: 10.1038/s41467-024-54638-8 (PMC11599593; doi:10.1038/s41467-024-54638-8)
Supplement: Supplementary file 1 — Supplementary Information [file 41467_2024_54638_MOESM1_ESM.pdf]

Supplementary Information: Quantitative determination of the spatial distribution of components in single cells with CellDetail

Tanja Schuster[1*], Amanda Amoah[1,2], Angelika Vollmer[1], Gina Marka[1], Julian Niemann[1], Mehmet Saçma[1], Vadim Sakk[1], Karin Soller[1], Mona Vogel[1], Ani Grigoryan[1], Meinhard Wlaschek[3], Karin Scharffetter-Kochanek[3], Medhanie Mulaw[4] and Hartmut Geiger[1*]

[1*]Institute of Molecular Medicine, Ulm University, Ulm, Germany.
[2*] †Terry Fox Laboratory, BC Cancer Research Centre, Vancouver, Canada.
[3*]Department of Dermatology and Allergic Diseases, Ulm University, Ulm, Germany.
[4*]Unit for Single-Cell Genomics, Ulm University, Ulm, Germany.

*Corresponding author(s). E-mail(s): tanja.schuster@uni-ulm.de; hartmut.geiger@uni-ulm.de;
Contributing authors: aamoah@bccrc.ca; angelika.vollmer@uni-ulm.de; gina.marka@uni-ulm.de; julian.niemann@uni-ulm.de; mehmet.sacma@uni-ulm.de; vadim.sakk@uni-ulm.de; karin.soller@uni-ulm.de; mona.vogel@uni-ulm.de; ani-1.grigoryan@uni-ulm.de; meinhard.wlaschek@uni-ulm.de; karin.scharffetter-kochanek@uniklinik-ulm.de; medhanie.mulaw@uni-ulm.de;

Keywords: spatial distribution, quantification, polarity, protein network, open-source, Septins, hematopoietic stem cells

Contents

A Further application example: Chromosome11 distribution **3**

B Benchmarking of CellDetail **4**

C Robustness of approach **12**

D IF staining - single color controls **15**

E FACS Gating Strategy **16**

F Algorithm **18**
F.1 Quantification of polarity using a dipole moment based method. Basic approach and formulas. 18
F.2 Quantification of polarity using a dipole moment based method. Detailed description of the algorithm and the normalization options. 19

G Validation of code - example scenario **30**

H Manual of CellDetail **32**
H.1 General Description of CellDetail 32
H.2 Fast Start 32
H.3 Detailed Description 36
H.3.1 Tab 1: Import and Export settings 36
H.3.2 Tab 2: Adjust Parameters and Pre-View of cell detection 42
H.3.3 Tab 3: Results 52
H.3.4 Rerun tab 56
H.4 Output 60
H.4.1 Important Parameters for Analysis 60

I Distribution function of absolute value of normalized dipole moment P_n **84**
I.1 Rayleigh formula 84
I.2 Derivation of Rayleigh distribution 84
I.3 Extended Derivation in 3D case 86

J Validation of code - scenarios **88**
J.1 Comparison: Analytical result vs. Algorithm result 88
J.1.1 Description 88
J.1.2 Comparison 88
J.1.3 Two charges 94
J.1.4 Multiple charges 100
J.1.5 Displacement 114

Appendix A Further application example: Chromosome11 distribution

It is known that distributional changes of Chromosome11 occur in murine hematopoietic stem cells upon aging, which are reduced upon treatment with CASIN [18 in manuscript, [2]]: In the young condition the chromosomes are closer to each other, while upon aging they are more distant to each other (see Fig. S1 a). For evaluation manual work was needed for separating the chromosomes and measuring the distance between them.

For testing further application examples of CellDetail, we evaluated the distribution of Chromosome11 from images of previously published data [2] with CellDetail (same settings as for all other evaluations in this manuscript). We found that CellDetail detected the age-related change of Chromosome11 distribution as well as its rescue upon CASIN treatment by P_n (S1 b). P_n is thus not only a value for assessing polarity changes of cell components, but as well more general distributional changes of cell components. Thus, the applicability range of CellDetail is not limited to cell polarity research questions alone, but is expanded to research questions about more general distributional changes.

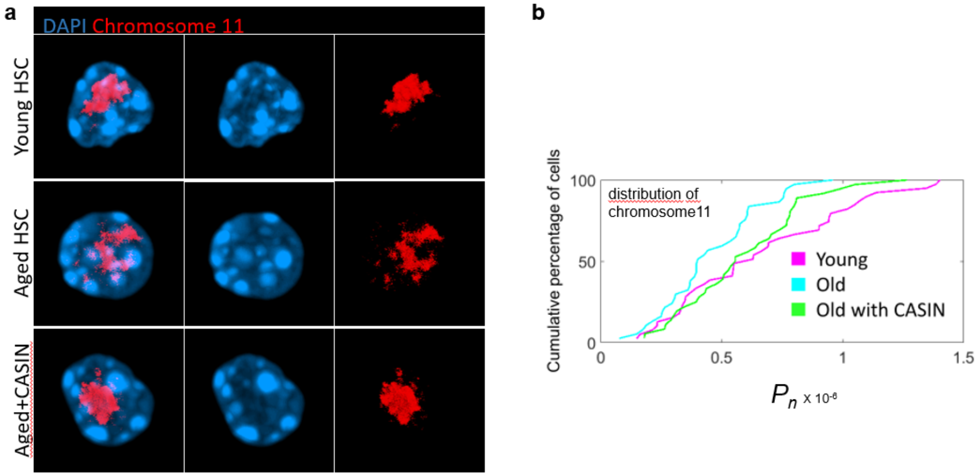

Fig. S1 a) Confocal IF example images of Chromosome11 staining in young and old murine hematopoietic stem cells
b) Cumulative percentage of cells over P_n of Chromosome11 (re-evaluation of published data by CellDetail). n_{young} = 39, n_{old} = 37, $n_{old,CASIN}$ = 36, median: $P_{n,young}$=0.63e-6, $P_{n,old}$=0.40.e-6, $P_{n,old,CASIN}$=0.56e-6, $p_{young\ vs.\ old}$ = 0.02; effect size: $D_{young\ vs.\ old}$=0.35; $p_{young\ vs.\ old\ with\ CASIN}$ = 0.56; effect size: $D_{young\ vs.\ old\ with\ CASIN}$=0.46; $p_{old\ vs.\ old\ with\ CASIN}$ = 0.03; effect size: $D_{old\ vs.\ old\ with\ CASIN}$=0.35;confocal (Source data are provided as a Source Data file.)

Appendix B Benchmarking of CellDetail

For a benchmarking analysis of CellDetail against other existing tools, the following methods/tools were compared: Dipole moment method (CellDetail), Barycenter/Center of mass method (self-implemented), Barycenter/Center of mass method with normalization (self-implemented), intensity profile fit method (POME [1], resulting in standard deviation values, amplitude values and baseline values used on a self-trained generalized linear model), Ratio method (QuantifyPolarity [3]), Fourier analysis method (QuantifyPolarity), PCA method (QuantifyPolarity) and 2D Artificial Neural Network method (self-implemented, training data 67% and validation data 33% of 210 completely independently and with datagenerator modified 2D layers (translational and rotational modification for generalization), optimized for a 64-node-7-convolutional/maxpool-layers-0-dense-layers set-up according to lowest validation loss (0.23) with validation accuracy of 90.0% and resulting test accuracy of 97.4% over all polar and apolar Cdc42 2D test layers.).

As QuantifyPolarity and POME are tools that are restricted to 2D due to the fact that parts of their methods are limited to 2D analyses, the comparison between methods had to be based on single 2D layer images. The 3D image stacks of HSCs stained for Cdc42 were split up into single 2D layers to provide a dataset of independent 37 polar and 42 apolar 2D layers of HSCs stained for Cdc42 (see Fig. S2A). Polar and apolar image information were provided as a 2D slice to the methods to evaluate how well the methods could separate polar and apolar image information. Cell masks necessary for most of the methods/tools were provided by CellDetail. These cell masks were used for the other methods in order to give every tool/method the same cell voxel vs. background information as CellDetail.

During the process of applying the tools POME and QuantifyPolarity on the 2D data set, limitations of these tools became obvious: POME with the intensity fit method requires programming skills in R. QuantifyPolarity requires cell masks with a hole, as this tool was programmed for membrane proteins. To include also these methods in the benchmarking analyses, cell masks were adjusted by including a hole in the middle of the cell. The resulting 2D layers with the adapted cell masks were analyzed with CellDetail (again) and QuantifyPolarity (Ratio method, Fourier method, PCA method). This required to perform 2 types of benchmark comparisons: Fig. S2 b-e with common cell masks and Fig. S2 b'-h with adapted cell masks. The benchmarking analysis of the neural network, which does not make use of cell vs. background masks, is visible in Fig. S2 i,j. POME and QuantifyPolarity each could not analyze all cells, but gave out an error due to bad fit for POME (1 apolar cell) and an unknown reason for QuantifyPolarity (1 polar cell).

Fig. S2 b-h show the histograms of the polarity parameter output of b) Dipole moment method, c) Center of mass method, d) Center of mass method with normalization, e) Intensity profile fit (POME), b') Dipole moment method

with adapted cell masks (CellDetail), f) Ratio method (QuantifyPolarity), g) Fourier method (QuantifyPolarity), h) PCA method (QuantifyPolarity) for the established data set consisting of 37 polar and 42 apolar Cdc42 2D cell layers. The polar data set is colored in blue and the apolar data set in red in the count histograms. The dipole moment method of CellDetail (b and b') as well as the barycenter method (with and without normalization) separate the polar and apolar Cdc42 2D layers best. For the neural network a prediction confusion plot is shown (Fig. S2 j), which shows the percentages of correctly and incorrectly assigned 2D layers. One cell was left undefined due to its probability falling into the 95% confidence interval restriction received from the normalized inverse cumulative sum of frequency of probabilitiy outcome for the 210 layers (see Fig. S2 i). The assignment is non-random (Fisher's exact test on confusion matrix, $p = 6.2e\text{-}20$).

In summary, the dipole moment method in CellDetail together with the barycenter method (with/without normalization) separated 2D layers of HSCs visually assigned to be polar and apolar in Cdc42 with an accuracy of 98.7% (Table S1). The dipole moment method in CellDetail though offers additional benefits compared to the barycenter method (for example i) it can distinguish between different types of polarity, ii) it provides a higher level of sensitivity in terms of protein cluster center position, and iii) in CellDetail the polarity vector is not being forced through the center of the cell to then provide more detailed information on the actual relative positions of charges), which are parameters that could not be tested in a comparative fashion in the benchmarking analyses.

A table with in-depth qualitative comparisons of the different polarity quantification methods used in our benchmarking with examples of tools that implemented them, is shown in Figs. S3-S5.

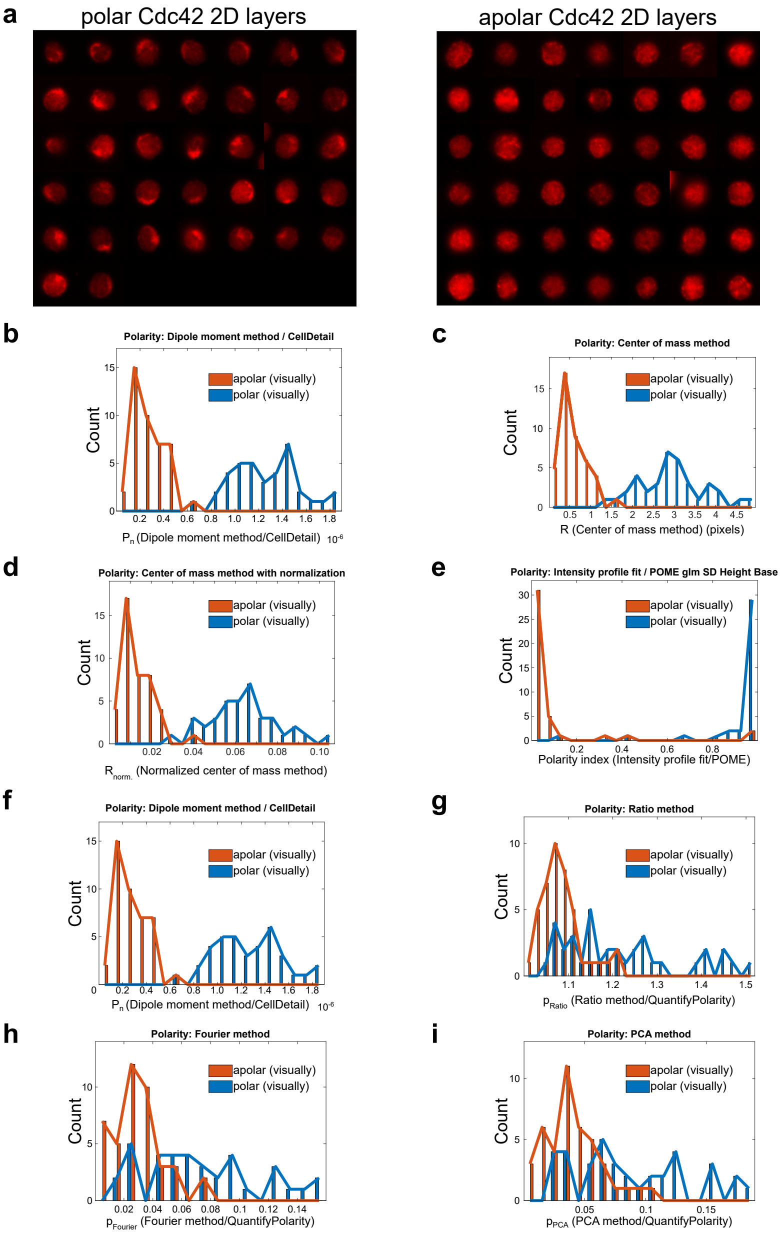
a
polar Cdc42 2D layers
apolar Cdc42 2D layers
b
Polarity: Dipole moment method / CellDetail
apolar (visually)
polar (visually)
Count
P_n (Dipole moment method/CellDetail) 10^-6
c
Polarity: Center of mass method
R (Center of mass method) (pixels)
d
Polarity: Center of mass method with normalization
R_norm. (Normalized center of mass method)
e
Polarity: Intensity profile fit / POME glm SD Height Base
Polarity index (Intensity profile fit/POME)
f
Polarity: Dipole moment method / CellDetail
P_n (Dipole moment method/CellDetail) 10^-6
g
Polarity: Ratio method
p_Ratio (Ratio method/QuantifyPolarity)
h
Polarity: Fourier method
p_Fourier (Fourier method/QuantifyPolarity)
i
Polarity: PCA method
p_PCA (PCA method/QuantifyPolarity)

Fig. S2

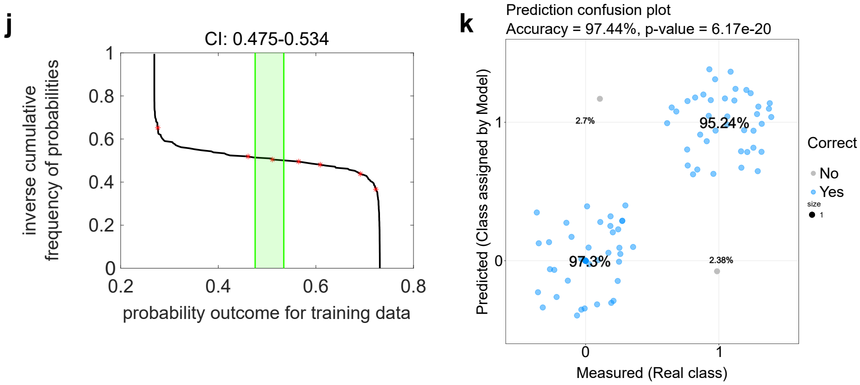

Fig. S2 Benchmarking analysis. Source data are provided as a Source Data file. a) data set for validation. 2D layers of Cdc42 distribution from individual cells, 37 polar and 42 apolar cells. b)-k) Benchmarking analyses. b)-i) Count histograms are shown for the polarity outcome of different methods, with polar Cdc42 2D layer inputs shown in blue and apolar Cdc42 2D layer inputs shown in red. The x-axes differ due to different methods used for analysis of polarity. b) Dipole moment method (tool: CellDetail). c) Center of mass method. d) Center of mass method with normalization. e) Intensity profile fit (tool: POME). f) Dipole moment method (tool: CellDetail) with adapted mask to render data compatible for the subsequent benchmarking against the tool QuantifyPolarity. The tool QuantifyPolarity (that can use a Ratio method, a Fourier method or a PCA method) requires a cell mask that excludes the cytoplasm. It was necessary to adapt the input data by inserting a background pixel in the middle of the cell masks used and let it run through CellDetail / dipole moment method again to ensure consistency for comparing results with QuantifyPolarity. g) Ratio method (tool: QuantifyPolarity). h) Fourier method (tool: QuantifyPolarity). i) PCA method (tool: QuantifyPolarity). j) Neural network: Inverse cumulative frequency of probabilities over probability outcome for training data plot for determining 95% confidence interval (limits to assignment probabilities). Red markers show misclassified Cdc42 2D cell layers of the training data. Cells falling within the confidence interval (green box) are undefined. k) Prediction confusion plot with percentage of true polar, false polar, false apolar and true apolar assigned polar and apolar Cdc42 2D cell layers.

Table S1 Benchmarking analysis. Source data are provided as a Source Data file. Percentage of accuracy based on overlap of polarity values for polar and apolar Cdc42 2D cell layers.

Method	accuracy (%)
dipole moment method	98.7%
barycenter method	98.7%
barycenter method with normalization	98.7%
intensity profile fit method	94.9%
ratio method	73.4%
Fourier method	69.6%
PCA method	69.6%
2D neural network	97.4%

Tool or, if not existent, example paper	CellDetail	Amanda Amoah et al. [1]	PolarityJAM (bioRxiv), POME (side-information) ImageJ macro
Method	dipole moment method	visual categorization	barycenter / center of mass
Short Description	subtracting average cell intensity value from single cell intensity values<br>➡ positive and negative values<br>➡ barycenters of positive and negative values<br>➡ distance between positive/negative barycenter multiplied by summed positive values: "dipole moment"<br>➡ normalization P_n	defining cell protein state as polar or apolar based on visual categorization	calculation of barycenter of intensity values of cell
Advantages of methods	applicable on different cell types, independent of cell component distribution beforehand known (like e.g. cortical protein or nuclear protein) and related to cell morphology (always in uropod, or next to cilia base) or not	applicable on different cell types, independent of cell component distribution beforehand known and related to cell morphology or not	applicable on different cell types, independent of cell component distribution beforehand known and related to cell morphology or not
Additional advantages of existing tools	cell detection included, further spatial distribution parameters	no	cell detection and segmentation included, further parameters like tissue-related parameters, visualization
Limitations of method		requires trained researchers, binary output for polarity, how to treat difficult cells (leave out, force decision)	indistinguishable scenarios of polarity, less sensitive than dipole moment method
Limitations of existing tools	implemented for single cells; dependent on component of interest additional staining or cell mask needed		dependent on tool, e.g. only in 2D (POME, PolarityJAM), single cells and background voxels set to 0 needed and additional calculation steps (ImageJ)
Applications	HSCs (Isotropic cell type, with no clear region of interest defineable by morphology or protein behavior), fibroblast strain FF95	correctly oriented organelles and proteins in HSCs, fibroblasts etc. [Wang2003, Chacko2005, Amoah2022], organoid shape of spheroids [Okuyama2016]	Notchl polarity in endothelial cell culture
Methods useable for our application	yes	yes	yes
Implemented tools useable for our application	yes		(only in 2D or extra effort needed)
Polarity parameter	P_n	% polar, % apolar	distance Middle-Barycenter, can be normalized by radius
Further polarity related parameters	R_n contribution of spatial separation and q_n contribution of charge to polarity; direction of polarity related to cell content ($R_{neg}R_{pos}$) and related to middle of cell (MR_{pos}), constriction of polarity gradient within cell subregion or across cell	no	direction of polarity related to middle of cell
applicable to 2D	yes	yes	yes
applicable to 3D without projection	yes	yes	yes
3D polar. sensitive	yes	yes	yes

Fig. S3 Qualitative comparison of different methods for polarity quantification. Dipole moment method. Visual categorization. Barycenter method. Intensity ratio method. Intensity profile fit method. Angle method. Fourier method. PCA method. Neural Network.

Tool or, if not existent, example paper	SEGGA (cortical proteins), QuantifyPolarity (cortical proteins), PolarityJAM (bioRxiv)	POME (cortical proteins), Azimuthal average (Fiji, dependent on usage: ratio or intensity profile method)	PolarityJAM (bioRxiv)
Method	intensity ratio	intensity profile	angle
Short Description	definition of regions of interest and comparison of corresponding intensities	from the intensity profile deduction of localization parameters (e.g. after fitting of Gaussian)	angle of ciliar bundles or Golgi-nucleus angle across whole tissue
Advantages of methods	manually: applicable on different cell types, independent of cell component distribution beforehand known and related to cell morphology or not<br>if not manually:<br>predictable protein behavior for segmentation needed (not useable for every case)	can characterize existing poles more (for width, intensity, etc.)	applicable on different cell types, independent of cell component distribution beforehand known and related to cell morphology or not
Additional advantages of existing tools	planar polarity on single cell and tissue level, segmentation included dependent on tool	segmentation included dependent on tool	several possibilities for cell/nucleus parameters segmentation included
Limitations of method	no directionality of polarity,<br>just relative intensity magnitude parameter not possible to automatize for every case	need to know relative position of ROI,<br>usually method ignores radial sensitivity	needs relative point of reference (like directionality in tissue) which is not always given; method not in general applicable
Limitations of existing tools	implemented for membrane proteins, needs membrane staining (SEGGA, QuantifyPolarity); segmentation into two halves made dependent on cell shape but not on distribution, which is not ideal (PolarityJAM)	implemented for membrane proteins, needs membrane staining (POME);<br>Azimuthal average: fit of circle can have deviations from cell shape	only 2D (PolarityJAM)
Applications	intensity difference in diverse cell compartments [Krieghoff2006], intensity switch from front to back or transverse to vertical edges [Wang2002, Farrell2017], polarity establishment intensity switch [Glanc2018]	membrane proteins (POME) with crescent polarity, secretion polarity (Azimuthal angle, fit of circle), determination of related distance of positions [Bryant2014]	Golgi-nucleus in endothelial cell culture (PolarityJam), microtubule directionality [Toya2016], ciliary bundle directionality [Qian2007]
Methods useable for our application	no (would need to optimize for cutting cell into halves finding right position of cutting)	no (would need projection, which limits 3D sensitivity of polarity)	no
Implemented tools useable for our application	no	no (2D, not 3D polarity sensitive)	no (implemented for tissue polarity, and comparison of directionality of relative polarity vectors of e.g. Golgi to nucleus within different cells in tissues)
Polarity parameter	(log2 ratio of intensities) ratio of intensities	position of peak, parameters of fitted shape (mostly Gaussian)	angle distribution or combined information in vector for mean angle distribution of tissue (magnitude and angle)
Further polarity related parameters	no	direction of polarity related to middle,<br>width of pole/s,<br>amount contributing to pole/s	direction of polarity
applicable to 2D	yes	yes	yes
applicable to 3D without projection	yes<br>(dependent on ROI selection)	POME not, Azimuthal average yes	would be possible, but if viewing angle well chosen not needed
3D polar. sensitive	(dependent on ROI selection)	(dependent on intensity plot line)	no

Fig. S4 Qualitative comparison of different methods for polarity quantification. Dipole moment method. Visual categorization. Barycenter method. Intensity ratio method. Intensity profile fit method. Angle method. Fourier method. PCA method. Neural Network.

Tool or, if not existent, example paper	QuantifyPolarity	QuantifyPolarity	Neural Network
Method	Fourier	PCA	Neural Network
Short Description	0-360° intensity profile gets Fourier decomposed Fourier coefficients lead to angle and magnitude of polarity vector	recalculation of cell shape for excluding elongation effects (compression) ➡ recalculation of cell pixel positions according to intensity ➡ PCA with resulting vector dependent on eigenvalues and eigenvectors	defining cell protein state as polar or apolar based on visual categorization, training of a neural network with images
Advantages of methods		shape insensitive	applicable on different cell types(if trained on them), detection of hidden features (unsupervised)
Additional advantages of existing tools	further cell parameters included, applicable on cell layers and single cells, polarity quantification methods of intensity ratio, Fourier and PCA all included in tool QuantifyPolarity		
Limitations of method	radial insensitive; method not in general applicable	intracellular/ radial insensitive; bipolarity concept of planar polarity (opposite junctions vs. other junctions: relative intensity difference); method not in general applicable	more effort for generalization over different cell types and biomolecules of interest
Limitations of existing tools	intracellular/radial insensitive; sensitive to cell geometry; bipolarity concept of planar polarity NOT generally applicable (opposite junctions vs. other junctions: relative intensity difference), needs cell object masks with inner holes	insensitive to radial distance from middle (for membrane component rather than intracellular component spatial distribution); needs cell object masks with inner holes	
Applications	planar polarity [Aigouy2010]	planar polarity [Tan2021]	
Methods useable for our application	no (indifference of radial distance to middle of cell)	no (indifference of radial distance to middle of cell)	yes
Implemented tools useable for our application	no	no	
Polarity parameter	magnitude and angle (vector)	magnitude and angle (vector)	% polar, % apolar
Further polarity related parameters	direction of polarity	direction of polarity	no
applicable to 2D	yes	yes	yes
applicable to 3D without projection	no	no	yes
3D polar. sensitive	no	no	yes

Fig. S5 Qualitative comparison of different methods for polarity quantification. Dipole moment method. Visual categorization. Barycenter method. Intensity ratio method. Intensity profile fit method. Angle method. Fourier method. PCA method. Neural Network.

Appendix C Robustness of approach

For the validation of the robustness of CellDetail, the following properties were considered important:

a) The influence of variation of brightness on the result.
b) The influence of Signal-to-noise ratio on the result.
c) The influence of oversaturation of pixels on the result.
d) The influence of shape at fixed area size on the result.

For the validation five cell image stacks of Cdc42 staining were chosen whose polarity P_n values for Cdc42 were spread across the P_n polarity index spectrum (Fig.S6 a,b).
For the evaluation of the influence of brightness variation on the P_n polarity outcome, the intensity of image stacks of the individual cells was altered by multiplying the pixel intensity values by a factor of 1 to 10. After the CellDetail run-through on these intensity manipulated images, we observed only minor differences in the P_n over their intensity multiplication factor (percentage differences are below 3%) (see Fig. S6 b). The algorithm is therefore robust with respect to intensity multiplication. When pixel intensity values exceeded the pixel saving size, the intensity manipulated images were not taken into account for the analysis of brightness variation as the exceeding of pixel saving size means oversaturation (e.g the data for cell 4 ends at an intensity multiplication factor of 4). Such images though were instead used for the latter case of oversaturation, by modifying the exceeded pixel values back to the maximal possible pixel intensity value (in this case set to $2^{14} - 1$).This means that P_n values are comparable in the following different scenarios:

- measurement of the biomolecule of interest with a different exposure time
- measurement of the biomolecule of interest with different staining concentrations of primary / secondary antibody
- measurement of the biomolecule of interest with another secondary antibody fluorophore staining
- biomolecule A polarity against biomolecule B polarity

For the evaluation of the robustness of the signal-to-noise ratio (SNR), white Gaussian noise signal was added to the images. 0.1 means a signal-to-noise ratio of 1 signal- to 10 noise- intensity. This was done by using the Matlab command *awgn*. In such a scenario, it is anticipated that with lower value of SNR, the value of the deviation from the original P_n increases. Fig. S6 c shows indeed an increase in the deviation from the original value of P_n at lower SNR values. Cell 1 presents with the largest deviation between original value without added noise to added noise (35%), while the other cells show a deviation of around 7-10% (cell 2 - cell 5). The higher impact of the added noise to an almost complete apolar distribution is likely due to the fact that noise will affect several smaller clusters more than a single big (polar) cluster. The lowered SNR though does not change the order of the cells in terms of

their polarity: The polarity increases from cell 1 to cell 5 at every level of SNR. CellDetail is therefore robust with respect to variations in the SNR.

For the evaluation of the robustness with respect to an oversaturation of pixels, the image stacks of individual cells were multiplied with intensity multiplication factors that made pixel intensities exceed the saving bit depth of 14 (maximal possible value thus $2^{14} - 1$). Values that then exceeded the maximal possible value were re-set to the maximal possible value, and their percentage relative to the volume was assessed. Data in Fig. S6 d (% oversaturation over P_n) shows that the influence of oversaturation on P_n is larger than that of brightness variation or SNR. Still, even at the level of oversaturation of 40% of all pixels within the image, the cells remain in the same order on the polarity axis compared to no oversaturation. Oversaturation of pixels is in general an avoidable artifact when measuring samples. While usually oversaturation has a strong impact on the ability to score polarity by visual examination, CellDetail is also in this respect quite robust, in as cells remain in their relative order of polarity - even at very high levels of oversaturation.

For testing the robustness of P_n with respect to cell shape, the maximal diameter of a line, circle, hexagon and square was analytically calculated in dependence of their area and the dipole moment algorithm was applied on the line, circle, hexagon and square for a charge sitting at the edge of the maximal diameter of the geometrical shape resulting in the outcome of the polarity value P_n. The difference in percentage of P_n (Fig. S6 e) between different shapes decrease with an increase of the size of the area. The size of the cell area of a single layer image of an HSC is in the range from 4000 to 8000 pixels (shown as yellow colored area), that of an FF95 cell is in the range of 30,000 pixels up to 300,000 pixels (not shown here). Even for small cells like HSCs the percentage difference is below 1% for the biorealistic difference of non-line shapes. The line comparisons were included in this analysis to find the maximal possible deviation in percentages (line-circle comparison as maximal diameters differ the most at fixed area). CellDetail is thus also robust with respect to variations in cell shape.

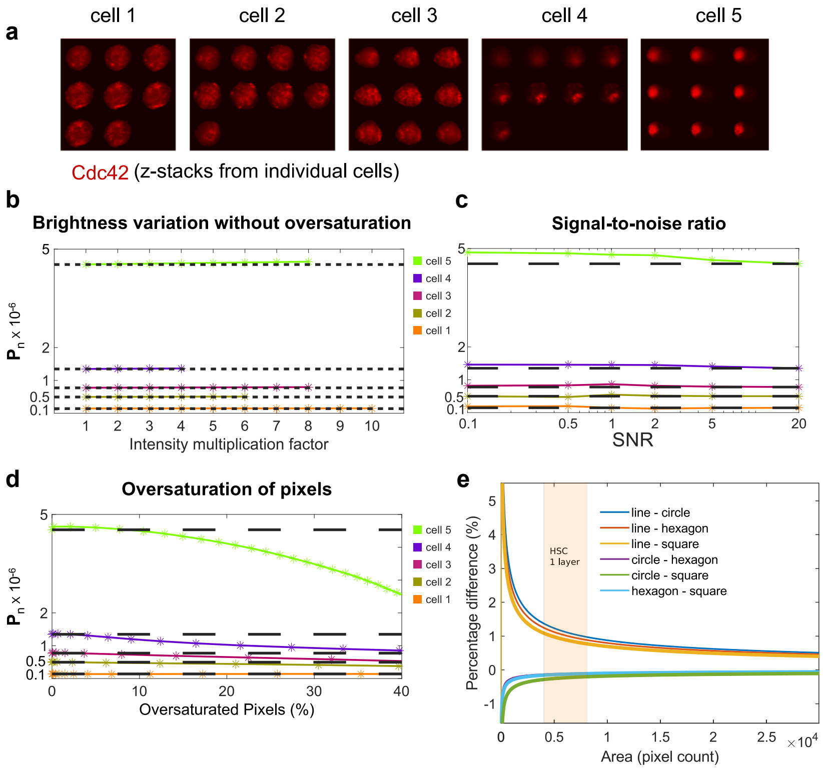

Fig. S6 Analyses of robustness of CellDetail. Source data are provided as a Source Data file. a)-d) on Cdc42 3D IF stacks.
a) Image stacks of five cells stained for Cdc42. P_n values of these cells cover the range of polarity values obtained for HSCs.
b) Brightness variation test outcome for evaluating the influence of intensity variations on the polarity outcome. The polarity parameter P_n is shown against different intensity multiplication factors.
c) Signal-to-noise (SNR) ratio test outcome for evaluating the influence of noise on the polarity outcome. P_n is shown against the SNR.
d) Oversaturation test outcome. P_n is shown against the percentage of oversaturated pixels.
e) Influence of shape test outcome. The percentage difference of P_n for two different shapes of same area size with a charge set at the maximal distance on the outer edge of the maximal diameter are shown against area. The area in pixel count that one layer of hematopoietic stem cells can have is indicated as yellowish box.

Appendix D IF staining - single color controls

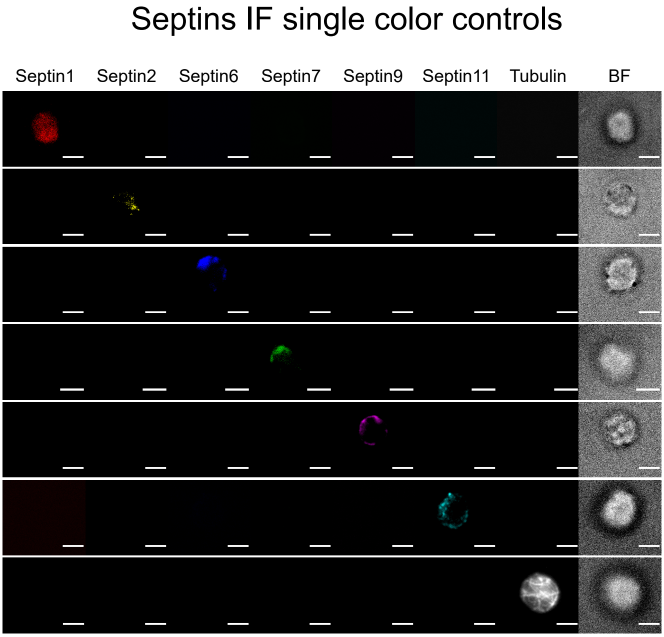

Fig. S7 IF stainings on individual HSCs. Single color controls for Septin1, Septin2, Septin6, Septin7, Septin9, Septin11 and Tubulin staining. Scale bar 5µm.
For the 7 color IF measurements, the filters Semrock FF01-595/31, Semrock FF01-676/29, Zeiss PBP 425/30+514/31+592/25+681/45+785/38, Zeiss QBP 425/30 + 524/51 + 634/38 + 785/38 and Zeiss TBP 467/24+555/25+687/145 were used with the beam splitters TBS 450+538+610, QBS 405+493+611+762, PBS 405+493+575+654+761, BS573, BS652 and with Zeiss Colibri 7 LEDs (385 nm LED, 430 nm LED, 475 nm LED, 555 nm LED, 590 nm LED, 630 nm LED, 735 nm LED).

Appendix E FACS Gating Strategy

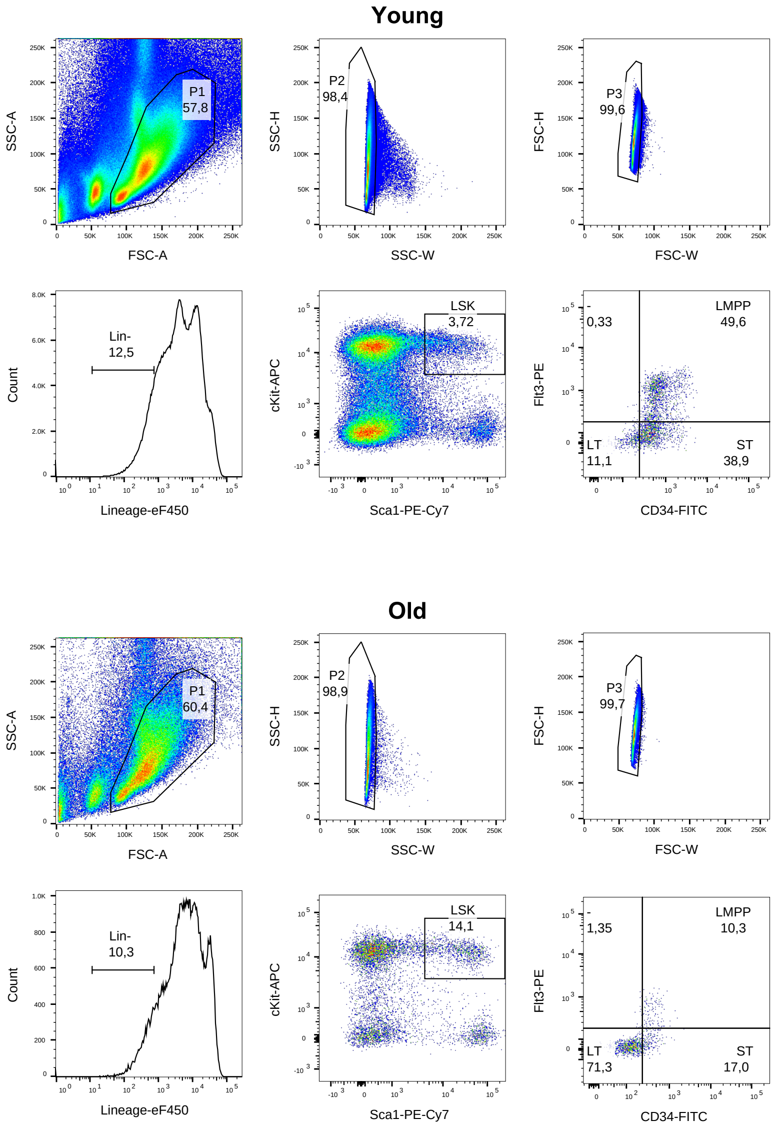

Fig. S8 FACS Gating Strategy for HSCs (longterm HSCs, LT-HSCs or here LT). Representative FACS dot plots of young and old HSCs (same gating strategy). SSC-A vs FSC-A: viable cells gating. SSC-H vs. SSC-W: removal of duplets/triplets , FSC-H vs. FSC-W: removal of duplets/triplets; Lineage-eF450: lineage depleted cells taken, LT-HSCs: $Lin^{-}c\text{-}Kit^{+}Sca\text{-}1^{+}Flt3^{-}CD34^{-}$

Appendix F Algorithm

F.1 Quantification of polarity using a dipole moment based method. Basic approach and formulas.

We implemented a dipole moment based method to quantify 2D and 3D polarity in single cells. To this end, voxels are separated first into background voxels and cell voxels (cell detection). Next, the average intensity of background voxels is calculated and subtracted from the cell voxel intensities to obtain background corrected voxels for each cell (cell voxels or from now on simply voxels). In a next step, the intensity of the voxels can be normalized to average cell intensity per voxel being 1. This is a normalization option within CellDetail.

Afterwards, the average intensity of voxels is calculated, with n_{voxel} being the number of voxels and $i_{k,cell}$ being the voxel intensity of voxel k,

$$i_{av} = \frac{\sum_{k=1}^{n} i_{k,cell}}{n_{voxel}}. \tag{F1}$$

Afterwards, the charges q are calculated by subtracting i_{av} from each voxel intensity value:

$$q_k = i_{k,cell} - i_{av}. \tag{F2}$$

This results in positive charges $q_{pos,k}$ and negative charges $q_{neg,k}$ with $n_{pos,voxel}$ as number of positive charges and $n_{neg,voxel}$ as number of negative charges. In the special case of q_k being 0, the value does not contribute to later charge center calculation and is thus ignored. Charge center positions are required to calculate the dipole moment. To this end, the positive charge center $\mathbf{R}_+$ is calculated as

$$\mathbf{R}_+ = \frac{\sum_{k=1}^{n_{pos,voxel}} q_{pos,k} \cdot \begin{pmatrix} x_k \\ y_k \\ z_k \end{pmatrix}}{\sum_{j=1}^{n_{pos,voxel}} q_{pos,j}} \tag{F3}$$

with (x_k, y_k, z_k) being the position of cell voxel k with charge $q_{pos,k}$, and the negative charge center $\mathbf{R}_-$ is calculated as

$$\mathbf{R}_- = \frac{\sum_{k=1}^{n_{neg,voxel}} q_{neg,k} \cdot \begin{pmatrix} x_k \\ y_k \\ z_k \end{pmatrix}}{\sum_{j=1}^{n_{neg,voxel}} q_{neg,j}} \tag{F4}$$

with (x_k, y_k, z_k) being the position of cell voxel k with charge $q_{neg,k}$. The distance vector $\mathbf{d}$ between charge center positions is calculated by

$$\mathbf{d} = \mathbf{R}_+ - \mathbf{R}_-. \tag{F5}$$

The resulting dipole moment describing the polarity of a spatial distribution is calculated as

$$\mathbf{P} = Q_+ \cdot \mathbf{d} \tag{F6}$$

with $Q_+ = \sum_{k=1}^{n_{pos,voxel}} q_{pos,k}$. For the calculation of the absolute value of normalized dipole moment P_n, the distance between charge centers (d,length of $\mathbf{d}$) is needed. $\mathbf{P}$ is normalized in a final step leading to

$$P_n = \frac{Q_+}{normalization} \cdot \frac{d}{normalization} = q_n \cdot R_n \tag{F7}$$

For all data analysis in this manuscript, the following normalizations were used: The average intensity per voxel was set to 1 with charge normalization option q_{n1} as well as the distance normalization option R_{n1} (see next section for detail).

F.2 Quantification of polarity using a dipole moment based method. Detailed description of the algorithm and the normalization options.

CellDetail uses immunofluorescence images of single cells for the assessment of distribution of stained components. Single layer or z-stacked tiff images of single cells are used as input. Cell detection is included in CellDetail, another possibility is the usage of pre-made cell-background masks. Cell detection is based on Otsu's method for automatized thresholding. It is split into thresholding for cell layers and after recognizing cell layers thresholding for cell voxels against background voxels. After cell detection the average background intensity is subtracted. The average intensity value of cell voxels is calculated and subtracted from cell voxel intensities leading to positive and negative voxel values as can be seen in Figure 1e (manuscript). The positive and negative voxel values correspond to regions with higher and lower protein content and are considered as positive and negative charges. The new method for quantitative analysis of polarity is derived from the quantity dipole moment $\mathbf{P}$. The distance between charge-weighted positive and negative charge center multiplied with absolute value of charge gives the dipole moment whose magnitude is considered as a measure for a polar distribution of the stained component. As cells vary in size and component (e.g. protein content), the possible maximal distance of charge centers is higher in bigger cells and the possible maximal charge is dependent on protein expression, which shows age-related changes, and cell size. Thus, dependent on cell size and protein content, cells can have an advantage in achieving higher polarity values. Normalizations of the distance (see Fig. S9), charge (see Fig. S10) and protein amount are used to make polarity comparable, as otherwise polar but weak intensity cells are overwhelmed by apolar high intensity cells and small cells have a general disadvantage in possible distance between charge centers.

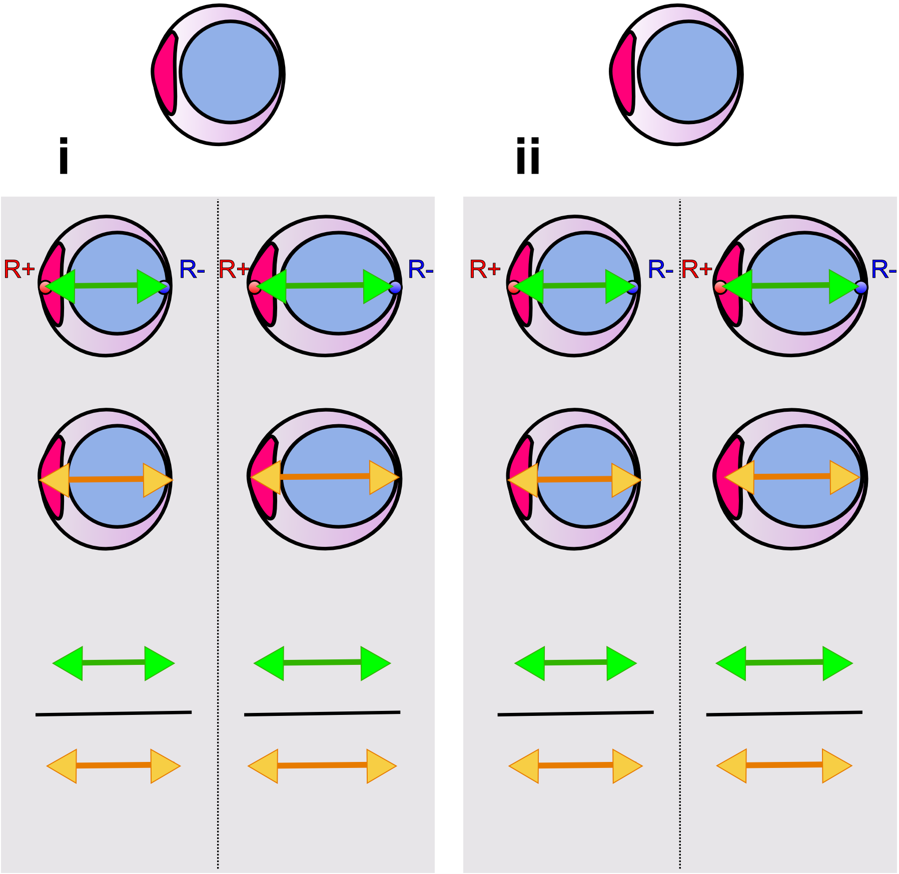

Fig. S9 Diameter normalization options. (i) Maximal diameter option. Distance d between positive charge center (red dot) and negative charge center (blue dot) shown by green arrow divided by maximal diameter shown by orange arrow for both a true isotropic and elongated cell. $R_{n1} = \frac{d}{maximal\ diameter}$ (ii) Averaged diameter option. Distance d between positive charge center (red dot) and negative charge center (blue dot) shown by green arrow divided by averaged diameter shown by orange arrow for both true isotropic and elongated cell. $R_{n2} = \frac{d}{average\ diameter}$

An overview over all normalization possibilities is provided in Tab. S2. A guideline for choosing normalization options is shown in Figures S12, S13 and S14.

CellDetail offers two options for distance normalization, five options for charge normalization and two options for protein content normalization. Distance normalization can be based on the maximal or the average cell diameter (Fig.S9 (i) maximal, (ii) average).

As a general view, the charge normalization can be done bit-based (i q_{n1}, ii q_{n2}), cell-based (iii q_{n3}, iv q_{n4}) and relative to initial intensity values (vi q_{n6})

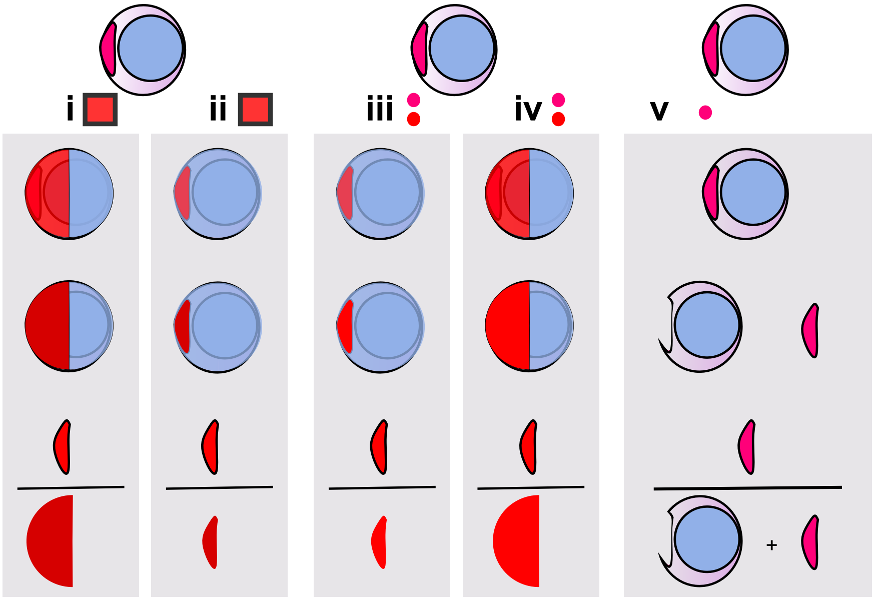

Fig. S10 Charge normalization options with q_n as normalized charge, q_{pos} as positive charge of individual voxels, n_{voxel} as the number of voxels of cell volume, $i_{background}$ as average intensity of background voxels, $i_{average}$ as average intensity of cell voxels, $n_{pos,voxel}$ as number of positive charge voxels. (i) bit-based. Half of the cell volume is taken as maximal possible volume of positive charge, the maximal possible charge is defined by bits used to store single voxels in $(2^{bit} - 1)$. $q_{n1} = \frac{\sum q_{pos}}{0.5 n_{voxel} \cdot (2^{bit} - 1 - i_{background} - i_{average})}$ (ii) bit-based. The volume of positive charge is taken as maximal volume of positive charge, the maximal possible charge is defined by bits used to store single voxels in $(2^{bit} - 1)$. $q_{n2} = \frac{\sum q_{pos}}{n_{pos,voxel} \cdot (2^{bit} - 1 - i_{background} - i_{average})}$ (iii) individual maximum charge based. The volume of positive charge is taken as maximal volume of positive charge, the maximal charge of the individual cell $q_{pos,max}$ is taken for defining the maximal possible value of charge a cell can have. $q_{n3} = \frac{\sum q_{pos}}{n_{pos,voxel} \cdot q_{pos,max}}$ (iv) individual maximum charge based. Half of the cell volume is taken as maximal possible volume of positive charge, the maximal charge of the individual cell $q_{pos,max}$ is taken for defining the maximal possible value of charge a cell can have. $q_{n4} = \frac{\sum q_{pos}}{0.5 n_{voxel} \cdot q_{pos,max}}$ (v) recalculation to original intensity. The proportion of intensity belonging to positive charge compared to total intensity is calculated. $q_{n6} = \frac{\sum q_{pos} + (i_{average} \cdot n_{pos,voxel})}{n_{voxel} \cdot i_{average}}$

(see Fig. S10). The maximal possible charge can either be defined by cell size (half cell size, for options i q_{n1}, iv q_{n4}) or by component distribution with just the positive charge voxels having maximal charge value (ii q_{n2}, iii q_{n3}). The latter case gives cells an advantage in which components are more efficiently clustered on a small volume than in other cells. For the case of vi q_{n6} the percentage of intensity that the positive charge voxels have from the initial total intensity for a component of the cell is calculated. The options using bit depth for setting a margin to maximal charge provide a more global standard than a cell-based option, for cells with low protein content can't achieve high

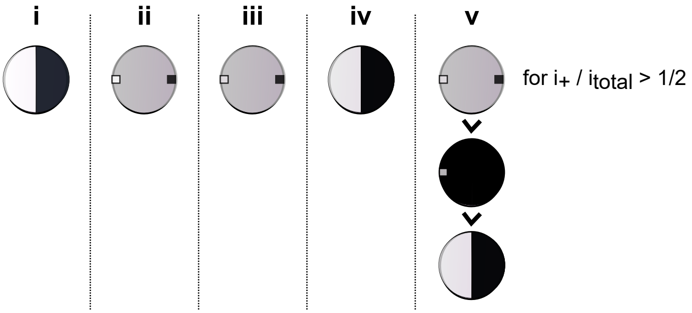

Fig. S11 Design for cells of highest possible value of P_n for different normalization conditions (independent of diameter normalization option, as isotropic in this example. For anisotropic example, you need to choose the biggest distance between high and low intensity spots.) The Latin numeration corresponds to charge normalization options i) q_{n1} , ii) q_{n2} , iii) q_{n3}, iv) q_{n4} , v) q_{n6}.

Table S2 Normalization options. All implemented normalization options of CellDetail.

Normalization options	
average to 1	'Cell average intensity normalized to 1'
none	'average to 1': unchecked
q_{n1}	'1) Charge normalized dependent on volume of cell (bit)'
q_{n2}	'2) Charge normalized dependent on protein distribution of cell (bit)'
q_{n3}	'3) Cell charge normalized corresponding to maximal value of charge multiplied by number of positive charges'
q_{n4}	'4) Cell charge normalized corresponding to maximal value of charge multiplied by halved number of total charges'
q_{n6}	'6) Own max for each cell (else: dependent on bit)'
R_{n1}	'1) Normalized by maximal diameter'
R_{n2}	'2) Normalized by averaged diameter'

values for normalized charge, and the maximal possible charge depends on bit depth (e.g. for 16 bit the value is $2^{16} - 1$). When going with the cell-based option, the focus is more on the individuality of cells and the maximal possible

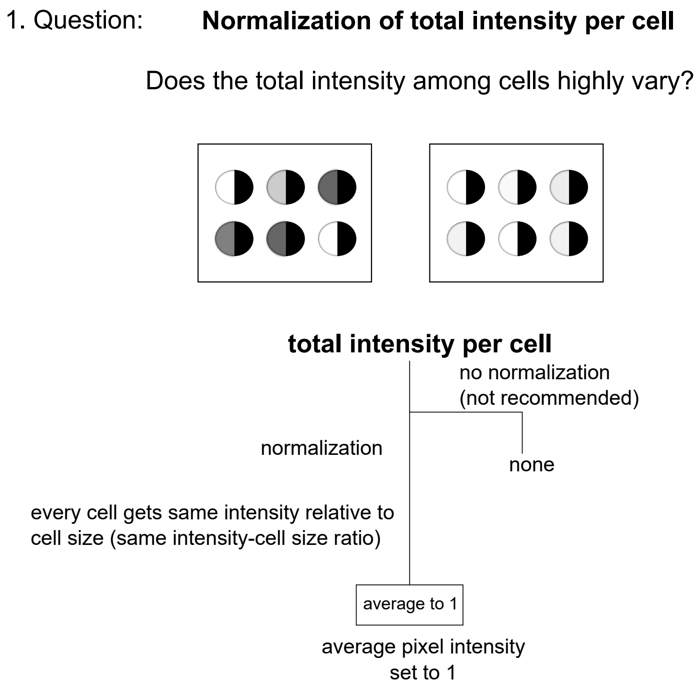

Fig. S12 Normalization choices. 1. Normalization of total intensity per cell.

charge depends on the maximal charge value found for the component of an individual cell. The capabilities of cells to cluster components more or less efficiently as well as heterogeneous vs. homogeneous distribution of cluster density play a role on the outcome for the cell-based options. The last charge normalization option (vi) is based on the percentage of the initial intensity of positive charge voxels from the total intensity. This method is again centered on single cell intensity, but with less influence of single high intensity noise voxels. Furthermore, the component content can be normalized to the average cell voxel intensity value of 1. Especially for the bit-based normalization methods, same staining and measurement protocols are critical to be able to compare data. Other normalization methods are more robust to proportional changes in intensity. Schemes for cells yielding highest polarity values are shown in Figure S11 for the different charge normalization possibilities.

Fig. S15 shows the normalization options that were chosen for polarity evaluation of the image data within this manuscript: For the analysis of polarity in this manuscript, the combination of the normalization possibilities maximal diameter for distance, bit-based (cell size) for charge and average intensity set to 1 for protein content was chosen.

In more detail, for normalization of the distance between charge centers to account for differences in cell size, two possibilities are included: A normalization by i) the maximal diameter of the cell (R_{n1}) and ii) the averaged diameter

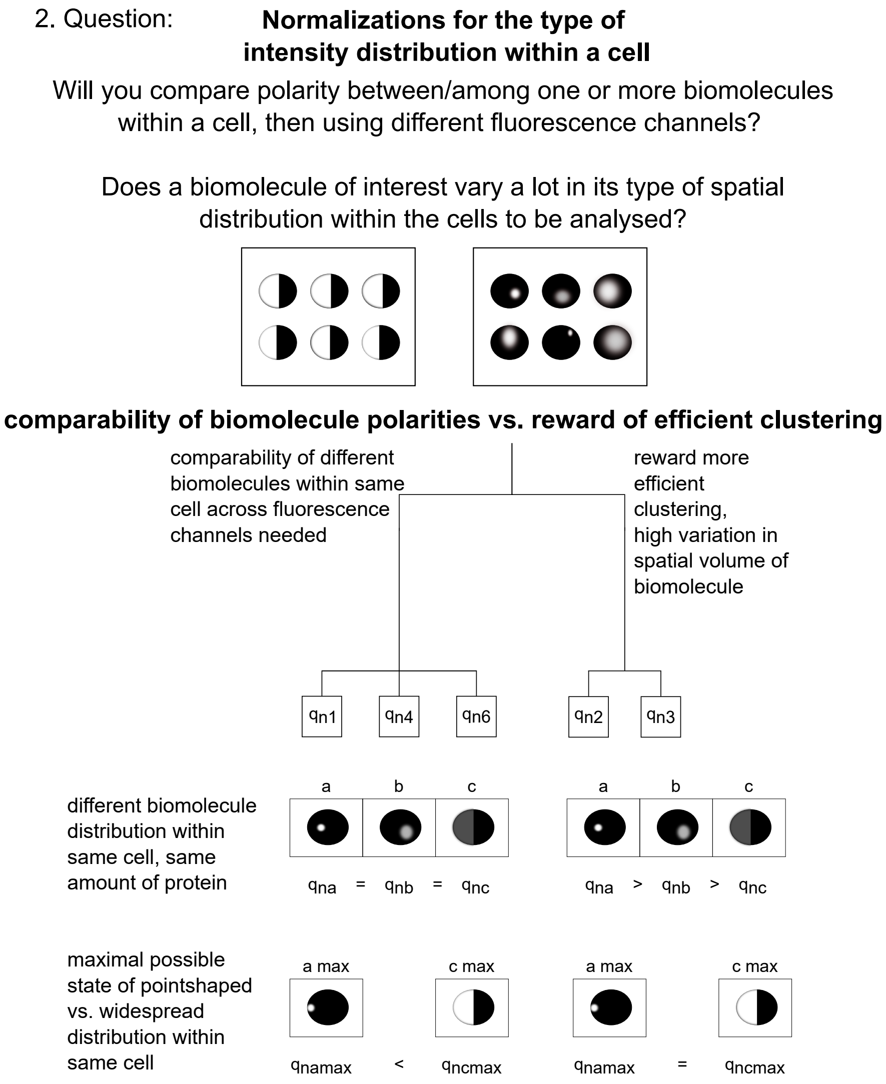

Fig. S13 Normalization choices.
2. Normalization for the type of intensity distribution within a cell.

of the cell (R_{n2}) (see Fig. S9 and Fig. S14 question 4). For the decision of distance normalization via maximal or averaged diameter, the spatial distribution of the biomolecule of interest (protrusions/elongations/uropods vs. middle of cell vs. everywhere) and the morphology of cells (anisotropic vs. isotropic) needs to be taken into account. For a biomolecule sitting at protrusions/elongations/uropods, the maximal diameter normalization is recommended, both in anisotropic and isotropic cell types. For a biomolecule sitting at the middle of

the cell, and the cell being of anisotropic morphology, the average diameter normalization is recommended. For a biomolecule being distributed everywhere in anisotropic cells, the maximal diameter normalization option is recommended. For proteins being localized in the middle of an isotropic cell or everywhere within an isotropic cell, both normalization options can be taken.
CellDetail provides 5 possibilities for charge normalization to account for variations in the parameters that influence charge (see Fig. S10). In the following equations, q_{nx} is the normalized charge with x being the normalization option used (1-6), q_{pos} is the positive charge of individual voxels, n_{voxel} is the number of voxels of cell volume, $i_{background}$ is the averaged intensity of background voxels, $i_{average}$ is the averaged intensity of cell voxels, $n_{pos,voxel}$ is the number of positive charge voxels and $q_{pos,max}$ is the maximal positive charge of cell voxel found in the individual cell.

q_{n1} is a charge normalization option that is based on bit depth. The maximal possible charge is defined by the bit depth used to store single voxel values (2^{bit}-1). Half of the cell volume is taken as maximal possible volume of positive charge. As the reference point of cell volume stays the same across biomolecules of interest, the polarity of different biomolecules within a cell can be compared across channels (see Fig. S13, comparability of polarity among biomolecules). Images need to have the same bit depth for analysis, as CellDetail recognizes automatically the bit depth of images (see Fig. S14 question 3).

$$q_{n1} = \frac{\sum q_{pos}}{0.5 \cdot n_{voxel} \cdot (2^{bit} - 1 - i_{background} - i_{average})}.$$

q_{n2} is a charge normalization option that is also based on bit depth. Only the number of positive charge voxels is taken for defining the maximal possible charge of the cell for the component/protein instead of half of the cell volume. The rationale behind this is that due to different spatial distributions of biomolecules, like more pointed distribution versus more widespread distribution, biomolecules of pointed distribution have a disadvantage as the maximal possible spatial distribution state that they can achieve shows per se a lower polarity as the maximal possible spatial distribution state that more widespread biomolecules can achieve (see Fig. S13, lower part with maximal possible state of pointshaped vs. widespread distribution). I.e. although a component might have a polar distribution, it could still be outcompeted when comparing it with a more widespread distribution of a biomolecule, but less polar distributed. This is adjusted for by individualizing the possible maximal value of charge per biomolecule by exchanging half of the cell volume (0.5 n_{voxel}) with $n_{pos,voxel}$.

$$q_{n2} = \frac{\sum q_{pos}}{n_{pos,voxel} \cdot (2^{bit} - 1 - i_{background} - i_{average})}.$$

q_{n3} is a charge normalization option that is based on the maximal positive

charge value found in the individual cell per measured fluorescence channel (see Fig. S14, question 3 with individual cell maximum). The rationale behind this normalization option is that images to be analyzed might not have the same bit depth. With this normalization the normalized charge values are not in the low value range as for the bit depth dependent normalizations (q_{n3} range: 10^{-2}-10^{-1}). A drawback is that cluster intensities need to be homogeneous (see Fig. S14 question 3).

$$q_{n3} = \frac{\sum q_{pos}}{n_{pos,voxel} \cdot q_{pos,max}}$$

q_{n4} is a charge normalization option that is based on the maximal positive charge value found in the individual cell per measured fluorescence channel (see Fig. S14 individual cell maximum). The voxel number of half of the cell is used and thus the maximal possible polarity is related to cell volume. Thus, biomolecule polarities are comparable (see Fig. S13).

$$q_{n4} = \frac{\sum q_{pos}}{0.5 \cdot n_{voxel} \cdot q_{pos,max}}.$$

q_{n6} is a charge normalization option that recalculates the proportion that the original intensity of positive charge voxels has in regard to the total intensity. This is a normalization option which is not dependent on bit depth and does not need the maximal possible value of charge per cell, but offers comparability of biomolecule polarities (see Fig. S13 question 2). Thus, higher values of q_n are reached than for the bit depth dependent normalizations, but this method is as well more robust to cluster intensity heterogeneity compared to the charge normalization options relying on individual channel charge maximal values for deriving the charge normalization (see Fig. S14 question 3 cluster intensity distribution does not matter). It is the normalization which is the furthest apart from the dipole moment concept due to the transition from charges to intensities.

$$q_{n6} = \frac{\sum q_{pos} + (i_{average} \cdot n_{pos,voxel})}{n_{voxel} \cdot i_{average}}.$$

Output files of CellDetail are .txt, .xls and .mat files, which are organized in a chosen folder, with individual output parameter files. Further outputs are .png cell detection images per channel, cell mask tiff file, cluster analysis output like angle histograms and images, a list of positive charge voxel values and positions, the mean position of a cell, the positive and negative charge-weighted center positions. Parameters like positive charge density per positive charge voxel or the angles and distances between positive charge centers of

different components can be calculated based on these output parameters. A list and description of output parameters (about 40) is listed in the CellDetail manual (Supplementary file E Manual of CellDetail).

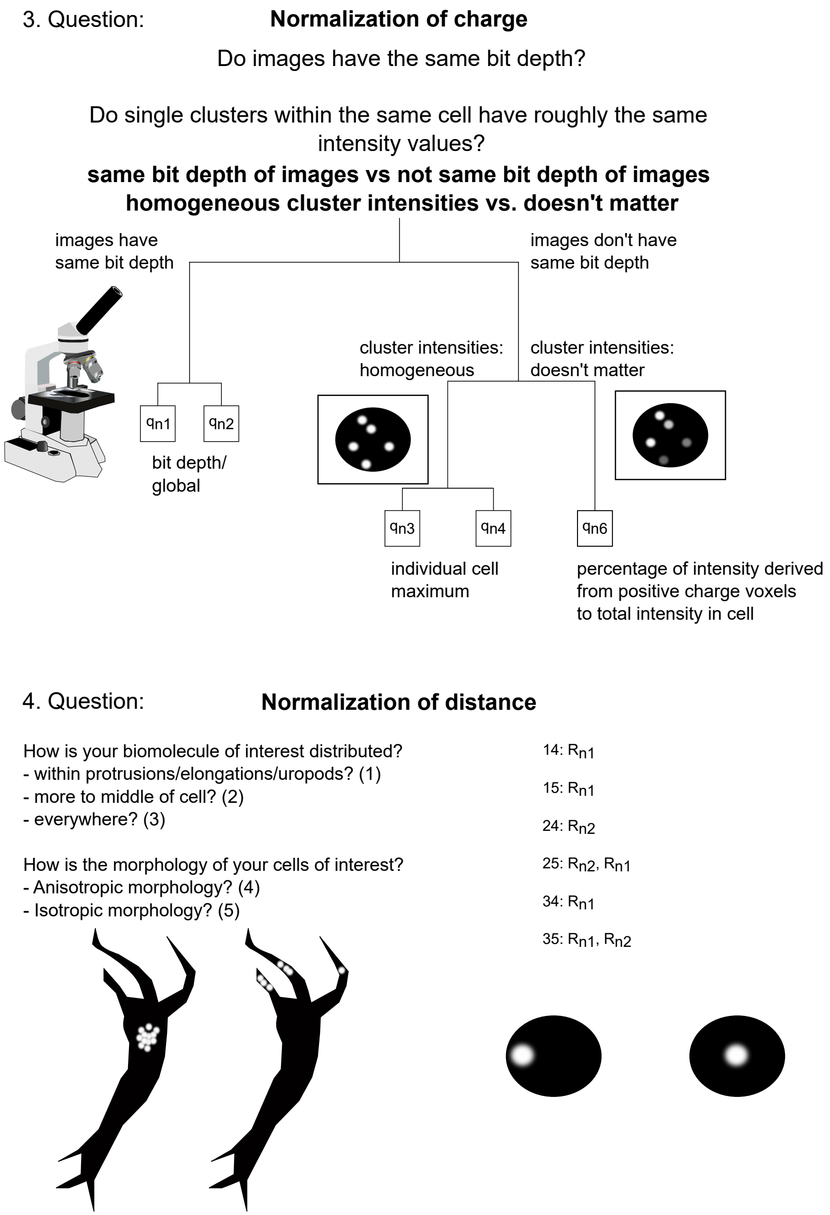

Fig. S14 Normalization choices.
3. Normalization of charge.
4. Normalization of distance between charge centers.

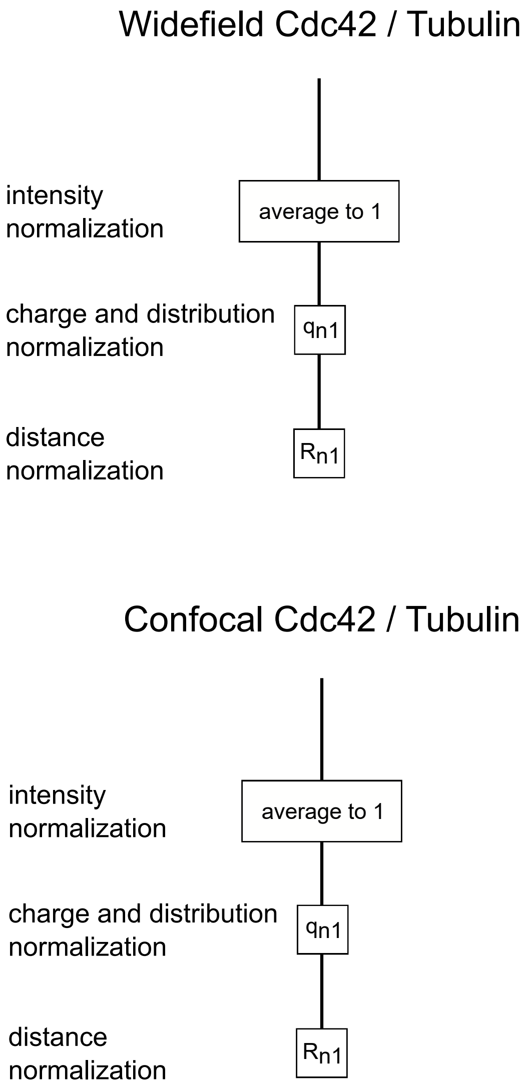

Fig. S15 Normalization choices for widefield and confocal microscopy image analysis for Cdc42, Tubulin and Septins in HSCs and FF95 cells.

Appendix G Validation of code - example scenario

For validation of correctness of code a sphere of 5000 nm radius was taken as a cell object and the circular, in radius differing mask in each layer was calculated using voxel sizes of 110 x 110 x 750 nm^3 with an assumed 16 bit image stack. The resulting z-stack can be seen in Figure S16i.

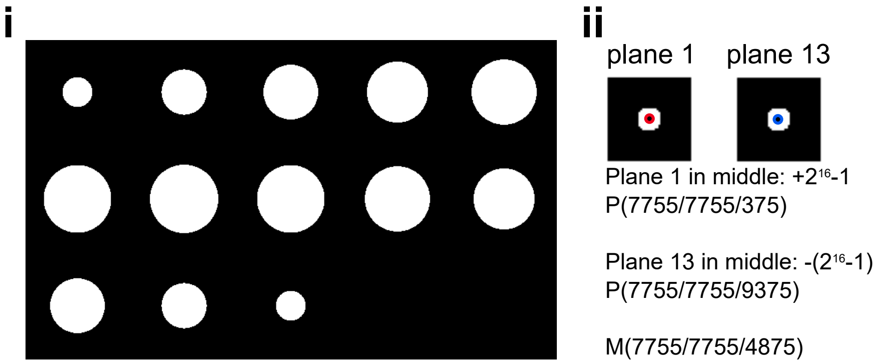

Fig. S16 (i) Mask before being filled with positive and negative charges for validation of code. (ii) First situation with positive charge at position P(7755/7755/375) on plane 1 and negative charge at P(7755/7755/9375) on plane 13. Mean position is at M(7755/7755/4875).

Different scenarios of introduced charges were evaluated. For simplicity, total charge was aimed at 0 so no subtraction of average intensity value changed the positions of positive and negative charges which facilitated the calculation of the analytical solution. Analytical solution was checked against algorithm solution. Therefore, the positive charge density q+, the normalized charge q_n, the distances between mean point of sphere and negative charge-weighted center ($|\mathbf{MR}_-|$) or positive charge-weighted center ($|\mathbf{MR}_+|$) and the distance between positive charge-weighted center and negative charge-weighted center ($|\mathbf{R}_+\mathbf{R}_-|$) as well as the absolute value of normalized distance R_n, the dipole moment vector $\mathbf{P}$, the absolute value of normalized dipole moment vector P_n with according normalization option of distance as first subscript number and according normalization option of charge as second subscript number, and the number of positive charge voxels $n_{pos,voxel}$ were compared. The first example scenario is shown in Fig.S16ii. Further detail and more example scenarios, on all normalization possibilities, can be found in the Supplementary information file. The results for the example scenario are shown in Tab.S3.

As can be seen in the first example scenario the values expected by analytical solution are in agreement with the algorithm values. In few other scenarios only small non-disturbing deviations could be seen due to: (i) Moving from continuous space (sphere) to discrete space (voxels) (averaged diameter, anisotropic behavior in different multiple positive charge settings on surface,

Table S3 First scenario for validation

Parameter	q_+ ($\frac{1}{nm^3}$)	$\vert\mathbf{MR}_-\vert$ (nm)	$\vert\mathbf{MR}_+\vert$ (nm)	$\vert\mathbf{R}_+\mathbf{R}_-\vert$ (nm)
Analytic	$\approx 7.2 \cdot 10^{-3}$	4500	4500	9000
Algorithm	$\approx 7.2 \cdot 10^{-3}$	4500	4500	9000
Parameter	$n_{pos,voxel}$	R_{n1}	R_{n2}	q_{n1}
Analytic	1	0.9	0.9	$\approx 3.5 \cdot 10^{-5}$
Algorithm	1	0.9	≈ 0.91	$\approx 3.5 \cdot 10^{-5}$
Parameter	q_{n2}	q_{n3}	q_{n4}	(q_{n5})
Analytic	1	1	$\approx 3.5 \cdot 10^{-5}$	$\approx 3.5 \cdot 10^{-5}$
Algorithm	1	1	$\approx 3.5 \cdot 10^{-5}$	$\approx 3.5 \cdot 10^{-5}$
Parameter	q_{n6} [1]	P_{n11}	P_{n21}	P_{n12}
Analytic	not practical	$3.11 \cdot 10^{-5}$	$3.11 \cdot 10^{-5}$	0.9
Algorithm	Inf	$3.11 \cdot 10^{-5}$	$3.14 \cdot 10^{-5}$	0.9
Parameter	P_{n22}	P_{n13}	P_{n23}	P_{n14}
Analytic	0.9	0.9	0.9	$3.11 \cdot 10^{-5}$
Algorithm	≈ 0.91	0.9	≈ 0.91	$3.11 \cdot 10^{-5}$
Parameter	P_{n24}	(P_{n15})	(P_{n25})	P_{n16} [1]
Analytic	$3.11 \cdot 10^{-5}$	$3.11 \cdot 10^{-5}$	$3.11 \cdot 10^{-5}$	not practical
Algorithm	$3.14 \cdot 10^{-5}$	$3.11 \cdot 10^{-5}$	$3.14 \cdot 10^{-5}$	Inf
Parameter	P_{n26} [1]			
Analytic	not practical			
Algorithm	Inf			

[1]Not practical as total intensity equals 0.

in range from $10^{-14}\%$ of up to 10% deviation from expected value). (ii) Limits of saving space of single numbers ($\ll 10^{-14}\%$). (iii) Change from double to int32 (0.1%). The first cause is in the nature of experimental images and thus intrinsic to data acquisition. The smallest deviations come from the second cause, which steps in in case of irrational numbers with unlimited decimal digits. The second cause is again intrinsic to data acquisition. The third cause however is specific for the validation of the algorithm and artificially set-up scenarios and will not occur with experimental data. All in all, the analytical and algorithm results were in good agreement.

Appendix H Manual of CellDetail

H.1 General Description of CellDetail

The main aim of CellDetail is to offer users spatial distribution / polarity quantification possibilities for components within cells (proteins, mRNAs, epigenetic marks, organelles, ...) of interest. Additional benefits are the multichannel analysis possibilities as well as the characterization of general cell and component properties. It is a versatile tool for studying multicomponent networks.
It is possible to detect cells in single tiff images and tiff z-stacks via thresholding for cell layers and thresholding for cell voxels. Channels can be left out or included to the user's liking. The resulting cell vs. background mask can be exported. Furthermore, montage images of the resulting cell mask multiplied with protein intensities can be exported. There is the possibility to exclude cells which have not been detected well, to change cell detection settings for single cells to improve cell detection, to leave out a channel of a specific cell. The exported data can be found in .txt and .xls files, the possibility to export .mat files is included.
The results consist of spatial distribution -/ polarity-related values like the dipole moment vector **P**, absolute value of normalized dipole moment P_n, normalized distance R_n and normalized charge q_n, but as well total voxel number (being related to the volume of the cell), the maximal and averaged diameter, the average intensity and total intensity as well as the index of left out cells as well as the cell detection parameters used to make it possible for the user to reanalyze a data set pretty quickly with new analysis options with the same cell detection settings.

If not satisfied with the implemented cell detection, users can refer to self-made cell masks. CellDetail's cell detection algorithm identifies one object per image file and thus is set-up for single cell images. Thus, for using CellDetail for analyzing images with multiple cells (like e.g. cell layers) the direct import of multi cell images is not recommended. Instead, the option of importing pre-made cell masks can be used with single cell objects per file and the same number of files for both tiff data files and mask files.

H.2 Fast Start

After installation of CellDetail, prepare folders per project that you want to analyze: One for tiff-images to be analyzed, another one for data and another one for generated images upon analysis. When pre-made cell masks are used as input, generate an additional folder for the mask files. When starting CellDetail, the first Tab "Import and Export settings" is shown. If not already checked, click: "See all parameters at once." Copy and paste the pathways to the folders into the according fields ("Pathway to Data Folder" for tiff images,

"Pathway to save images" for the images generated upon analysis and "Pathway to save data" for the output data; if pre-made cell detection masks are used under the "Data set rerun options" setting parameters, check "Predefined mask import option" and paste the pathway to the folder of pre-made cell detection masks into the field after "Pathway to Mask Folder" field and choose the word found in every cell detection mask file name, for which "Mask" is pre-set, by replacing it.) or use the browse function for it by checking the boxes. As tiff images are used, the word in every file name is chosen to be ".tif " unless another definition of tiff images being used is wished for. Thus, all files which have ".tif" in their name within the pathway to data folder are chosen as input files. Next name the channels of your tiff images in the field "Name Channels". As an example: When having the channels in the following order: channel 1 Cdc42-594, channel 2 Tubulin-488, channel 3 DAPI and channel 4 brightfield, one can write "Cdc42, Tubulin, Dapi, none". Thus, the channels will be named accordingly and the brightfield channel will not be analyzed. The enumeration starts with the first measured channel. It is important to name every channel, as otherwise the separation in channels will not be appropriate. The option which channel shall be taken for cell detection can be used in tab 1 (in tab 2 a more versatile variant can be used for which several channels can be taken into account for cell detection).

Write in measurement settings the pixel width x, pixel height y and voxel depth z in nm. When using images of 14 bit size, you can check 14 bit. For all bit values however, the import recognizes the bit depth of the first image and takes it. This is important when you want to use the charge normalization options 1 and 2, which rely on the bit depth.

For detection option, check "Confocal" option. This smooths inner holes of cell detection layers. For analysis options, leave "Take all parameters" and "Cell detection separated" unchecked ("Take all parameters" calculates cluster-related parameters taking more computing time, "Cell detection separated" changes the order of cell detection and analysis to a complete cell detection before calculating parameters which is advantageous when single cell calculation time takes long). "Data set rerun options" can be ignored, they only apply when reanalysis of already analyzed data shall be performed or pre-made cell detection mask tiffs shall be used. For the latter case, one needs to make sure that masks and tiffs follow the same order in both folders (extra mask folder should be made, number of tiff files needs to equal number of mask files, size of mask file needs to equal size of corresponding tiff file).

In the second tab "Adjust Parameters and Pre-View of cell detection" the "Parameters for cell detection" are set for a general run-through. (Later in the third tab, cell detection can be improved per single cell.)

First, the layer thresholding is performed. Usually, the "Basic Parameters" subtab should be enough for detecting cells of interest. For giving users more freedom, additional parameters can be varied under the subtab "Additional Parameters". For applying thresholding at all, "Apply thresholding" needs to be checked (below "General Parameters" box). Otherwise, only the whole

image stack of a channel of a single cell can be looked at. The channels which shall be used for cell detection can be given freely when checking “Several channels for cell detection” at the lower base and replacing the input “1,2,3” with the wished channels. As some channels can appear really bright, the program sets the maximum of each channel to be the same. For weighting of channels for cell detection, one can check “Weighting of channels” and write in the weighting of each selected channel. For first adjustment, it makes sense to check both “Apply thresholding” and “Show just layer thresholding”. The first step of detection is performed: The layer thresholding. Two parameters can be set in the “Basic Parameters” option: “Minimum number of pixels per layer for Range filter” and “Maximum number of pixels per layer for Range filter”. By varying and clicking through the cells (caveat, one needs to click through in order to see effect) via the “Back” and “Next” buttons below the image montage on the right, the new settings are adapted and one can optimize the values. Nonetheless, via the “Minimum number of images per cell (+/-1)” and “Maximum number of images per cell (+/-1)” options one can have additional influence on how many layers are at minimum and at maximum taken. When the layer thresholding was optimized, next comes “Cell pixel thresholding” and one unchecks “Show just layer thresholding”. Cell pixel vs. background thresholding in the chosen layers is mostly performed via “Imbinarize Threshold for Confocal” under general parameters.
As fluorescence light in widefield condition already leads to signal outside of the cell interior in a nearly uniform manner, one has the option to check “Advanced” option next to “Parameters for cell detection” and “Take in account: out of focus” to move layers consistently by a certain value. E.g. when signal comes already two layers before the cell itself appears, a 2 can be written for “Reposition of layers along depth: Move found layers by value” parameter. The detected layers will always be moved by 2 layers. Negative values are allowed as well for repositioning.
When cell detection does not work with the pre-set parameters, one can choose “Program Run: Visible cell pre-view making?” (option below “Apply thresholding” and “Show just layer thresholding” option at the base). This allows to observe the cell detection algorithm at work. One can see at first the original image versus the range filter, afterwards the decision on layer thresholding and the decision on cell pixel thresholding. Thus, it is possible to conclude which cell detection step still needs improvement.
For the shown image montage, you can select the channel which shall be shown (“Number of channel to show”). If you want to have an easy possibility to rerun analysis on the data set, you should check “Save images inclusive mask (latter as tif-stack)” as this leads to output masks which can be used for the rerun functionality in the first tab (be careful when going for rerun in this case if you selected cells not to be taken into account for analysis due to poor cell detection, as the cell original image files and mask files need to be removed for reanalysis then).

You will receive results for the selected normalization possibilities in the drop-down menu (pre-set: “1) Normalized by maximal diameter” and “1) Charge normalized dependent on shape of cell (bit)”). Check “Cell average intensity normalized to 1” (cells’ protein amount variability under different conditions shall not have influence) and if co-localization is an important parameter for your experiment, check “Pearson correlation coefficient” option. When the general settings are optimized for your image data, click the “Start run” button. A window appears showing the stage of analysis. After run-through, the “Feedback to user” field will show “Runthrough done: Elapsed time is ” and mention the time needed for performing the run-through. Next you move to the third tab “Results”. On the left handside the detected cell voxels versus background voxels are shown. On the right handside a table is given with some of the output values. You can move through the table and cells via “Back” and “Next”. If cells shall be removed from analysis data, one can check “Not to take”. This can be undone. If a cell was not detected well, one can check “Rerun” (right handside, top) and a “Rerun settings” windows opens with the parameters already seen in tab 2. The previously general settings can now be adapted for the single cell. Before the adaption is completed, you can check with a “Preview of Rerun” click whether cell detection is good. When cell detection is good, the “Rerun (table values changed)” button needs to be clicked in order to adapt values with optimized cell detection settings. After moving through cells and checking cell detection, you can export data by clicking “Export Data” button. The recommendation is to export .mat files as well (check “.mat export”) as another rerun option for reanalysis is possible with it. The output values are now saved in the data folder as .xls files, .txt files and .mat files (if option for it was checked). You obtain the following values:

- averaged intensity (value, which is changed when normalization of intensity is performed (total to 1, average to 1))
- “Chargedensitypos”, total positive charge density (a.u./nm^3, not per voxel)
- dipole moment $\mathbf{P}$
- averaged and maximal diameter
- normalized charge q_n(various options)
- normalized dipole moment P_n (various options)
- normalized distance between charge centers R_n (various options)
- number of pixels/voxels (cell size)
- Pearson correlation coefficient table
- positive pixel/voxel number
- vector $\mathbf{R_{neg}M}$ (“RnegRmean”, $\mathbf{R_{neg}}$ negative charge weighted center, $\mathbf{M}$ middle of cell)
- vector $\mathbf{R_{pos}M}$ (“RposRmean”), $\mathbf{R_{pos}}$ positive charge weighted center
- vector $\mathbf{R_{pos}R_{neg}}$ (“RposRneg”)
- total intensity (parameter which is not changed due to normalization of intensity)
- “indexnottaken” (index of cell/s not taken, if cell/s excluded from analysis)

For exported .mat files, additional information is yielded:

- “Names”, names of cell tiff files (cell names in the order of run-through)
- “allchannelnames”, channel names
- “savingofsettingsofcertaincells” (for rerun option)
- “toignore”: index of channel not taken in account (e.g. brightfield)

Caution: After export of data press the button “Next Data Folder” in tab 1 “Import and Export settings” for deleting global variables which could interfere with the new data set!

H.3 Detailed Description

H.3.1 Tab 1: Import and Export settings

The user is asked for import, export and basic information about data to be analyzed in tab 1. Roughly seven areas/panels can be drawn:

- Panel 1 for “Import”/“Export” settings as well as loading settings of another run (“Load Settings”) as well as starting over with a new dataset (“Next Data Folder”, for deletion of global variables).
- Panel 2 for “Channel information” with writing down channel names separated by comma and naming channels not being of interest for analysis as “none”. A pre-step of cell detection can be made by choosing the channel for cell detection in tab 1. However, in tab 2, several channels can be chosen together with a weighting. Thus, when several channels shall be used for cell detection (recommended), this point can be neglected till tab 2.
- Panel 3 for “Measurement settings”. The pixel/voxel values are asked for as well as whether 14 bit was used or not. This is due to ImageJ not being able to save tiff as 14 bit images, thus allowing the user for correcting mislead analysis when choosing charge normalization options 1 or 2.
- Panel 4 for “Detection option” of “Confocal” or not. For the “Confocal” option, steps are performed in order to fill holes within detected cell areas. For conventional widefield images, “Confocal” option can be an improvement as well. The “Analysis options” are called “Take all parameters” and “Cell detection separated”. For the option “ Take all parameters” additional parameters are calculated, like the clusters of positive charge voxels, their size and position. For the option “Cell detection separated” first the cell detection is made, before the analysis occurs. If not checked, when starting the run in tab 2, the analysis is performed right after cell detection of single cells leading to a results table in tab 3 giving an overview over essential parameters (normalized dipole moment, ...).
- Panel 5 for “Data set rerun options” gives facilitation for users wanting to rerun an already analyzed data set. Users can use the “savingofsettingsofcertaincells.mat” file and “indexofcellsnottaken.mat” file, thus reusing previous cell detection parameters, or use masks belonging to their image data files of interest.
- Panel 6 for user feedback.

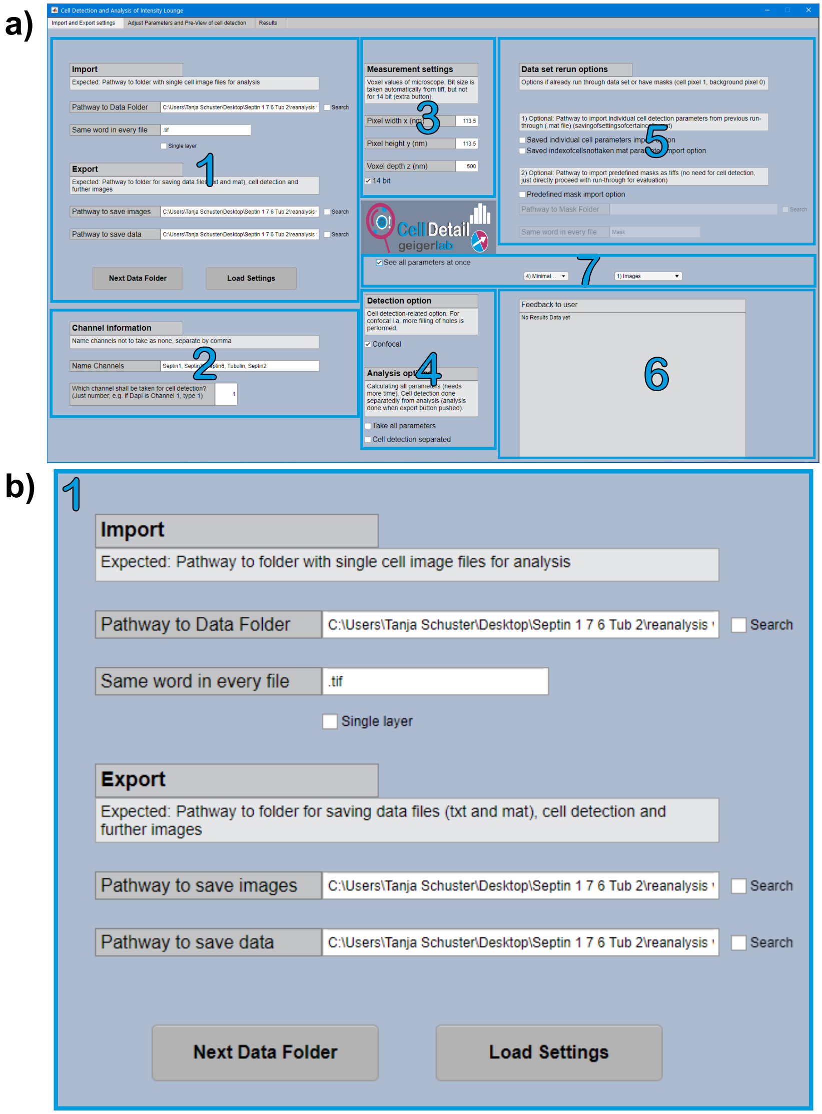

Fig. S17 a) Overview over tab 1 of GUI CellDetail b) Overview over tab 1 panel 1 Import, Export and buttons

- Panel 7 for view options of tab 1. Users can see all parameters at once ("See all parameters at once" checked), or panel 1 till panel 5 one after the other ("See all parameters at once" unchecked, with appearing "Back" and "Next" buttons), either via transparency or image panels. By "Back" and "Next" button the user can click through, but it is possible via mouse click on the next panel as well.

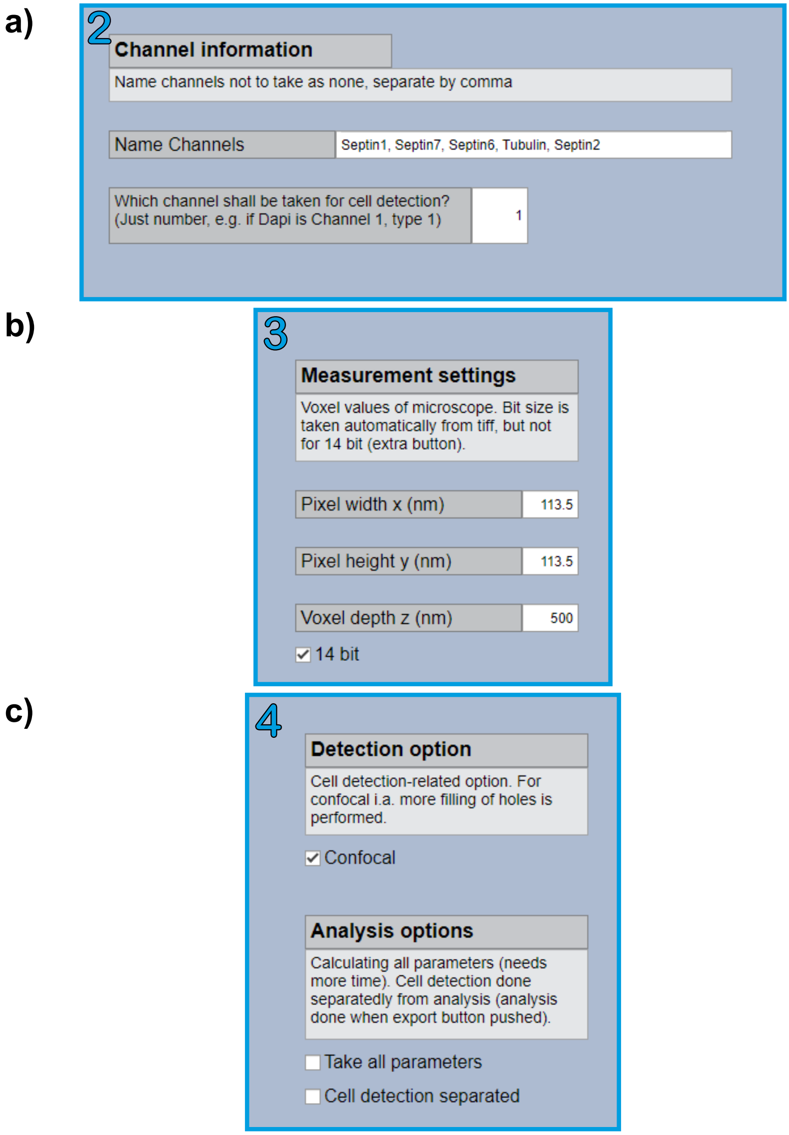

Fig. S18 a) Overview over tab 1 panel 2 Channel settings b) Overview over tab 1 panel 3 Measurement settings c) Overview over tab 1 panel 4 Detection and analysis options

Panel 1

Panel 1 of tab 1 asks for import information. The user needs to give the path of the Data Folder, in which the images to be analyzed are contained. This can be done either by copy and pasting the pathway or by checking the search

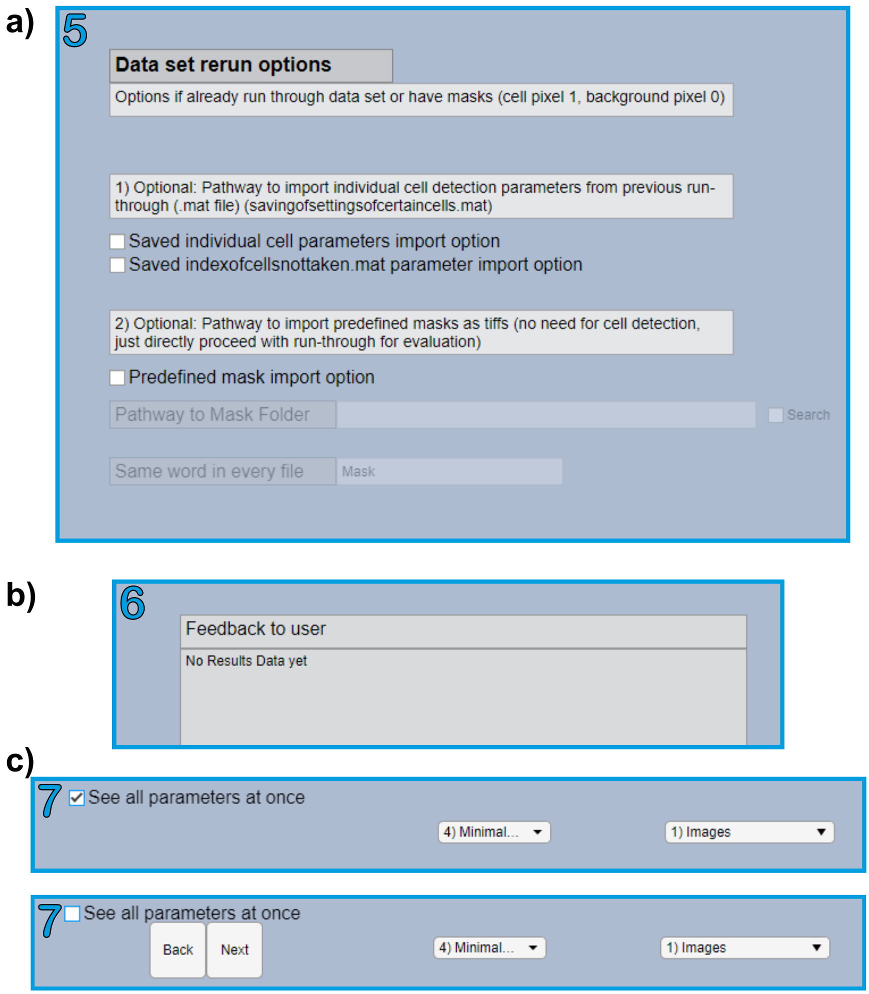

Fig. S19 a) Overview over tab 1 panel 5 Rerun settings for reanalysis b) Overview over tab 1 panel 6 Feedback field c) Overview over tab 1 panel 7 View options

button. For CellDetail receiving access to the files in the folder, the user has to choose a phrase which is contained in the file names of interest ("Same word in every file", pre-set: ".tif"). From the import pathway in combination with the phrase to look for, already the image names and image pathway names are extracted as well as the cell number.
If single images are used instead of z-stacks, the single layer button needs to be checked as later in cell detection setting, a layer number of less/equal two is considered too low and further attempts are made for detecting cells.
Export information needs to be given by choosing folders in which to save resulting images ("Pathway to save images") and analysis data ("Pathway to

save data". It is recommended not to choose the same pathway as the "Pathway to Data Folder" (import folder), to be sure that no complications occur during processing. Again, the checking of the search field at the right side gives the user the possibility to search for the folder in the system. Another possibility is copy/pasting the pathway of the corresponding folder.
When a data set was analyzed and the user switches to the next data set in a different folder, the user has to click the button "Next Data Folder", as certain global variables need to be deleted to make clean space for the new data set. This involves variables saving the analysis values relating to polarity as well as the cells that are ultimately not taken. Images that are saved in the folder under "Pathway to save images" include - when "Save images inclusive mask (latter as tif-stack)" in tab 2 panel 6 is checked - the montage of masked channel images and the tif-stack of the mask.
"Load Settings" can be used for the .txt file made when clicking "Save Settings" button in tab 2. General parameters of cell detection as well as import pathway, phrase to find in files, export images pathway, export data pathway, pixel resolution x (nm), pixel resolution y (nm), voxel resolution z (nm), number of channels, channel(s) to ignore index, channel names, channel taken for cell detection, number of images, confocal option, normalization option, several channels for cell detection option as well as used channels for cell detection, weighting option and weights and time stamp are saved in the import folder as .txt file.

Panel 2

The user names the channels in tab 1 panel 2. It is important to list all channels, even the ones not of interest like brightfield channel, as the import makes use of the channel number. Channels, that shall not be analyzed, should be called "none". The channels need to be separated by comma. By filling out this field, the naming and number of channels, the number of channels of interest as well as their order is deduced. If a channel is accidently omitted, the wrongdoing can be seen in tab 2 as channel images are intermixed in the case of stacks being analyzed.
If cell detection shall be performed using a single channel only, the value of the channel needs to be placed after the field "Which channel shall be taken for cell detection?". If cell detection shall be performed using multiple channels, this field can be ignored, as it will have no influence on the further cell detection when opting for multiple channels in tab 2 panel 2.

Panel 3

Panel 3 demands for information on measurement settings. Pixel width, height and (if in 3D) voxel depth values are asked for. When using 2D images, voxel depth should be set to 1 as charge density is calculated by dividing through the voxel volume. The pixel/voxel values provided by the user influence charge density, the absolute position of middle of volume $\mathbf{M}$, of positive charge weighted mean position R_{pos} and of negative charge weighted mean position R_{neg}, thus the distance and dipole moment values. The relative values resulting by

normalization are not changed. When importing tif, the bit number is automatically taken from the GUI. This value is used when opting for options 1 and 2 for charge normalization. As 14 bit is not offered by saving tiff images via ImageJ, and for holding the options 1 and 2 for charge normalization still open, the check button for 14 bit was inserted. Other bit values of tiff images are automatically recognized.

Panel 4

Panel 4 is structured into two main themes: "Detection option" and "Analysis options". Detection option relates to the cell detection algorithm for which a difference between confocal (low voxel width/height values) and widefield (high voxel width/height values) is made for improving cell detection. Low voxel width/height values make it necessary to take filtering steps in order to fill in areas in which several cell voxels were not recognized.

When considering widefield images, the "Confocal" option can still be an improvement and can be used.

The "Analysis options" have two possible options: "Take all parameters" and "Cell detection separated". When the "Take all parameters" option is checked, the positive charges above the mean of positive charges and their positions are further analyzed by converting voxel positions of the (x,y,z)-coordinate system into a spherical (ϕ, θ, r)-coordinate system. It was chosen to take the positive charges above the mean of positive charges, as for the confocal setting the inclusion of all positive charges for further analysis leads to not only protein voxels being taken in account but as well clear cytoplasm voxels and thus, taking the positive charges above the mean of positive charges is more precise. There exist two clustering methods: 1. Clusters are built based on histogram counts above average expected count. Their size and angle between them are analyzed. 2. Clusters out of the positive charge voxels with values above mean of positive charges are built based on the resulting nearness of positive charge voxels above mean of positive charges. Information about cluster size, angle and intensity is saved. When "Cell detection separated" is checked, the cell detection is at first performed. The analysis follows later when pushing the export button. This facilitates user interaction with the GUI as calculation of all parameters can take time and thus single cell detection correction would become tedious with the waiting times in between.

Panel 5

The "Data set rerun options" are implemented for reanalysis of data, e.g. when one wants to try out another normalization method. Herefore, one can either use the parameter "savingofsettingsofcertaincells.mat", which saves the individual cell detection parameters for each cell, and "indexofcellsnottaken.mat" (both are saved automatically when exporting .mat files). Another option is to have masks of cell voxels (with value 1) and background voxels (with value 0) corresponding to your tiff-images e.g. via the option "Save images inclusive mask (latter as tif-stack)" in tab 2 panel 6. The order of masks and tiffs within their folders must be the same. A check on the number of images and width and height is performed and acceptance is given as feedback in tab 1 panel 6.

When using these options, the user does not need to look at cell detection any more. Thus, there won't be a result table in tab 3 showing the results. Nonetheless, after inserting the necessary information in tab 1, press the "Start run" button in tab 2 and export afterwards your analysis data in tab 3 ("Export Data").
Use the "Cell detection separated" button from tab 1 panel 4 and make a run-through in tab 2 panel 6 by clicking on "Start run". After the fast run-through, go to tab 3, make a check for single cell detection and when accepted click "Export Data". After giving a name, the calculation of the parameters starts and they are saved in the corresponding folders.

Panel 6

Field for giving response to the user.

Panel 7

Dependent on the design chosen, one can either see all parameter fields at once or in a certain order. For getting to the next parameter fields, one can either use the "Back" and "Next" button seen in panel 7 or click directly on the next panel to get visible.

H.3.2 Tab 2: Adjust Parameters and Pre-View of cell detection

Tab 2 shows the parameters for cell detection. Cell detection is performed by using Otsu's method on range filtered images for layer selection and on plain image layers for cell voxel selection. The layer thresholding takes place before the cell voxel thresholding. Figure S20 shows tab 2 for "Basic Parameters" and tab 2 for "Additional Parameters" option in the subtabs. It is possible for the user to change parameters in every step of the general cell detection algorithm to make the software flexible for all kinds of image data and objects to be detected. Tab 2 can be visually separated into 6 panels:

- Panel 1 with cell detection parameters. It is in itself separated into layer thresholding, cell voxel thresholding and general parameters.
- Panel 2 with options concerning which channels shall be used for detection and with which weighting, as well as viewing options: Just to see how thresholding process looks like, one checks "Program Run: Visible cell preview making?". The according images appear in a separate windown. If you just want to look whether the right layers are chosen with no further processing of thresholding, you check "Show just layer thresholding" and "Apply thresholding" and see the result in panel 5. When you want to see the result of all thresholding steps, just check "Apply thresholding" without further options ("Show just layer thresholding", "Program Run: Visible cell pre-view making?") selected and the result can be seen in panel 5.
- Panel 3 with the button "Save Settings". This button helps in saving the general cell detection settings for reuse in other experiments. They are saved in a .txt file.

- Panel 4 as feedback field. When a cell object can't be generated due to settings like maximum number of voxels for each cell etc., a feedback will go out for the user.
- Panel 5 as resulting image field.
- Panel 6 as a control field for moving through the images and visual adaptations (e.g. for channel to be visualized,...) and options for analysis (normalization options, saving options, Pearson correlation coefficient) with the "Start run" button to start cell detection and/or analysis. The options "Calculate all normalization possibilities" and "Cell total intensity normalized to 1" were removed as they are not needed for the typical use case.

Panel 1

In panel 1 under subtab "Basic Parameters" the most often changed parameter fields can be seen. The parameter fields for "Layer thresholding" and "Cell pixel thresholding" can be approached by checking "Advanced".
For "Layer thresholding", the most often changed parameter for optimizing cell detection is "Minimum number of voxels per layer for Range filter" and "Maximum number of voxels per layer for Range filter". As cells are non-homogenous objects, we make use of a range filter on the original images which gives out the maximal minus minimal intensity value in a specified neighborhood (9x9 pixels). For just background images, a low value of the range filter is expected, while for images with both cell voxels and background voxels, more variability and thus higher resulting values are expected. After the range filter, a threshold needs to be defined for separating pixels/voxels into cell pixels/voxels and background pixels/voxels. The threshold set on defining pixels/voxels as being cell voxels is made by using Otsu's threshold method on the histogram of range filtered images and adding a specified value ("Threshold addition 1: If global threshold > Threshold frontier 1", "Threshold addition 2: If global threshold < Threshold frontier 1", visible under subtab "Additional Parameters" under "Layer thresholding" parameters 5 and 6 of the row) to the threshold calculated by Otsu's method. Whether "Threshold addition 1" or "Threshold addition 2" is used depends on "Threshold frontier 1" and the threshold calculated by Otsu's method being smaller or bigger than "Threshold frontier 1".
For "Confocal" (tab 1, "Detection option") case as well as the widefield case (tab 1, "Detection option", unchecked "Confocal"), another comparison with a threshold takes place in order to get outlier cases for which due to previous parameter settings, no layers were detected.
The "Confocal" option uses "Select layers differently if mean of voxel number is bigger than this value: depending on being bigger than mean value rather than on minimum value and Minimum number of pixels per layer for Range filter". This means that when the mean number of voxels selected as cell voxels per layer (thanks to Otsu's threshold method with corresponding threshold addition decisions before) is bigger than the set value (pre-set: "5e+04") layers

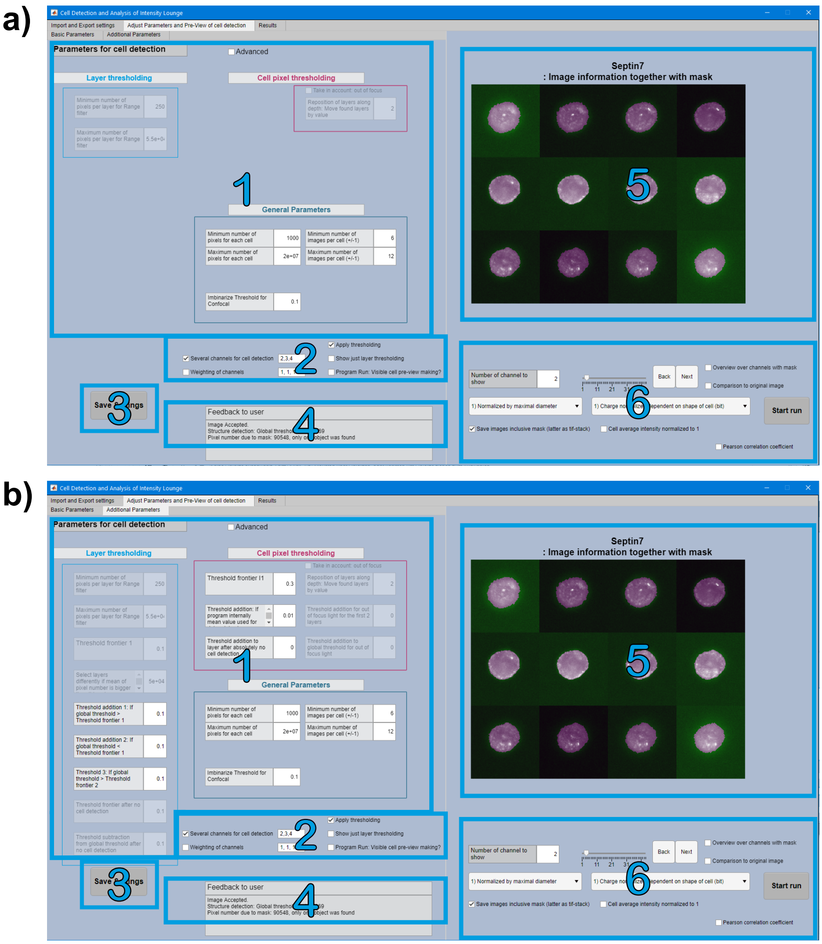

Fig. S20 a) Overview over tab 2 with basic parameters b) Overview over tab 2 with advanced parameters (see change in panel 1)

are selected for being cell layers when their number of defined cell voxels is above the mean value of defined cell voxels per layer.
The not-“Confocal” option (widefield) uses the parameters “Threshold frontier 2” and “Threshold 3: If global threshold > Threshold frontier 2”. It takes a global threshold calculated by Otsu’s method (calculated on histogram over all layers, not per layer) and compares it to “Threshold frontier 2”. If the global threshold is bigger than ‘´Threshold frontier 2”, a new threshold is assigned

for separating pixels/voxels as being cell pixels/voxels ("Threshold 3", preset "0.15"), otherwise the global threshold is used. Ranges of layers are taken which show a high enough cell voxel number. If the result is 0 layers and widefield was chosen (= "Confocal" option in tab 1 was not chosen), another approach is taken by applying an average filter of neighborhood 9x9 before the range filter step. Otsu's threshold method is performed on the resulting image. The resulting Otsu threshold is changed by subtraction according to it being above or below a certain threshold (certain threshold: "Threshold frontier after no cell detection", subtraction: "Threshold subtraction from global threshold after no cell detection").
If after all these steps no layer could be detected, the feedbackfield shows the text "No cell detected: Please try other values for setting Structure threshold". Afterwards the separation is made between the cases one layer and more than one layer. If the "Single layer" option in tab 1 panel 1 was not checked, the longest episode of layers is looked for and repositioning of layers takes place (parameter found under "Cell pixel thresholding" for the selected option "Take in account: out of focus"). Further limits of layer number are applied ("General Parameters" under "Minimum number of images per cell (+/-1)" and "Maximum number of images per cell (+/-1)") . Thus, the result of "Layer thresholding" is a range of selected layers, which go further to "Cell pixel thresholding".

The same thresholding steps are made for both single and more layers for defining cell voxels vs. background voxels (Cell voxel thresholding):

For the widefield case the special handling of the first 2 layers was implemented due to light shining through from other layers while the interior of the cell was not reached in the layer. It can be activated by checking "Take in account: out of focus" (parameters taken into account when selected: "Reposition of layers along depth: Move found layers by value", "Threshold addition for out of focus light for the first 2 layers", "Threshold addition to global threshold for out of focus light"). Herefore, Otsu's threshold method is used on all selected layers and single layers. By comparison of single layer Otsu threshold and global (across all layers) Otsu threshold with added "Threshold addition to global threshold for out of focus light" (together with the condition of the layers being below 3 and the option for taking out of focus light being selected (check of "Take in account: out of focus")), the decision is made whether the resulting threshold is single layer Otsu's derived threshold added to "Threshold addition for out of focus light for the first 2 layers" (yes, if single layer Otsu threshold is bigger than global layer Otsu threshold added to "Threshold addition to global threshold for out of focus light"). If single layer Otsu threshold is smaller than global layer Otsu threshold added to "Threshold addition to global threshold for out of focus light" for the first two layers, another comparison of single Otsu threshold being bigger than "Threshold frontier I1" is made. If it is bigger, then the resulting threshold is single layer Otsu's threshold added to "Threshold addition 1: If layer threshold > Threshold frontier I1". Otherwise, the resulting threshold is equal to single layer Otsu

threshold. The part with Threshold frontier I1 comparison is always used for layer numbers above 2 and if “Take in account: out of focus” is not checked, it is used for the layers 1 and 2 as well. The number of cells voxels per layer is checked against the maximal and minimal allowed pixel/voxel numbers per layer (“General Parameters” with “Minimum number of pixels in layer” and “Maximum number of pixels in layer”). A layer is set to zero if it does not meet the criteria. If the number of valid layers is below 4, another method is used for defining cell layers. Dependent on the found valid layers from previous thresholding, single layer Otsu threshold is used with addition of 0.1 (for valid number of layers <4) or with addition of “Threshold addition to layer after absolutely no cell detection” (for valid number of layers == 0). If the resulting number of cell pixels/voxels is below the minimal number of pixels to detect for a cell or no valid layer was found, the threshold is set to global Otsu threshold + 0.1 (if valid layer number is below 4), to global Otsu threshold + 0.04 (if valid layer number is equal to 0) or layers below minimal number of pixels per layer and layers above maximal number of pixels per layer are deleted (if valid layer number is above or equal 4). Afterwards, holes are filled and cell pixels/voxels are merged to a single object taking into account minimal and maximal pixel/voxel number allowed per cell.

For the confocal case, Otsu’s threshold method is applied on all images. If for layer thresholding the method of having higher pixel/voxel number than mean pixel/voxel number per layer was used, the resulting threshold for splitting into cell pixel/voxel and background pixel/voxel is calculated by addition of “Threshold addition: If program internally mean value used for determining layers” to the global Otsu method threshold. If not, the global Otsu method threshold is taken. Afterwards, the images are filtered with a Gaussian filter of size 11x11 and the “Imbinarize Threshold for Confocal” is taken for final imbinarizing into cell pixel/voxels and background pixel/voxels.

Afterwards the holes of the cell mask are filled (with more operations on filling for the confocal case) and a mask object is made. The biggest mask object below the maximal voxel number is taken as a cell mask.

As can be seen, some threshold parameters can only be approached via the decision on the parameter “Confocal” in tab 1. Starting values are defined according to condition “Confocal” being checked or not. Several parameter names change upon selection of confocal:

- “not Confocal” / “Confocal”
- Threshold frontier 2 / Select layers differently if mean of voxel number is bigger than this value: depending on being bigger than mean value rather than on minimum value and Minimum number of voxels per layer for Range filter
- Threshold addition 1: If layer threshold ¿ Threshold frontier I1 / Threshold addition: If program internally mean value used for determining layers
- Minimum number of pixels in layer / -
- Maximum number of pixels in layer / Imbinarize Threshold for Confocal

a)

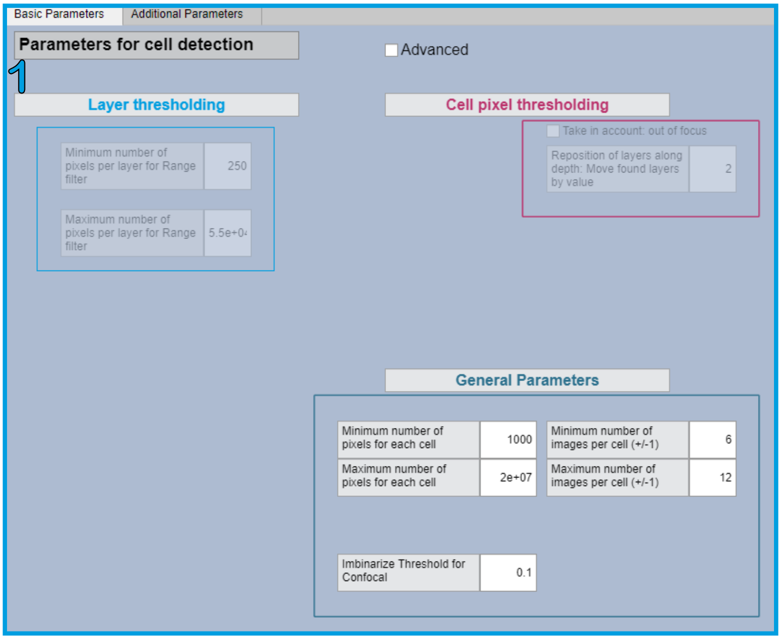

Fig. S21 a) Overview over tab 2 panel 1 with "Basic Parameters"

Panel 2

Panel 2 holds the controls on cell detection being carried out on not just a single channel, but selected channels. Herefore, the intensity values are added, and if wanted with a special weighting of channels (weighting done by multiplication). Without weighting, the values are - just for mask decision - adapted to the first imported channel's maximal value by changing the intensity ranges of the other channels. For visual information on the output of cell detection,

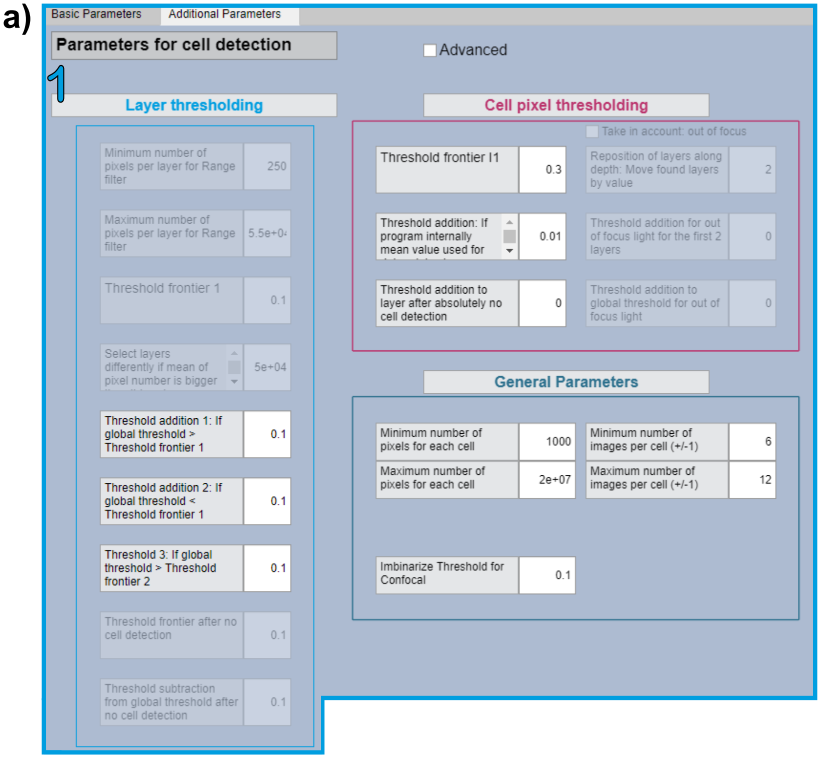

Fig. S22 a) Overview over tab 2 panel 1 with "Advanced Parameters"

the options "Apply thresholding", "Show just layer thresholding" and "Program Run: Visible cell pre-view making?" can be chosen.
When "Apply thresholding" is not selected, the imported images are shown as montages, no modification is applied. You can have a check whether the images were imported correctly (e.g. when a channel name is missing, the channels' images are intermixed).
When you want to have a look at how the "Layer thresholding" (= cell layer

selection) performs, you need to check both “Show just layer thresholding” and “Apply thresholding” buttons. When you want to see the outcome of all thresholding steps defining cell voxels vs. background voxels, you need to check “Apply thresholding”.
When you want to have a look at the cell detection process, you can check “Program Run: Visible cell pre-view making?”. A separate window opens and shows the run-through of several thresholding steps per layer (1) left: original image, right: result of Range filter; 2) left: original image, right: result of thresholding Range filtered image (= result of “Layer thresholding”); 3) left: original image, right: result of “Cell pixel thresholding”, 4) left: original image, right: final mask multiplied with intensity values (= result of cell detection algorithm)). This can help in identifying bottle necks in the process for your cells' detection e.g. whether improvements need to be made for “Layer thresholding” or for “Cell pixel thresholding”.
It is recommended to optimize the parameters in tab 2 panel 1 so that most cells are well detected as these parameters are in general used for cell detection in your data set. Afterwards in tab 3 there is the option to adapt cell detection for single cells.

Panel 3

The “Save Settings” option allows the saving of the cell detection threshold parameters in a .txt file in the import pathway, which can be loaded for other experiments in tab 1 panel 3 (“Load Settings”). The saved variables are:

- pathway to import data
- phrase found in data file names of files to be analyzed
- pathway to export images
- pathway to export data
- pixel/voxel size x
- pixel/voxel size y
- voxel size z
- number of channels
- number/ of which channel/s is/are ignored
- names of channels
- number of channel chosen for cell detection
- number of data files
- Option “Confocal”: checked or not
- Option “Advanced”: checked or not
- Option “Out of focus light”: checked or not
- Threshold frontier 1
- “Threshold frontier 2” / “Select layers differently if mean of voxel number is bigger than this value: depending on being bigger than mean value rather than on minimum value and Minimum number of voxels per layer for Range filter”
- “Threshold addition 1: If global threshold > Threshold frontier 1”
- “Threshold addition 2: If global threshold < Threshold frontier 1”
- “Threshold 3: If global threshold > Threshold frontier 2”

- “Threshold frontier after no cell detection’
- “Threshold subtraction from global threshold after no cell detection”
- “Minimum number of pixels per layer for Range filter”
- “Maximum number of pixels per layer for Range filter”
- “Threshold frontier I1”
- “Threshold addition 1: If layer threshold > Threshold frontier I1” / “Threshold addition: If program internally mean value used for determining layers”
- “Threshold addition to layer after absolutely no cell detection”
- “Threshold addition for out of focus light for the first 2 layers”
- “Threshold addition to global threshold for out of focus light”
- “Reposition of layers along depth: Move found layers by value”
- “Minimum number of pixels for each cell”
- “Maximum number of pixels for each cell”
- “Maximum number of images per cell (+/-1)”
- “Minimum number of images per cell (+/-1)”
- “Maximum number of pixels in layer” / “Imbinarize Threshold for Confocal”
- “Minimum number of pixels in layer”
- “Normalization option diameter”
- “Apply thresholding”
- “Show just layer thresholding”
- “Show pre-view making”
- “Comparison to original image”
- “Normalization option charge”
- Option “Several channels for cell detection”
- “Channel numbers for cell detection”
- Option “Weighting of channels”
- Weights of individual channels
- Option “14 bit”, Tab1 Panel3
- Option “Cell average intensity normalized to 1”
- Option “Cell total intensity normalized to 1”
- Option “Single layer”, Tab1 Panel1
- date and time

Panel 4

Field offering feedback to user. Feedback is made for facilitating thresholding part.

Panel 5 and Panel 6

Panel 5 is used as a visualization field for image data as well as for found cell layers and overlaid cell detection masks. The refreshing of the field needs the pushing of the buttons “Back” and “Next” in panel 6 or the modulation of the index on the image number list axis.
The shown channel can be adapted by changing “Number of channel to show” to the number of channel you want to look at.
By checking “Overview over channels with mask” option an image containing

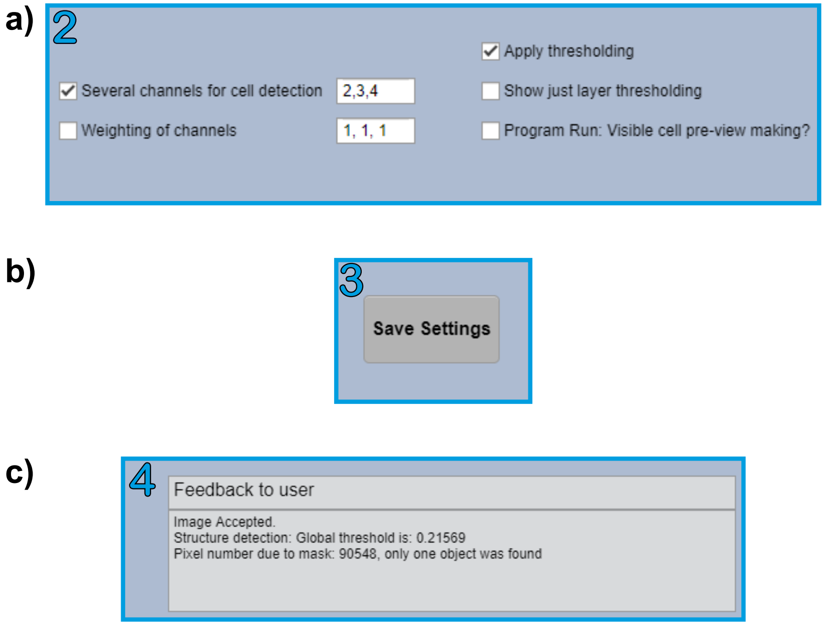

Fig. S23 a) Overview over tab 2 panel 2 b) Overview over tab 2 panel 3 c) Overview over tab 2 panel 4

all channels individually multiplied with the final cell mask as well as the addition of channels multiplied with the final cell mask is made which can be saved. By checking "Comparison to original image" depending on the chosen options for applying threshold (just layer or all), either the smaller stack of selected layers (left) is shown together with the total image stack (right) or the selected layers with the cell detection mask (left) are shown against the selected layers without the cell detection mask (right).

Furthermore, panel 6 offers the saving of cell detection masks and individual channel montages with overlaid cell detection masks ("Save images inclusive mask (latter as tif-stack)".
There are different normalization options. The case "Calculate all normalization possibilities" was removed as it is not needed for the typical use case. One can choose a normalization of the distance between charge centers (field with "1) Normalized by maximal diameter" can be switched to "2) Normalized by averaged diameter" and a normalization of the charge with "1) Charge normalized dependent on shape of cell (bit)" offers all in all 5 options ("2) Charge normalized dependent on protein distribution of cell (bit)", "3) Cell charge normalized corresponding to maximal value of charge multiplied by number of positive charges", "4) Cell charge normalized corresponding to maximal value of charge multiplied by halved number of total charges", ("5) Cell charge normalized corresponding to mean value of charge multiplied by halved number of total charges" was removed as it is not needed for the typical use case.) "6) Own max for each cell (else: dependent on bit)).
Moreover, the intensity of channels can be normalized to increase comparability of cells via checking "Cell average intensity normalized to 1" (average component content the same among big and small cells). "Cell total intensity normalized to 1" (total component content the same among big and small cells) was removed as it is not needed for the typical use case.

H.3.3 Tab 3: Results

Tab 3 shows the results of cell detection and analysis and offers the possibilities to optimize cell detection for single cells and to export analysis data (see Figure S25). Tab 3 can be visually separated in 6 panels:

- Panel 1 which shows the chosen cell layers together with overlaid cell detection mask and offers different possibilities on how to look at the selected cell images
- Panel 2 which shows the result table (in the case of stack-processing of first cell mask generation of all cells and afterwards analysis of all cells a table appears telling whether a cell could be found (1) or not (0).
- Panel 3 for export of analysis with the possibility to export .mat files.
- Panel 4 as feedback field

Panel 1

Panel 1 shows the cell detection mask overlaid on the selected cell layers. Different channels can be chosen to be looked at. You can move through the images by either using "Back" and "Next" button or by grabbing the index and moving it. Options for showing cell images are the same as in tab 2 panel 5: A comparison between original images and cell detection images can be looked at ("Comparison"). If one just wants to look at the selected layers, one needs to check "Show just layer thresholding". For having an overview over channels

a)
b)
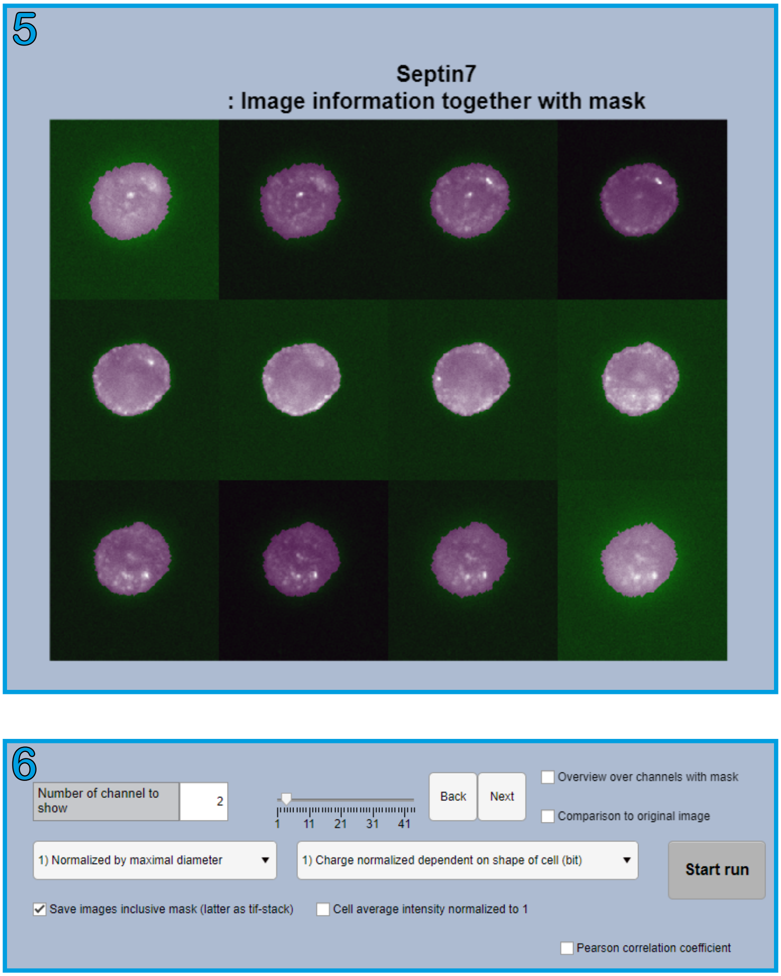

Fig. S24 a) Overview over tab 2 panel 5 and b) tab 2 panel 6

with their cell masks, the condition “Overview over channels with mask” needs to be checked. For getting an idea of which step still needs improvement in cell detection, the option “Program Run: Visible cell pre-view making?” needs to be checked.

Panel 2

Panel 2 contains a part of the results: The absolute value of normalized dipole

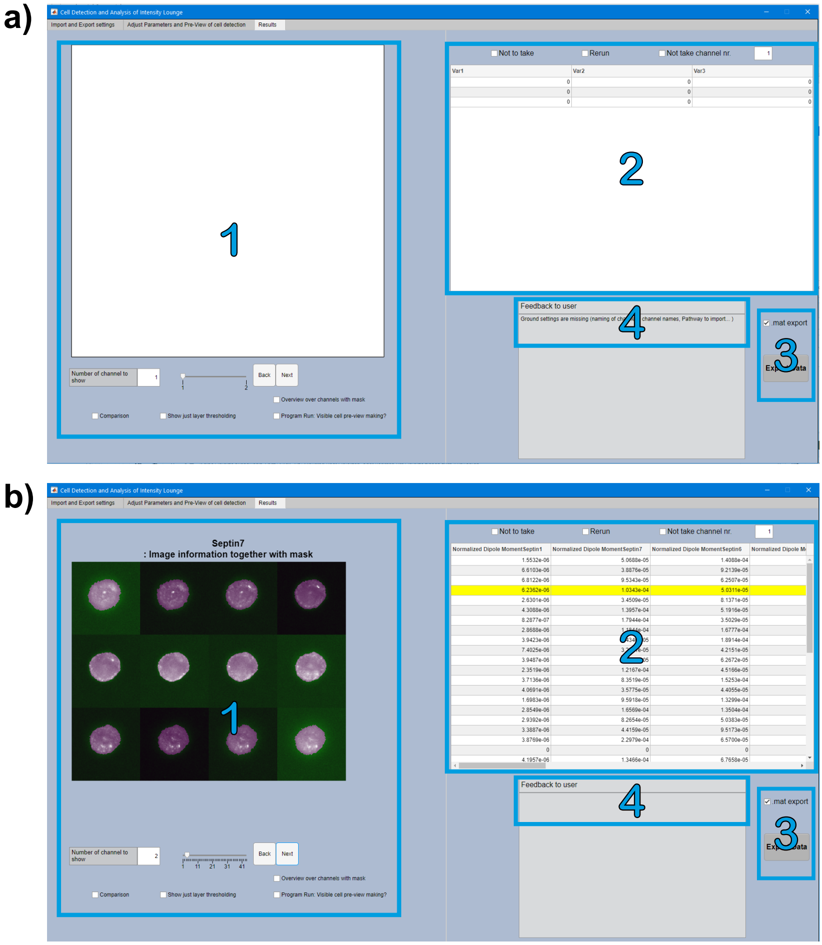

Fig. S25 a) Overview over tab 3 before run-through b) Overview over tab 3 after run-through with analysis directly after cell detection resulting in filled table in panel 2

moment (per channel), the absolute value of normalized distance (per channel), diameter, the normalized charge (per channel) and number of pixels/voxels (per channel). The values for the cell being looked at are marked in yellow. Above the table several possibilities are positioned which help in working with the selected cell. When the cell detection was not good or out of other reasons you want to exclude a cell i.e. not export the data belonging to this cell, you need to check “Not to take” for that cell. The “Not to take” option will always

a)

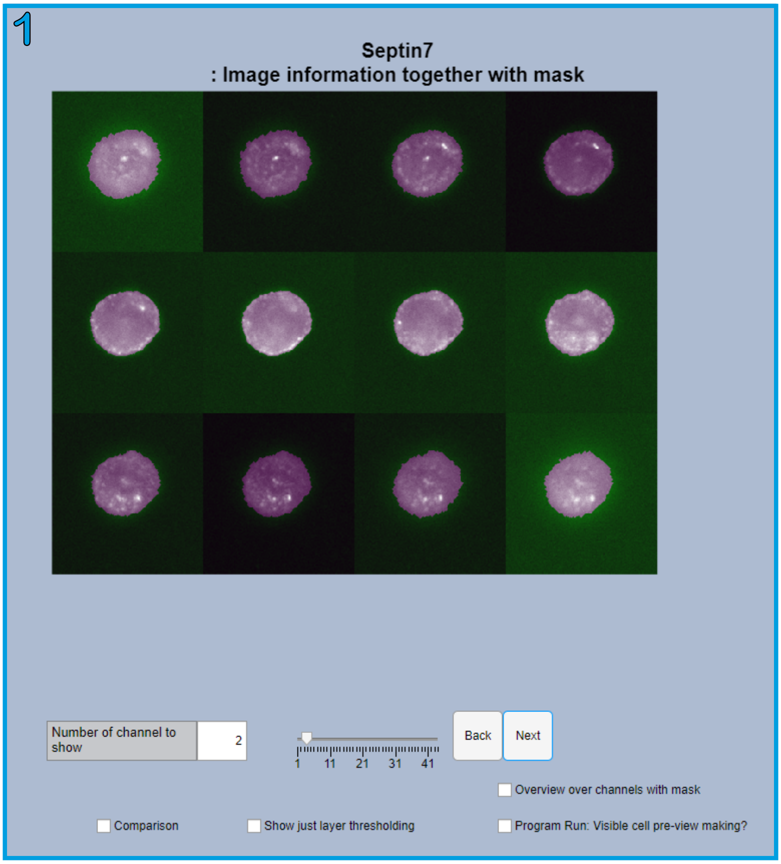

Fig. S26 a) Overview over tab 3 panel 1

be checked when this cell gets looked at and can be taken back by unchecking "Not to take". Only when the export button "Export Data" gets pushed, the value will be excluded from the values being exported.
If the cell detection needs to be improved, the "Rerun" option can be checked. This opens a new, but familiar window (see Figure S29), for which all previously introduced cell detection parameters can be changed for the single cell. Another option is the deletion of single values of a certain channel. This can

be done for the selected cell (in yellow) by writing the number of channel, that shall be deleted, and afterwards checking “Not take channel nr.” . This will write NaN value to the chosen channel, which can’t be undone.

Panel 3

Panel 3 is responsible for the export of analyzed data. The check for “.mat export” allows the additional export of .mat variables which can be worked on e.g. in Matlab. By pressing the “Export Data” button, the export starts. The data is exported as .xls and .txt files to allow further processing of data with other programs. A window shows up asking to give a head name for the exported files. Variables are exported in single variable files and are not combined within a single file. The export happens in the folder chosen as data export pathway.

Panel 4

Panel 4 gives feedback to user, e.g. the time a run-through takes.

H.3.4 Rerun tab

The rerun tab shows up when the user wants to optimize cell detection and checks the “Rerun” option in tab 3 panel 2. Parameters of cell detection (already introduced in tab 2 panel 1) can be changed for a single cell (except for channel/s used for cell detection).

Pressing “Preview of Rerun” just shows a preview of the adapted cell detection result without changing any values. This option can be used to optimize cell detection before starting the recalculation.

The new parameters overwrite the former set ones when “Rerun (table values changed)” is pressed. This starts the recalculation with the modified cell detection mask and changes the table values inclusive all other analysis results.

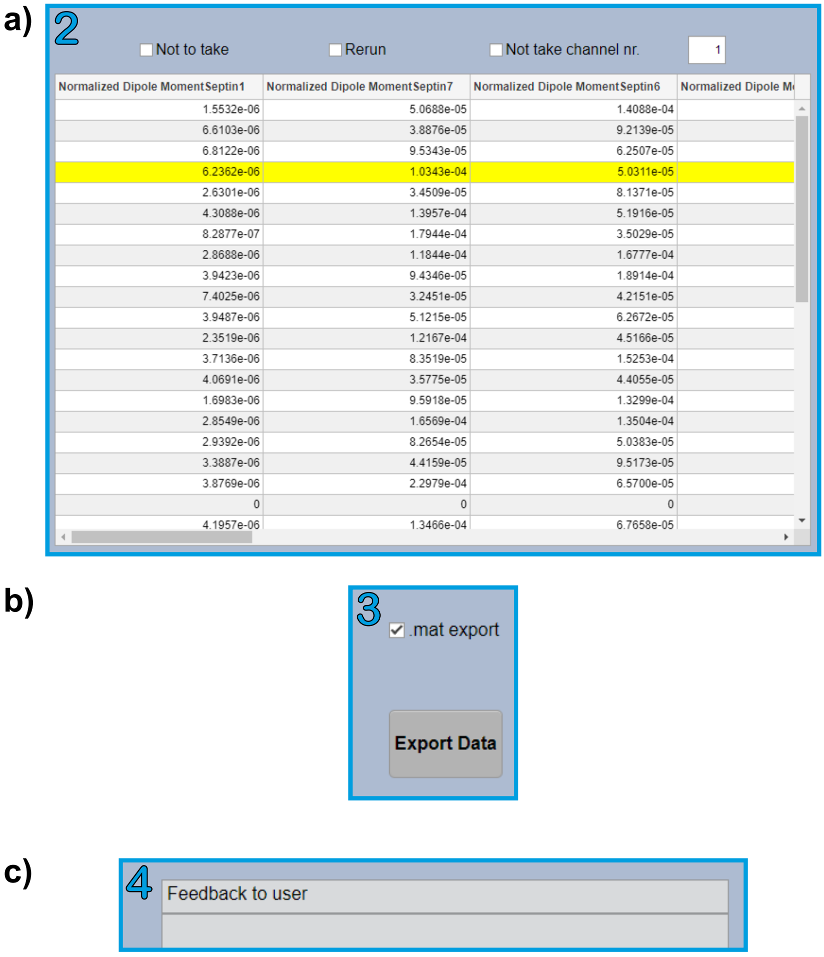

Fig. S27 a) Overview over tab 3 panel 2 b) Overview over tab 3 panel 3 c) Overview over tab 3 panel 4

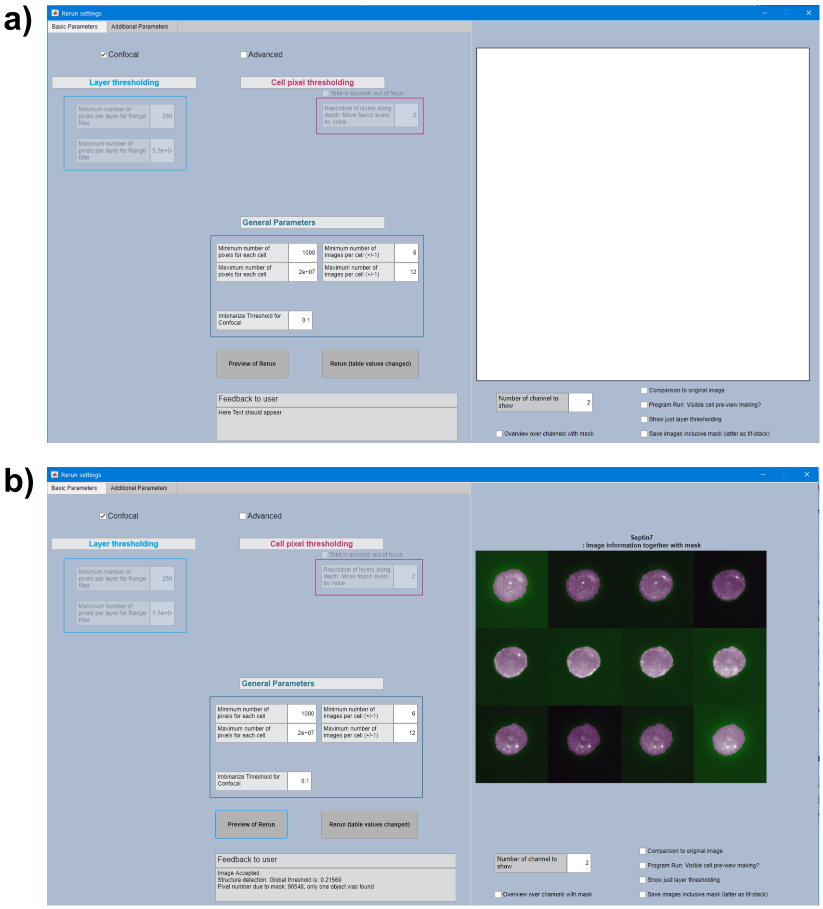

Fig. S28 a) Overview over rerun tab opened after checking "Rerun" in tab 3 panel 2 b) Overview over rerun tab after clicking button "Preview of Rerun"

a)

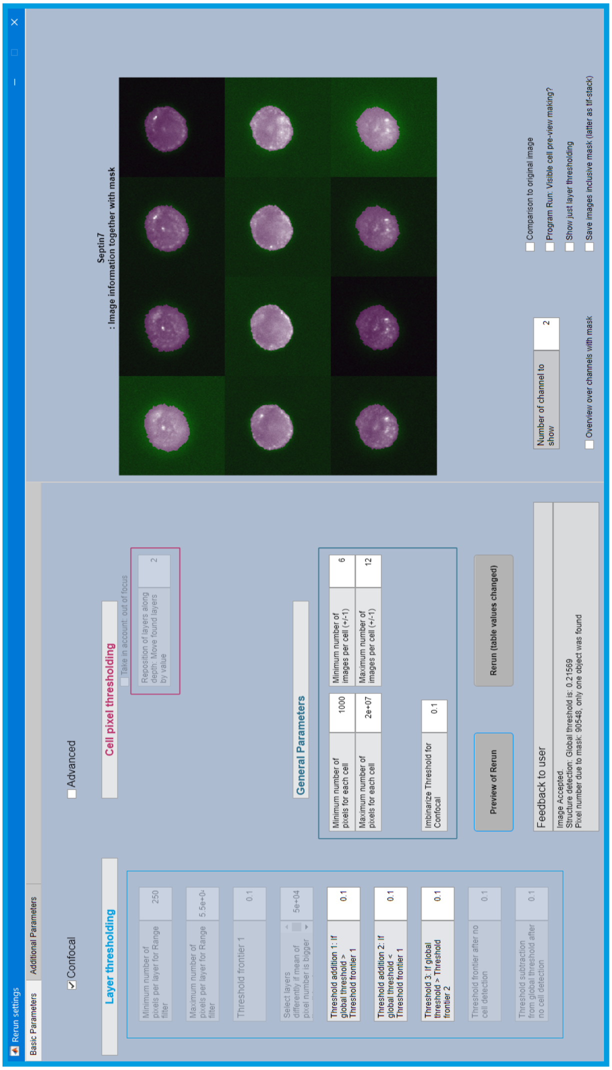

Fig. S29 a) Overview over rerun tab with “Advanced Parameters” setting selected

H.4 Output

H.4.1 Important Parameters for Analysis

The general output of the software consists of the dipole moment vector **P**, averaged and maximal diameter, the number of pixels/voxels related to volume, averaged intensity (adapted when normalization of average intensity to 1 is chosen!), positive charge density, positive pixel/voxel number, total intensity, the vectors between the negative charge weighted center $\mathbf{R}_{-}$ and the middle of cell **M** and the positive charge weighted center $\mathbf{R}_{+}$. Next to it, further parameters are given as output (see Tab. S4).

Table S4 General output

**Name**	**Description**	**Meaning**
DipolMoment<br>dipolmoment.mat	vector of dipole moment **P** with x,y,z<br>(a.u. nm)	possibility to check whether spatial distribution prefers sites at the top or bottom of a cell via z, or whether there are favored directions within a tissue, ...
AvDiameter<br>diameteravvecfinished.mat	averaged cell diameter (nm)	can be used for determining inter-dependence of cell size with other properties
MaxDiameter<br>diametermaxvecfinished.mat	maximal cell diameter (nm)	can be used for determining inter-dependence of cell size with other properties
NumberOfPixels<br>numberofpixels.mat	number of cell pixels/voxels	related to volume of cell, can be multiplied by pixel/voxel volume for getting cell area/ volume
AveragedIntensityAUperPixel<br>avintensity.mat	averaged intensity per pixel/voxel<br>(a.u. / pixel(voxel))<br>adapted if possibilities “Cell average intensity normalized to 1” is checked	average component density including freely diffusing and cluster constructs (for no normalization being chosen)

Name	Description	Meaning
Chargedensitypos qposvec.mat	positive charge density, sum of individual positive charge density (charge divided by pixel/voxel volume, (a.u. / nm^2)/(a.u. / nm^3)	when divided by number of pixels/voxels, the average positive charge density is gained (corresponds to the average value of higher intensity pixel/voxel being above average intensity value and thus gives information about relative average component density at cluster sites), from this value and the number of positive pixels/voxels the averaged original intensity of just positive charge associated pixels/voxels can be calculated; as average intensity differs among cells (different component amount), this can lead to different outcome when comparing average positive intensity (cells can have in reality denser clusters of components, as they have more component content, but relative to average intensity, cells could form more efficiently component clusters; this behavior can be deciphered via averaged positive charge and averaged positive intensity)

Name	Description	Meaning
PositivePixelNumber numberofpixelspos.mat	number of positive charge pixels / voxels number of cell pixels/voxels with intensity values higher than average cell pixel/voxel intensity	related to spatial area/volume of higher intensity pixels/voxels
TotalIntensity PixelIntensitytotal.mat	sum over all cell pixel/voxel intensity values	related to total amount of component within cell, comparison parameter among cells for component amount; average intensity can be calculated via division by NumberOfPixels in the case of chosen normalization method when AveragedIntensityAUperPixel parameter is modified
RnegRmean RnegRmean.mat	$\mathbf{R_{neg}R_{mean}}$, vector between negative charge weighted center $\mathbf{R_{neg}}$ and middle of cell $\mathbf{M}$	distance between negative charge weighted center and middle of cell can be calculated and set into relation to e.g. diameter (e.g. in DAPI channel how cytoplasm is positioned relative to nucleus) OR angle between different negative charge centers (via angle between component 1 $\mathbf{R_{neg}R_{mean}}$ and component 2 $\mathbf{R_{neg}R_{mean}}$) OR similarity between different negative charge centers (via distance between R_{neg} of component 1 and R_{neg} of component 2

Name	Description	Meaning
RposRmean RposRmean.mat	$\mathbf{R_{pos}R_{mean}}$, vector between positive charge weighted center $\mathbf{R_{pos}}$ and middle of cell $\mathbf{M}$	distance between positive charge weighted center and middle of cell can be calculated and set into relation to e.g. diameter (e.g. in protein channel how protein is positioned relative to non-protein areas/volume) OR angle between different positive charge weighted centers (via angle between component 1 $\mathbf{R_{pos}R_{mean}}$ and component 2 $\mathbf{R_{pos}R_{mean}}$; on same side or opposite side of cell) OR similarity between different negative charge centers (via distance between R_{pos} of component 1 and R_{pos} of component 2
RposRneg RposRneg.mat	$\mathbf{R_{pos}R_{neg}}$, vector between positive charge weighted center $\mathbf{R_{pos}}$ and negative charge weighted center $\mathbf{R_{neg}}$	possible to calculate distance between positive and negative charge weighted center before normalization (absolute value of $\mathbf{R_{pos}R_{neg}}$), can look for preference among cells in x,y,z direction (like for dipole moment $\mathbf{P}$ variable)
indexnottaken indexofcellsnottaken.mat	indices of cells that were not taken for final analysis (in third tab of CellDetail: clicked “Not to take”)	parameter which can be used for rerun of analysis on same data set (Data set rerun option 1)
savingofsettingsofcertaincells.mat	cell detection parameters for individual taken cells	parameter which can be used for rerun of analysis on same data set (Data set rerun option 1)

Name	Description	Meaning
names Names.mat	file names taken from data import folder	file names of processed image files, come into order of processing
channelnames allchannelnames.mat	assigned channelnames	assigned names of channels of image files, order of channels
toignore toignore.mat	number/s of channel/s which is/are ignored for analysis	exclusion of analysis of e.g. brightfield channel is possible

Table S5 General output parameters. All normalizations ("Calculate all normalization possibilities" checked)

Name	Description and Meaning
NormalizedChargeOption1<br>qnormalized1.mat	normalized positive charge, normalized by max. possible charge related to half cell size and bit size<br>Influence of cell size and bit size, thus unless normalization option "average to 1" chosen, higher abundance of component leads to higher normalized charge and thus to higher value of absolute value of normalized dipole moment.
NormalizedChargeOption2<br>qnormalized2.mat	normalized positive charge, normalized by max. possible charge related to positive charge pixel/voxel number and bit size<br>Influence of spatial distribution (positive charge pixel/voxel number) and bit size, thus unless normalization option "average to 1" chosen, higher abundance of component leads to higher normalized charge and thus to higher value of absolute value of normalized dipole moment.
NormalizedChargeOption3<br>qnormalized3.mat	normalized positive charge, normalized by max. possible charge related to half cell size and maximal positive charge value per individual cell<br>Influence of cell size and maximal value of positive charge cell pixel/voxel, thus influence of component cluster density distribution among positive charge voxels (positive charge density distribution), homogeneity vs. heterogeneity of clusters (more heterogeneous: lower resulting value, if more homogenous: higher resulting value), important to check positive charge distribution variety among conditions; implemented for avoiding influence of component amount (which can be achieved by optioning for "total to 1" as well) by individual cell-derived positive charge maximum.

**Name**	**Description and Meaning**
NormalizedChargeOption4 qnormalized4.mat	normalized positive charge, normalized by max. possible charge related to positive charge pixel/voxel number and maximal positive charge value per individual cell<br>Influence of spatial distribution (positive charge pixel/voxel number) and maximal value of positive charge cell pixel/voxel, thus influence of component cluster density distribution among positive charge voxels (positive charge density distribution), homogeneity vs. heterogeneity of clusters (more heterogeneous: lower resulting value, if more homogenous: higher resulting value), important to check positive charge distribution variety among conditions; implemented for avoiding influence of component amount (which can be achieved by optioning for "total to 1" as well) by individual cell-derived positive charge maximum.
NormalizedChargeOption5 qnormalized5.mat	normalized positive charge, normalized by max. possible charge related to half cell size and mean positive charge value per individual cell<br>Influence of cell size and mean value of positive charge cell pixel/voxel, thus influence of component cluster density distribution among positive charge voxels (positive charge density distribution), homogeneity vs. heterogeneity of clusters (more heterogeneous: lower resulting value, if more homogenous: higher resulting value), important to check positive charge distribution variety among conditions; implemented for avoiding influence of component amount (which can be achieved by optioning for "total to 1" as well) by individual cell-derived positive charge mean. Mean value in order to avoid influence of high noise pixels/voxels. This option was removed as it is not needed for the typical use case.
**Name**	**Description and Meaning**
NormalizedChargeOption6 qnormalized6.mat	normalized positive charge, normalized by percentage of original intensity of positive charge pixels/voxels relative to total intensity<br>As percentage to input intensity is used, there is no influence by component amount. Even cells with weak signal will get as high values as cells with strong signal as long as the spatial distribution is the same.

**Name**	**Description and Meaning**
NormalizedDipoleMoment11Option dipolmomentnormalized11.mat NormalizedDipoleMomentMaxDiameterChargeOption 1	absolute value of normalized dipole moment, normalized by max. diameter and max. possible charge related to half cell size and bit size; probability distribution of absolute value of normalized dipole moment helps in distinguishing the system to have one or several ground states of polarity, helps in distinguishing spatial distribution changes among different conditions as well as quantifying higher/lower reachable polarity values among different conditions, helps in correlation analysis related to spatial distribution/polarity. Influence of maximal diameter and maximal distance between charge weighted centers and thus influence of cell shape and positioning of charge centers like positioning of charge centers dependent on cell shape with charge centers always/-more often/ at least one sitting at elongated protrusion. In these situations maximal diameter normalization option provides normalization to values between 0 and 1. Influence of cell size and bit size, thus unless normalization option "average to 1" chosen, higher abundance of component leads to higher normalized charge and thus to higher value of absolute value of normalized dipole moment.

Name	Description and Meaning
NormalizedDipoleMoment12Option dipolmomentnormalized12.mat NormalizedDipoleMomentMaxDiameterChargeOption 2	absolute value of normalized dipole moment, normalized by max. diameter and max. possible charge related to positive charge pixel/voxel number and bit size; probability distribution of absolute value of normalized dipole moment helps in distinguishing the system to have one or several ground states of polarity, helps in distinguishing spatial distribution changes among different conditions as well as quantifying higher/lower reachable polarity values among different conditions, helps in correlation analysis related to spatial distribution/polarity. Influence of maximal diameter and maximal distance between charge weighted centers and thus influence of cell shape and positioning of charge centers like positioning of charge centers dependent on cell shape with charge centers always/-more often/ at least one sitting at elongated protrusion. In these situations maximal diameter normalization option provides normalization to values between 0 and 1. Influence of spatial distribution (positive charge pixel/voxel number) and bit size, thus unless normalization option “average to 1” chosen, higher abundance of component leads to higher normalized charge and thus to higher value of absolute value of normalized dipole moment.

Name	Description and Meaning
NormalizedDipoleMoment13Option dipolmomentnormalized13.mat NormalizedDipoleMomentMaxDiameterChargeOption 3	absolute value of normalized dipole moment, normalized by max. diameter and max. possible charge related to half cell size and maximal positive charge value per individual cell; probability distribution of absolute value of normalized dipole moment helps in distinguishing the system to have one or several ground states of polarity, helps in distinguishing spatial distribution changes among different conditions as well as quantifying higher/lower reachable polarity values among different conditions, helps in correlation analysis related to spatial distribution/polarity. Influence of maximal diameter and maximal distance between charge weighted centers and thus influence of cell shape and positioning of charge centers like positioning of charge centers dependent on cell shape with charge centers always/more often/ at least one sitting at elongated protrusion. In these situations maximal diameter normalization option provides normalization to values between 0 and 1. Influence of cell size and maximal value of positive charge cell pixel/voxel, thus influence of component cluster density distribution among positive charge voxels (positive charge density distribution), homogeneity vs. heterogeneity of clusters (more heterogeneous: lower resulting value, if more homogenous: higher resulting value), important to check positive charge distribution variety among conditions; implemented for avoiding influence of component amount (which can be achieved by optioning for “average to 1” as well) by individual cell-derived positive charge maximum.

**Name**	**Description and Meaning**
NormalizedDipoleMoment14Option dipolmomentnormalized14.mat NormalizedDipoleMomentMaxDiameterChargeOption 4	absolute value of normalized dipole moment, normalized by max. diameter and max. possible charge related to positive charge pixel/voxel number and maximal positive charge value per individual cell; probability distribution of absolute value of normalized dipole moment helps in distinguishing the system to have one or several ground states of polarity, helps in distinguishing spatial distribution changes among different conditions as well as quantifying higher/lower reachable polarity values among different conditions, helps in correlation analysis related to spatial distribution/polarity. Influence of maximal diameter and maximal distance between charge weighted centers and thus influence of cell shape and positioning of charge centers like positioning of charge centers dependent on cell shape with charge centers always/more often/at least one sitting at elongated protrusion. In these situations maximal diameter normalization option provides normalization to values between 0 and 1. Influence of spatial distribution (positive charge pixel/voxel number) and maximal value of positive charge cell pixel/voxel, thus influence of component cluster density distribution among positive charge voxels (positive charge density distribution), homogeneity vs. heterogeneity of clusters (more heterogeneous: lower resulting value, if more homogenous: higher resulting value), important to check positive charge distribution variety among conditions; implemented for avoiding influence of component amount (which can be achieved by optioning for "average to 1" as well) by individual cell-derived positive charge maximum.

Name	Description and Meaning
NormalizedDipoleMoment16Option dipolmomentnormalized16.mat NormalizedDipoleMomentMaxDiameterChargeOption 6	absolute value of normalized dipole moment, normalized by max. diameter and max. possible charge related to percentage of original intensity of positive charge pixels/voxels relative to total intensity; probability distribution of absolute value of normalized dipole moment helps in distinguishing the system to have one or several ground states of polarity, helps in distinguishing spatial distribution changes among different conditions as well as quantifying higher/lower reachable polarity values among different conditions, helps in correlation analysis related to spatial distribution/polarity. Influence of maximal diameter and maximal distance between charge weighted centers and thus influence of cell shape and positioning of charge centers like positioning of charge centers dependent on cell shape with charge centers always/-more often/ at least one sitting at elongated protrusion. In these situations maximal diameter normalization option provides normalization to values between 0 and 1. As percentage to input intensity is used, there is no influence by component amount. Even cells with weak signal will get as high values as cells with strong signal as long as the spatial distribution is the same.

Name	Description and Meaning
NormalizedDipoleMoment21Option dipolmomentnormalized21.mat NormalizedDipoleMomentAvDiameterChargeOption 1	absolute value of normalized dipole moment, normalized by averaged diameter and max. possible charge related to half cell size and bit size; probability distribution of absolute value of normalized dipole moment helps in distinguishing the system to have one or several ground states of polarity, helps in distinguishing spatial distribution changes among different conditions as well as quantifying higher/lower reachable polarity values among different conditions, helps in correlation analysis related to spatial distribution/polarity. Influence of averaged diameter and maximal distance between charge weighted centers and thus influence of cell shape and positioning of charge centers: When positioning of charge centers not dependent on cell shape with charge centers not sitting at elongated protrusion: In these situations averaged diameter normalization option can be used to get independent of elongated protrusion/cell shape influence. Influence of cell size and bit size, thus unless normalization option "average to 1" is chosen, higher abundance of component leads to higher normalized charge and thus to higher value of absolute value of normalized dipole moment.

**Name**	**Description and Meaning**
NormalizedDipoleMoment22Option dipolmomentnormalized22.mat NormalizedDipoleMomentAvDiameterChargeOption 2	absolute value of normalized dipole moment, normalized by averaged diameter and max. possible charge related to positive charge pixel/voxel number and bit size; probability distribution of absolute value of normalized dipole moment helps in distinguishing the system to have one or several ground states of polarity, helps in distinguishing spatial distribution changes among different conditions as well as quantifying higher/lower reachable polarity values among different conditions, helps in correlation analysis related to spatial distribution/polarity. Influence of averaged diameter and maximal distance between charge weighted centers and thus influence of cell shape and positioning of charge centers: When positioning of charge centers not dependent on cell shape with charge centers not sitting at elongated protrusion: In these situations averaged diameter normalization option can be used to get independent of elongated protrusion/cell shape influence. Influence of spatial distribution (positive charge pixel/voxel number) and bit size, thus unless normalization option “average to 1” is chosen, higher abundance of component leads to higher normalized charge and thus to higher value of absolute value of normalized dipole moment.

**Name**	**Description and Meaning**
NormalizedDipoleMoment23Option dipolmomentnormalized23.mat NormalizedDipoleMomentAvDiameterChargeOption 3	absolute value of normalized dipole moment, normalized by averaged diameter and max. possible charge related to half cell size and maximal positive charge value per individual cell; probability distribution of absolute value of normalized dipole moment helps in distinguishing the system to have one or several ground states of polarity, helps in distinguishing spatial distribution changes among different conditions as well as quantifying higher/lower reachable polarity values among different conditions, helps in correlation analysis related to spatial distribution/polarity. Influence of averaged diameter and maximal distance between charge weighted centers and thus influence of cell shape and positioning of charge centers: When positioning of charge centers not dependent on cell shape with charge centers not sitting at elongated protrusion: In these situations averaged diameter normalization option can be used to get independent of elongated protrusion/cell shape influence. Influence of cell size and maximal value of positive charge cell pixel/voxel, thus influence of component cluster density distribution among positive charge voxels (positive charge density distribution), homogeneity vs. heterogeneity of clusters (more heterogeneous: lower resulting value, if more homogenous: higher resulting value), important to check positive charge distribution variety among conditions; implemented for avoiding influence of component amount (which can be achieved by optioning for “average to 1” as well) by individual cell-derived positive charge maximum.

Name	Description and Meaning
NormalizedDipoleMoment24Option dipolmomentnormalized24.mat NormalizedDipoleMomentAvDiameterChargeOption 4	absolute value of normalized dipole moment, normalized by averaged diameter and max. possible charge related to positive charge pixel/voxel number and maximal positive charge value per individual cell; probability distribution of absolute value of normalized dipole moment helps in distinguishing the system to have one or several ground states of polarity, helps in distinguishing spatial distribution changes among different conditions as well as quantifying higher/lower reachable polarity values among different conditions, helps in correlation analysis related to spatial distribution/polarity. Influence of averaged diameter and maximal distance between charge weighted centers and thus influence of cell shape and positioning of charge centers: When positioning of charge centers not dependent on cell shape with charge centers not sitting at elongated protrusion: In these situations averaged diameter normalization option can be used to get independent of elongated protrusion/cell shape influence. Influence of spatial distribution (positive charge pixel/voxel number) and maximal value of positive charge cell pixel/voxel, thus influence of component cluster density distribution among positive charge voxels (positive charge density distribution), homogeneity vs. heterogeneity of clusters (more heterogeneous: lower resulting value, if more homogenous: higher resulting value), important to check positive charge distribution variety among conditions; implemented for avoiding influence of component amount (which can be achieved by optioning for “average to 1” as well) by individual cell-derived positive charge maximum.

**Name**	**Description and Meaning**
NormalizedDipoleMoment26Option dipolmomentnormalized26.mat NormalizedDipoleMomentAvDiameterChargeOption 6	absolute value of normalized dipole moment, normalized by averaged diameter and max. possible charge related to percentage of original intensity of positive charge pixels/voxels relative to total intensity; probability distribution of absolute value of normalized dipole moment helps in distinguishing the system to have one or several ground states of polarity, helps in distinguishing spatial distribution changes among different conditions as well as quantifying higher/lower reachable polarity values among different conditions, helps in correlation analysis related to spatial distribution/polarity. Influence of averaged diameter and maximal distance between charge weighted centers and thus influence of cell shape and positioning of charge centers: When positioning of charge centers not dependent on cell shape with charge centers not sitting at elongated protrusion: In these situations averaged diameter normalization option can be used to get independent of elongated protrusion/cell shape influence. As percentage to input intensity is used, there is no influence by component amount. Even cells with weak signal will get as high values as cells with strong signal as long as the spatial distribution is the same.

Name	Description and Meaning
NormalizedDistanceAvDiameter Rnormalized_finished2.mat	normalized distance between positive and negative charge center ($\mathbf{R_{pos}}$, $\mathbf{R_{neg}}$), normalized by averaged diameter of cell. Influence of averaged diameter and distance between charge weighted centers and thus influence of cell shape and positioning of charge centers: When positioning of charge centers not dependent on cell shape with charge centers not sitting at elongated protrusion: In these situations averaged diameter normalization option can be used to get independent of elongated protrusion/cell shape influence.
NormalizedDistanceMaxDiameter Rnormalized_finished1.mat	normalized distance between positive and negative charge center ($\mathbf{R_{pos}}$, $\mathbf{R_{neg}}$), normalized by max. diameter of cell. Influence of maximal diameter and maximal distance between charge weighted centers and thus influence of cell shape and positioning of charge centers like positioning of charge centers dependent on cell shape with charge centers always/more often/ at least one sitting at elongated protrusion. In these situations maximal diameter normalization option provides normalization to values between 0 and 1.

**Name**	**Description and Meaning**
Pearsoncorrelationcoefficient<br>Pearsoncorrelationcoefficient.mat	Pearson correlation coefficient = $\frac{\sum_i (x_i - <x>) \cdot (y_i - <y>)}{(\sum_i (x_i - <x>)^2 \cdot \sum_i (y_i - <y>)^2)^{0.5}}$, with <x> and <y> being the mean value of pixel/voxel intensities of a channel, measure of linear correlation ranging from -1 to 1 with 1/-1 describing a linear relationship between x and y (in this case: channel intensity values), for the outcome 1 positive linear correlation, for outcome -1 decreasing regression slope of linear correlation (y decreasing with increasing x), for outcome 0 no linear dependency

Name	Description and Meaning
displacementDipoleMomentfromMiddle displacementdipolfromM.mat	if distribution of component within cell more confined in one half, detectable; for apolar: random values possible; displacementDipoleMomentfromMiddle.mat, (number of images, number of channels)
displacementRposRnegMRposMRneg displacementRposRnegtoMorthogonal.mat	if cell distribution like one half neutral, other half positive and negative charge: detectable; thus, whether positive and negative charge center are directly opposite (which would be more natural for a concentration increase/decrease) or whether more constricted gradient in cell space; for apolar: random values possible, displacement $\delta = \sin(angle(\overrightarrow{\mathbf{MR}_+}\overrightarrow{\mathbf{R}_+\mathbf{R}_-})$, displacementRposRnegMRposMRneg.mat (number of images, number of channels)

1

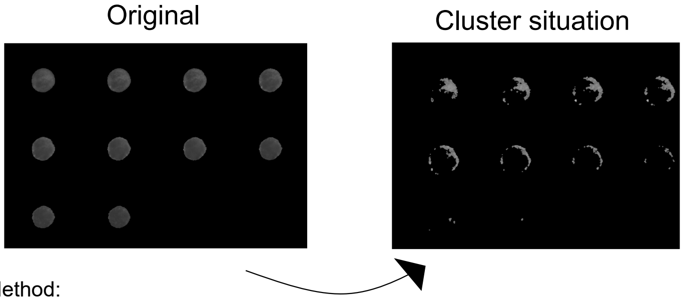

Cluster Method:
voxels above av. value of pos. charge voxels
morphological opening (erasion + dilation)
only clusters with more than 50 pixels/voxels taken

parameters:

meanintensity (mean intensity per single cluster)
maxintensity (max intensity per single cluster)
volumelist (number of pixels/voxels per cluster)
voxellistlist (only as .mat file, indices of pixels/voxels belonging to single clusters)
voxelvalueslist (only as .mat file, values of pixels/voxels belonging to single clusters)

centroid position (bary center position of each cluster (x,y,z) in pixel/voxel number)
weightedcentroid (intensity weighted bary center position of each cluster (x,y,z) in pixel/voxel number)

equiv. diameter (diameter in pixel/voxel number, caution if not isotropic pixel/voxel size!)
surfacearea (surface area of each cluster in pixel/voxel, caution if not isotropic pixel/voxel size!)

arrayfixed4distancelist (array of ranked distance of single cluster to strongest cluster (by angle of bary center (just x,y direction)), <= 45°: 1.column, 45 < x <= 90°: 2.column, 90° < x <= 135°: 3.column, 135° < x <= 180°: 4.column, number of clusters of ranks given)
arrayfixed5strengthlist (array of ranked strength of single cluster (by cluster percent), >= 0.6: 1.column, 0.4 <= x < 0.6: 2.column, 0.2 <= x < 0.4: 3.column, 0.1 <= x < 0.2: 4.column, 0<= x < 0.1: 5.column, number of clusters of ranks given)
sorted_array_tofilldistancelist (strength cluster ranks, sorted after descending from biggest/strongest cluster; same order of cluster indices as sorted_array_tofillstrengthlist)
sorted_array_tofillstrengthlist (distance cluster ranks, sorted after descending from biggest/strongest cluster; same order of cluster indices as sorted_array_tofilldistancelist)
distancestrengthlist (combination of distance and strength rank information; sorted_array_tofilldistancelist separated from sorted_array_tofillstrengthlist by 0, ends with 0; information whether bigger cluster on one side / opposite sides, whether mainly same size, ...)

Fig. S30 Cluster methods ("All parameters taken" checked). 1) Clustering by neighbor pixels/voxels of positive charge pixels/voxels above average value of positive charge pixels/voxels.

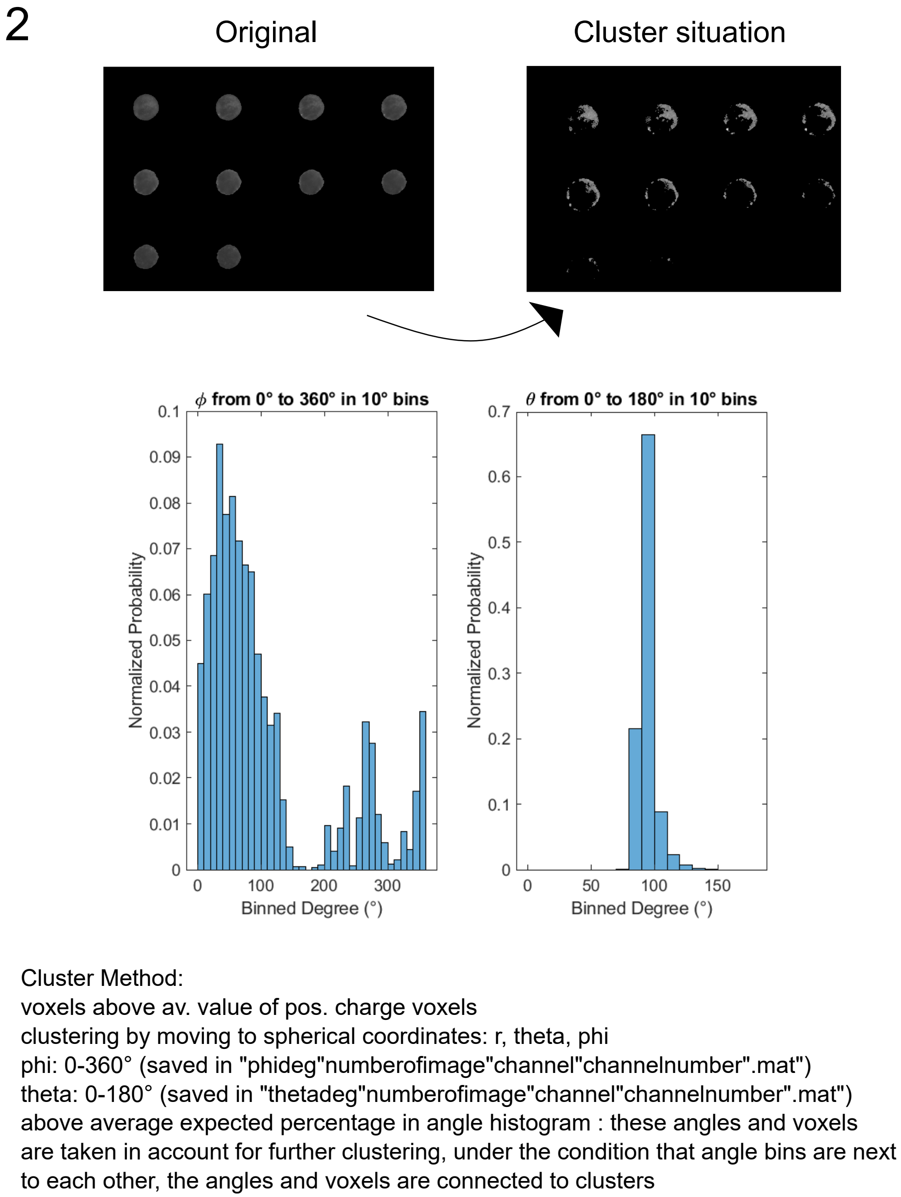

Fig. S31 Cluster methods (”All parameters taken” checked). 2) Clustering by transfer of positive charge pixels/voxels above average value of positive charge pixels/voxels to spherical coordinates and histogram angles. Clustering of pixels/voxels according to angle bins above random percentage value.

2

parameters:

general cluster information

countessphi/theta (number of bins above expected random percentage value)
percoverphi/theta (percentage of bins above expected random percentage value)
howmuchoveritatallphi/theta (total above expected random percentage of connected bins above expected random percentage value)
distancetoavvalphi/theta (sum of above and below random value of histogram, distance to complete random pixel/voxel distribution)
histogramvaluesphi/theta (angle histogram probability bin values)
clusterallphi/theta (indices of pixels/voxels belonging to clustered bins, per cluster)
clusterlengthphi/theta (number of pixels/voxels per clustered bins)
allclusterinputphi/theta (sum of indices of pixels/voxels belonging to clustered bins),
clusterpercentphi/theta (percentage of individual cluster to whole cluster amount),
clusterpercenttoallpixelsposphi/theta (percentage of individual cluster to whole voxels above av. value of pos. charge voxels amount)

calculation of distance and strength parameters

startpointsphi/theta (starting bin number of clustered bins, per cluster)
meanpointsphi/theta (middle bin number of clustered bins, between starting and end bin number, per cluster)
endpointsphi/theta (end bin number of clustered bins, per cluster)
lengthpointsphi/theta (number of clustered bins, per cluster)
distancetonextphi/theta (number of bins between clustered bins)
distancetomeanphi/theta (number of bins between mean value of clustered bins)

distance and strength information

arrayofclustersdistancephi/theta (array of ranked distance of cluster to strongest cluster (by distance between mean position), <= 45°: 1.column, 45 < x <= 90°: 2.column, 90°< x <= 135°:3.column, 135°< x <=180°:4.column, number of clusters of ranks given)
arrayofclustersphi/theta (1.column: clusterpercent, 2.column: mean of strongest cluster position to mean of individual cluster position (relative) 3.column : 360°-mean of strongest cluster position - mean of individual cluster position (global)
arrayofclusterstrengthphi/theta (array of ranked strength of cluster (by cluster percent), >=0.6: 1.column , 0.4 <= x < 0.6: 2.column, 0.2 <= x < 0.4: 3.column, 0.1 <= x < 0.2: 4.column, 0 <= x < 0.1: 5.column, number of clusters of ranks given)

Fig. S32 Cluster methods ("All parameters taken" checked). 2) Clustering by transfer of positive charge pixels/voxels above average value of positive charge pixels/voxels to spherical coordinates and histogram angles. Clustering of pixels/voxels according to angle bins above random percentage value.

Table S6 Files found in image folder after run-through

**Name**	**Description and Meaning**
image Cell mask	detected mask of cell, as .tif [name of image 'Masked Cell' number of image in analysis '.tif']
channel image multiplied with mask	[name of image, name of channel '.png']

Table S7 Files found in image folder after run-through for all parameters

**Name**	**Description and Meaning**
histogram images	histogram of ϕ and θ in clustering option based on transfer to spherical coordinates, [name of image, name of channel, 'phiand-theta.png']
image of qpos	image of positive charge voxels in cell mask, per channel, [name of image, name of channel, 'qpos.png']
image of qposofqpos	image of charge voxels above mean intensity of positive charge voxels in cell mask, per channel, [name of image, name of channel, 'qposofqpos.png']
image of qposofqposafteropening	image of charge voxels above mean intensity of positive charge voxels in cell mask, per channel, after erasion and dilation operation to get rid of single high intensity voxels, [name of image, name of channel, 'qposofqposafteropening.png'
list_neg	matrix with columns of x_-, y_- and z_- (positions of negative charge pixels/voxels), and negative charge density of individual pixel/voxel per nm^2/nm^3 χ_- and i_- (intensity value after background intensity subtraction, for every negative charge pixel/voxel); saved individually for each cell and channel singly, under image folder pathway, ['list_neg cell' cell number channel name]
list_pos	matrix with columns of x_+, y_+ and z_+ (positions of positive charge pixels/voxels), and positive charge density of individual pixel/voxel per nm^2/nm^3 χ_+ and i_+ (intensity value after background intensity subtraction, for every positive charge pixel/voxel); saved individually for each cell and channel singly, under image folder pathway, ['list_pos cell' image number channel name]

**Name**	**Description and Meaning**
Mmean pix	position of middle point of cell volume in pixels/voxels, saved under image folder pathway, [Mmean pix cell' image number channel name]
R_neg	position of negative charge weighted mean position of cell in pixels/voxels, saved under image folder pathway, ['R_neg cell' image number channel name]
R_pos	position of positive charge weighted mean position of cell in pixels/voxels, saved under image folder pathway, ['R_pos cell' image number channel name]

Appendix I Distribution function of absolute value of normalized dipole moment $\boldsymbol{P_n}$

I.1 Rayleigh formula

Rayleigh distribution can be used for the probability distribution description of vector magnitude of a 2D vector whose components are uncorrelated, normally distributed with equal variance and zero mean, e.g. for wind velocity in x- and y-direction,.

As we have a 3D vector of the dipole moment **P** for which we have uncorrelated, normally distributed x,y,z values with equal variance and zero mean, we need to extend the Rayleigh distribution by a third component to get a description of the probability distribution of the absolute value of normalized dipole moment P_n. Then the formulae for the corresponding expectation value (mean) and variance for P_n can be found.

I.2 Derivation of Rayleigh distribution

The probability density function of the Rayleigh distribution in 1D case for variable x is given by

$$f(x;\ \sigma) = \frac{x}{\sigma^2} e^{-x^2/(2\sigma^2)}, x \geq 0 \tag{I8}$$

with σ being the scale parameter of the distribution. The cumulative distribution function (CDF) for x $\in$ [0, ∞) is the following:

$$F(x;\ \sigma) = 1 - e^{-x^2/(2\sigma^2)} \tag{I9}$$

The mean of a Rayleigh distributed variable is

$$\mu(X) = \sigma\sqrt{\frac{\pi}{2}} \tag{I10}$$

and the variance

$$Var(X) = (2 - \frac{\pi}{2})\sigma^2 \tag{I11}$$

The vector magnitude of a 2D vector is Rayleigh distributed if the vector magnitude is calculated as $R = \sqrt{X^2 + Y^2}$ where $X \approx N(0, \sigma^2)$ and $Y \approx N(0, \sigma^2)$ are independent normal random variables with same variance ($N(0, \sigma^2)$: normal distribution with zero mean and variance σ^2).

Derivation

The probability density of the normal distribution is:

$$f(x) = \frac{1}{\sqrt{2\pi}\sigma} e^{-x^2/(2\sigma^2)} \tag{I12}$$

The probability density of two directions (x and y) is a multiplication:

$$f_{X,Y} = \frac{1}{2\pi\sigma^2} e^{-(x^2+y^2)/(2\sigma^2)} \tag{I13}$$

For the magnitude of a 2D vector ($K := \sqrt{X^2+Y^2}$) the cumulative distribution function can be calculated by:

$$F_K(k) = P|\sqrt{X^2+Y^2} \leq k| = \int_{x^2+y^2 \leq k^2} \int \frac{1}{2\pi\sigma^2} e^{-(x^2+y^2)/(2\sigma^2)} dxdy \tag{I14}$$

with k being the maximal absolute value K can take. As a next step we transform to polar coordinates: $r := \sqrt{x^2+y^2}$ and $\phi := \arctan(\frac{y}{x})$, so that $x = r\cos(\phi)$ and $y = r\sin(\phi)$. Thus, one obtains:

$$F_K(k) = \int_{-\infty}^{k} \int_{-\infty}^{\sqrt{k^2-y^2}} f_{X,Y}(x,y) dxdy = \tag{I15}$$

$$\int_{-\infty}^{k} \int_{-\infty}^{\sqrt{k^2-y^2}} \frac{1}{2\pi\sigma^2} e^{-(x^2+y^2)/(2\sigma^2)} dxdy = \tag{I16}$$

$$\int_{0}^{2\pi} \int_{0}^{k} \frac{1}{2\pi\sigma^2} e^{-(r^2\cos(\phi)^2 + r^2\sin(\phi)^2)/(2\sigma^2)} rdrd\phi = \tag{I17}$$

$$\int_{0}^{2\pi} \int_{0}^{k} \frac{1}{2\pi\sigma^2} e^{-r^2/(2\sigma^2)} rdrd\phi = \tag{I18}$$

$$\frac{1}{2\pi\sigma^2} \int_{0}^{2\pi} \int_{0}^{k} e^{-r^2/(2\sigma^2)} rdr = \tag{I19}$$

$$\frac{1}{\sigma^2} \int_{0}^{k} re^{-r^2/(2\sigma^2)} dr \tag{I20}$$

The probability density of F_K is thus the function:

$$f_K(k) = \frac{k}{\sigma^2} e^{-k^2/(2\sigma^2)} \tag{I21}$$

For the expectation value, the integral

$$E(k) = \int_{0}^{\infty} k \frac{k}{\sigma^2} e^{-k^2/(2\sigma^2)} \tag{I22}$$

is solved to get:

$$E(k) = \sigma\sqrt{\frac{\pi}{2}} \tag{I23}$$

For the variance value, the integral

$$Var(k) = \int_{-\infty}^{\infty} (E(k) - k)^2 f_K(k) dk \tag{I24}$$

is solved to get:

$$Var(k) = -\frac{2\pi|\sigma|^3}{\sigma} \tag{I25}$$

For variance in realistic case, the variance value needs to be solved for an integral ranging from 0 to infinity:

$$Var(k) = \int_{0}^{\infty} (E(k) - k)^2 f_K(k) dk = \frac{1}{2}\sigma^2 \cdot (-\frac{2 \cdot \pi}{sgn(\sigma^3)} + \pi + 4) = \sigma^2 \cdot (2 - \frac{\pi}{2}) \tag{I26}$$

I.3 Extended Derivation in 3D case

The probability density of three directions (x, y and z) for normally distributed components of a vector can be described as:

$$f_{X,Y,Z} = \frac{1}{(2\pi)^{\frac{3}{2}}\sigma^3} e^{-(x^2+y^2+z^2)/(2\sigma^2)} \tag{I27}$$

We define the magnitude of the 3D vector as $K := \sqrt{X^2 + Y^2 + Z^2}$ and the cumulative distribution function can be calculated by $F_K(k) = P|\sqrt{X^2 + Y^2 + Z^2} \leq k|$:

$$F_K(k) = \int_{x^2+y^2+z^2 \leq k^2} \int\int \frac{1}{(2\pi)^{\frac{3}{2}}\sigma^3} e^{-(x^2+y^2+z^2)/(2\sigma^2)} dxdydz = \tag{I28}$$

$$\int_{-\infty}^{k} \int_{-\infty}^{\sqrt{k^2-z^2}} \int_{-\infty}^{\sqrt{k^2-z^2-y^2}} \frac{1}{(2\pi)^{\frac{3}{2}}\sigma^3} e^{-(x^2+y^2+z^2)/(2\sigma^2)} dxdydz \tag{I29}$$

We now make use of spherical coordinates:

$$x = r\sin(\theta)\cos(\phi) \tag{I30}$$

$$y = r\sin(\theta)\sin(\phi) \tag{I31}$$

$$z = r\cos(\theta) \tag{I32}$$

with $r \in [0,k], \theta \in [0,\pi], \phi \in [0,2\pi]$. The equation of the cumulative distribution function can thus be written as:

$$F_K(k) = \int_0^k \int_0^\pi \int_0^{2\pi} \frac{1}{(2\pi)^{3/2}\sigma^3} \tag{I33}$$

$$e^{-(r^2\sin(\theta)^2\cos(\phi)^2+r^2\sin(\theta)^2\sin(\phi)^2+r^2\cos(\theta)^2)/(2\sigma^2)} r^2\sin(\theta)drd\theta d\phi \tag{I34}$$

$$=^{\sin(\alpha)^2+\cos(\alpha)^2=1} \frac{1}{(2\pi)^{3/2}\sigma^3} \int_0^k \int_0^\pi \int_0^{2\pi} e^{-r^2/(2\sigma^2)} r^2 \sin(\theta)drd\theta d\phi = \tag{I35}$$

$$\frac{1}{(2\pi)^{3/2}\sigma^3} |-\cos(\theta)|_0^\pi \cdot |\phi|_0^{2\pi} \int_0^k e^{-r^2/(2\sigma^2)} r^2 dr = \tag{I36}$$

$$\frac{1}{(2\pi)^{3/2}\sigma^3} \cdot 2 \cdot 2\pi \int_0^k e^{-r^2/(2\sigma^2)} r^2 dr = \tag{I37}$$

$$\frac{\sqrt{\frac{2}{\pi}}}{\sigma^3} \int_0^k e^{-r^2/(2\sigma^2)} r^2 dr = \tag{I38}$$

$$\frac{\sqrt{\frac{2}{\pi}}(\sqrt{\frac{\pi}{2}}\sigma^3 erf(\frac{k}{\sqrt{2}\sigma}) - k\sigma^2 e^{\frac{-k^2}{2\sigma^2}})}{\sigma^3} \tag{I39}$$

Thus, the probability density function for the 3-dimensional case is

$$f_K(k) = \sqrt{\frac{2}{\pi}} \frac{k^2}{\sigma^3} e^{-k^2/(2\sigma^2)} \tag{I40}$$

and the expectation value can be calculated by:

$$E(k) = \int_0^\infty k \cdot \sqrt{\frac{2}{\pi}} \frac{k^2}{\sigma^3} e^{-k^2/(2\sigma^2)} dk = 2\sqrt{\frac{2}{\pi}}\sigma \tag{I41}$$

The variance can be calculated by:

$$Var(k) = \int_0^{+\infty} \frac{(2\sigma\sqrt{\frac{2}{\pi}} - k)^2 k^2 \sqrt{\frac{2}{\pi}} e^{-\frac{k^2}{2\sigma^2}}}{\sigma^3} dk = \tag{I42}$$

$$\frac{\sigma((8+3\pi)|\sigma| - 16\sigma)}{\pi} = \tag{I43}$$

$$\sigma^2 \cdot (3 - \frac{8}{\pi}) \tag{I44}$$

Hence, by fitting the probability density function for the 3-dimensional case on the P_n distribution or by calculating the cumulative distribution function for the P_n distribution and fitting, one can gain σ and calculate the expectation value and variance according to the above formulae.

Appendix J Validation of code - scenarios

J.1 Comparison: Analytical result vs. Algorithm result

J.1.1 Description

A sphere of 5000 nm radius is implemented with voxel size 110x110x750 nm^3 resulting via calculation (see Fig. S33 for visual aspect and equations J45 , J46, J47) in a mask of a sphere of 13 layers (see Fig. S34). The parameter t is the number of steps from middle plane in z direction, so for example for $t = 1$: $r_t = r_1$..

$$\sin(\alpha) = \frac{r_t}{r_0} \tag{J45}$$

$$\cos(\alpha) = \frac{t \cdot \Delta z}{r_0} \tag{J46}$$

$$r_t = r_0 \cdot \sin(\arccos\left(\frac{t \cdot \Delta z}{r_0}\right)) \tag{J47}$$

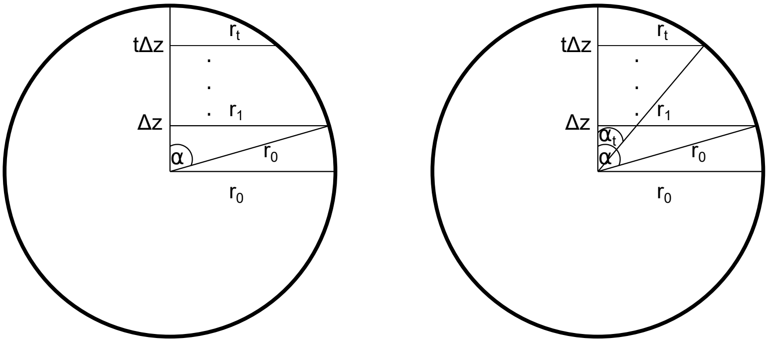

Fig. S33 Planar xz view for gaining radius r_t per layer. On the left handside, the scheme is shown for gaining r_1, while the right handside shows the scheme for arbitrary r_t.

The translation of radius into voxels yields not exactly the same diameter as analytically suggested, as we move from metric/ratio scale to ordinal scale.

J.1.2 Comparison

Essential parameters for comparison of analytical and algorithm solution are:

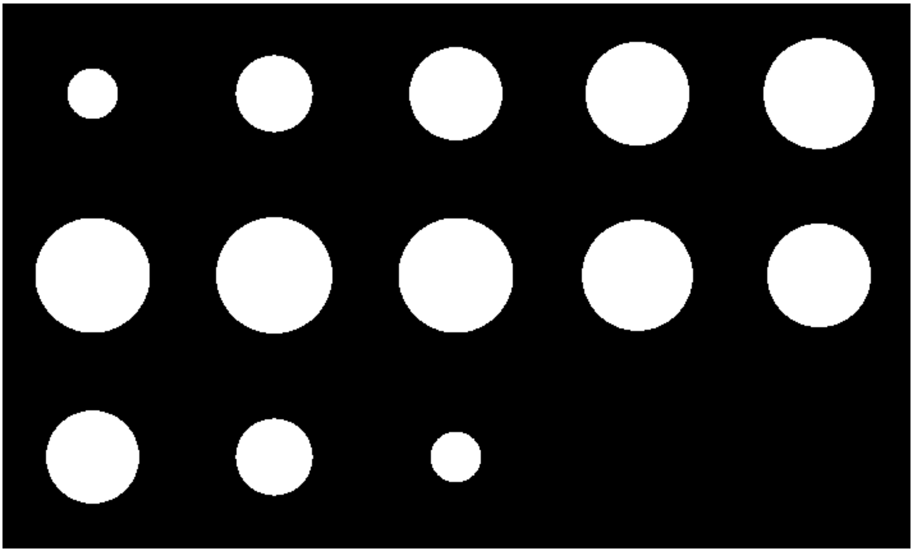

Fig. S34 Mask of sphere. Diameter chosen: 10,000 nm, voxel size chosen: width: 110 nm, height: 110 nm, depth: 750 nm

- q_+: summed positive charge density; $q_+ = \sum_{i=1}^{n} \frac{Q_{+,i}}{voxel\ volume}$ with $Q_{+,i}$ being the positive charge of an individual voxel and n the number of positive charge voxels
- q_n: normalized charge, $q_n = \frac{q_+}{normalization\ option}$ (normalization options: maximal possible value by bit size, dependent on shape of cell (q_{n1}) or protein distribution (q_{n2}); dependent on maximal value of charge density, dependent on protein distribution (q_{n3}) or shape of cell (q_{n4}); dependent on mean value of charge density to avoid influence of bright noise signal and dependent on shape of cell (q_{n5}), was removed in the final software as it is not needed for the typical use case; dependent on total intensity at beginning and related value of in the end detected positive charge (q_{n6}) with no influence of shape of cell. q_{n6} is not practical in these examples, as starting intensity values are for convenience selected in a way to give the value 0 or a value near 0. For exact value of 0, Infinity is the result, for a value near 0 high numbers are the result.
- $|\mathbf{MR_{neg}}|$: Distance between middle of cell and negative charge-weighted center
- $|\mathbf{MR_{pos}}|$: Distance between middle of cell and positive charge-weighted center
- $|\mathbf{R_{pos}R_{neg}}|$: Distance between positive and negative charge-weighted centers.
- R_n: absolute value of normalized distance between $\mathbf{R_{pos}}$ and $\mathbf{R_{neg}}$; $R_n = \frac{|\mathbf{R_{pos}R_{neg}}|}{normalizationoption}$ (normalization options: maximal diameter, averaged diameter)

- P_n: absolute value of normalized dipole moment vector (dipole moment vector: $\mathbf{P}$), resulting from multiplication of q_n and R_n (normalization options are all possible combinations of q_n and R_n resulting in 12 options)
- n_{pos}: number of positive charge voxels

General parameters that do not change and are fixed with mask:

- diameter (due to change from metric scale to ordinal scale offset from 10,000 nm: maximal diameter is 9999.7 nm and averaged diameter 9915.3 nm; these numbers are not used for calculation of analytical solution, thus R_{n2} will show deviation in some cases as too far away from 10,000 nm, while R_{n1} still in good agreement as little deviation from 10,000 nm.) At some point, even R_{n1} deviation from exact value due to offset of maximal diameter from 10,000 nm will show later deviations in downstream parameters.
- number of voxels (in this case: 57801)
- **M**: middle of mask position, ideally in voxels M(71/71/7), however for program **M**(7755/7755/4875) due to program resorting to subtracting half of voxel length to place points ideally in the center of voxel. The same subtraction happens for $\mathbf{R_{pos}}$ and $\mathbf{R_{neg}}$

We look at the charge scenarios visible in Fig. S35, S36, S37. They all relate to the same mask of sphere (visible in Fig. S34), but are filled with different charge distributions and strengths.

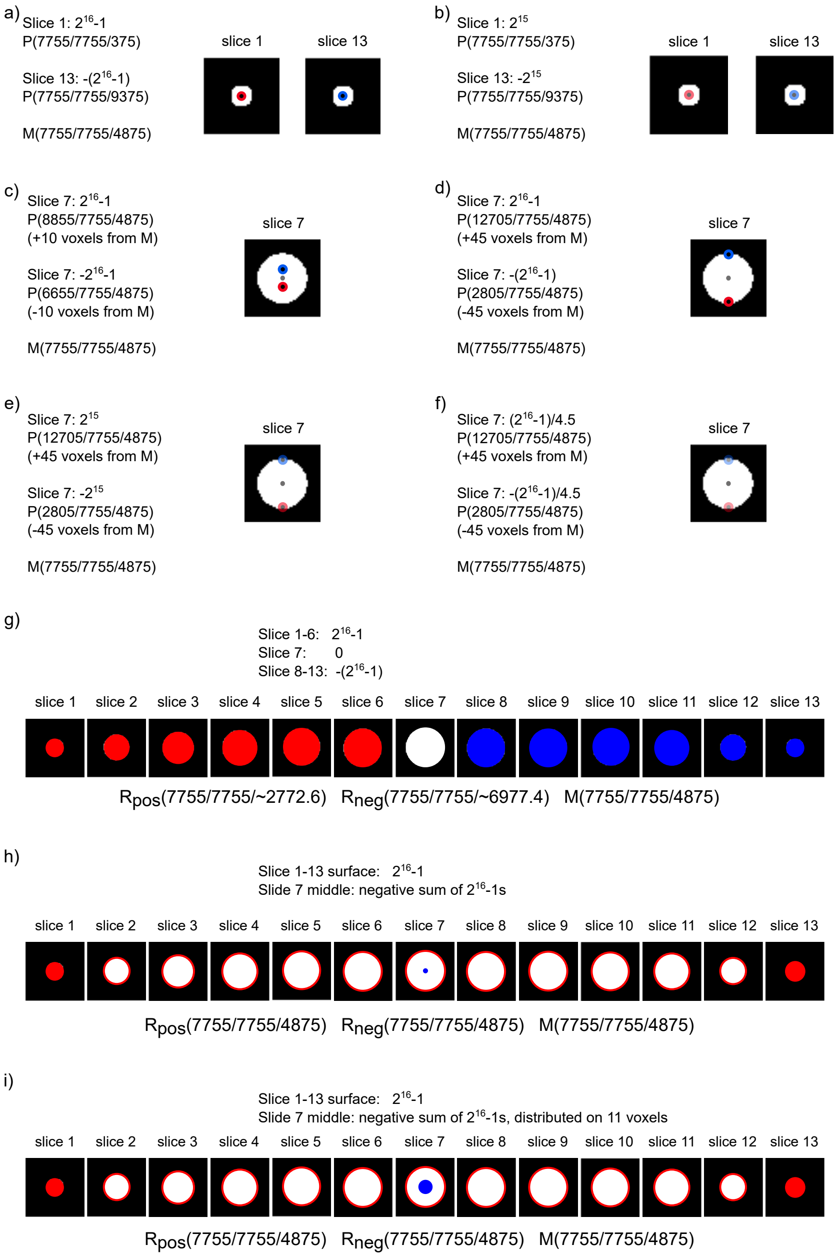

Fig. S35 Different charge scenarios: a) - f) single positive and negative charge placements. g) - i) several positive and negative charge placements.

j)

Slice 1 surface: $(2^{16}$-1)
Slice 2 surface: $(2^{16}$-1) / 13 * 12
Slice 3 surface: $(2^{16}$-1) / 13 * 11
Slice 4 surface: $(2^{16}$-1) / 13 * 10
Slice 5 surface: $(2^{16}$-1) / 13 * 9
Slice 6 surface: $(2^{16}$-1) / 13 * 8
Slice 7 surface: $(2^{16}$-1) / 13 * 7
Slice 8 surface: $(2^{16}$-1) / 13 * 6
Slice 9 surface: $(2^{16}$-1) / 13 * 5
Slice 10 surface: $(2^{16}$-1) / 13 * 4
Slice 11 surface: $(2^{16}$-1) / 13 * 3
Slice 12 surface: $(2^{16}$-1) / 13 * 2
Slice 13 surface: $(2^{16}$-1) / 13 * 1

Slide 7 middle: negative sum of 2^{16}-1s, 1 voxel

slice 1 slice 2 slice 3 slice 4 slice 5 slice 6 slice 7 slice 8 slice 9 slice 10 slice 11 slice 12 slice 13

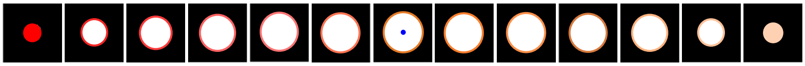

R_{pos}(7743.8/7743.8/3082.8) R_{neg}(7755/7755/4875) M(7755/7755/4875)

k)

Slice 1 surface: $(2^{16}$-1)
Slice 2 surface: $(2^{16}$-1) / 18 * 15
Slice 3 surface: $(2^{16}$-1) / 18 * 12
Slice 4 surface: $(2^{16}$-1) / 18 * 9
Slice 5 surface: $(2^{16}$-1) / 18 * 6
Slice 6 surface: $(2^{16}$-1) / 18 * 3
Slice 7 surface: 0
Slice 8 surface: -$(2^{16}$-1) / 18 * 3
Slice 9 surface: -$(2^{16}$-1) / 18 * 6
Slice 10 surface: -$(2^{16}$-1) / 18 * 9
Slice 11 surface: -$(2^{16}$-1) / 18 * 12
Slice 12 surface: -$(2^{16}$-1) / 18 * 15
Slice 13 surface: -$(2^{16}$-1)

Slide 7 middle: negative sum of $(2^{16}$-1) s, 1 voxel

slice 1 slice 2 slice 3 slice 4 slice 5 slice 6 slice 7 slice 8 slice 9 slice 10 slice 11 slice 12 slice 13

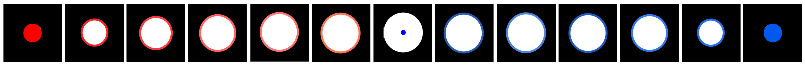

R_{pos}(7734.1/7734.1/1514.5) R_{neg}(7775.9/7775.9/8235.5) M(7755/7755/4875)

l)

Slice 1 surface: $(2^{16}$-1)
Slice 2 surface: $(2^{16}$-1) / 10 * 5
Slice 3 surface: $(2^{16}$-1) / 10 * 4
Slice 4 surface: $(2^{16}$-1) / 10 * 3
Slice 5 surface: $(2^{16}$-1) / 10 * 2
Slice 6 surface: $(2^{16}$-1) / 10 * 2
Slice 7 surface: $(2^{16}$-1) / 10 * 1
Slice 8 surface: -$(2^{16}$-1) / 10 * 1
Slice 9 surface: -$(2^{16}$-1) / 10 * 2
Slice 10 surface: -$(2^{16}$-1) / 10 * 3
Slice 11 surface: -$(2^{16}$-1) / 10 * 4
Slice 12 surface: -$(2^{16}$-1) / 10 * 7
Slice 13 surface: -$(2^{16}$-1) / 10 * 8

Slide 7 middle: negative sum of $(2^{16}$-1)s, 1 voxel

slice 1 slice 2 slice 3 slice 4 slice 5 slice 6 slice 7 slice 8 slice 9 slice 10 slice 11 slice 12 slice 13

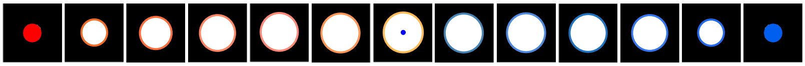

R_{pos}(7735.5/7735.5/1450.4) R_{neg}(7773.1/7773.1/8340.0) M(7755/7755/4875)

m)

Slice 1 surface: $(2^{16}$-1)
Slice 2 surface: $(2^{16}$-1)
Slice 3 surface: $(2^{16}$-1) / 10 * 4
Slice 4 surface: $(2^{16}$-1) / 10 * 3
Slice 5 surface: $(2^{16}$-1) / 10 * 2
Slice 6 surface: $(2^{16}$-1) / 10 * 2
Slice 7 surface: $(2^{16}$-1)/ 10 * 1
Slice 8 surface: -$(2^{16}$-1) / 10 * 1
Slice 9 surface: -$(2^{16}$-1) / 10 * 2
Slice 10 surface: -$(2^{16}$-1) / 10 * 3
Slice 11 surface: -$(2^{16}$-1) / 10 * 4
Slice 12 surface: -$(2^{16}$-1) / 10 * 7
Slice 13 surface: -$(2^{16}$-1) / 10 * 8

Slide 7 middle: negative sum of 2^{16}-1s, 1 voxel

slice 1 slice 2 slice 3 slice 4 slice 5 slice 6 slice 7 slice 8 slice 9 slice 10 slice 11 slice 12 slice 13

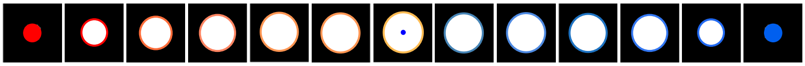

R_{pos}(7737.8/7737.8/1385.2) R_{neg}(7769.4/7769.4/7645.9) M(7755/7755/4875)

Fig. S36 Different charge scenarios: j) - m) several positive and negative charge placements.

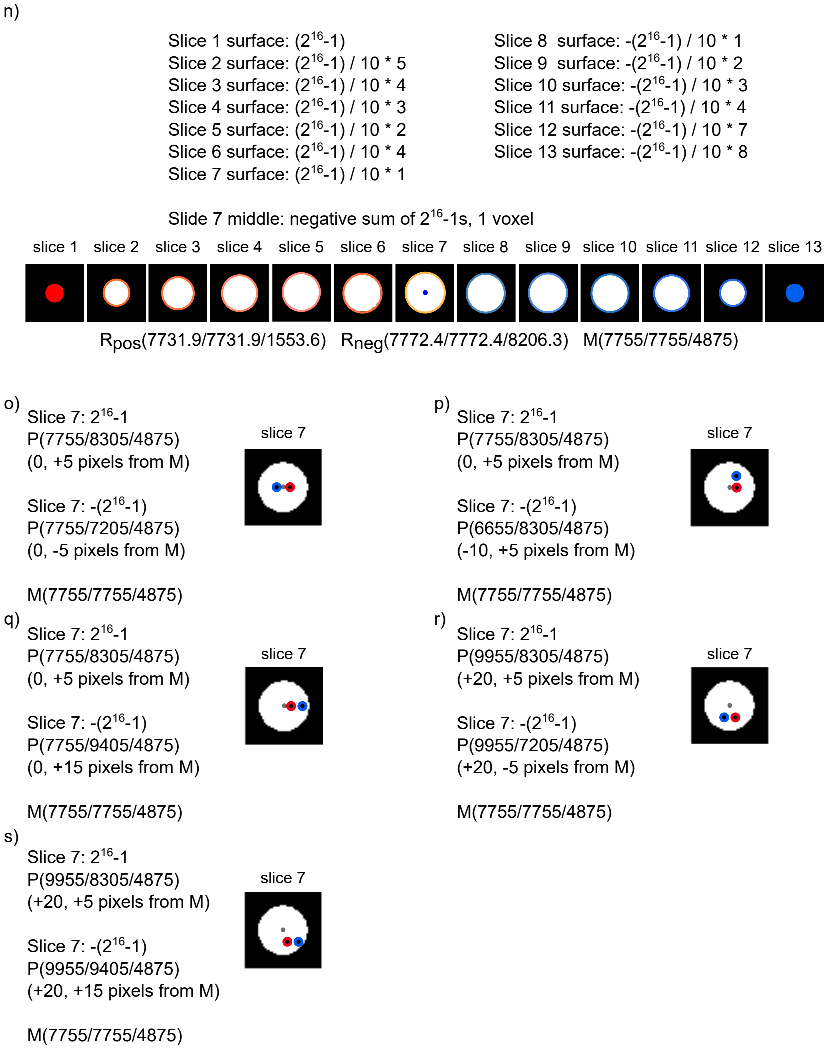

Fig. S37 Different charge scenarios: n) several positive and negative charge placements, o)-s) single charge placements with displacements around middle M

J.1.3 Two charges

a)
First scenario of two charges in lowest and highest layer of sphere, with highest and lowest charge possible.
Slice 1 in middle: $2^{16} - 1$, $R_{pos}(7755/7755/375)$
Slice 13 in middle: $-(2^{16} - 1)$, $R_{neg}(7755/7755/9375)$
Middle: M(7755/7755/4875)
The situation can be seen visualized in Fig. S35 a).

Table S8 Scenario a) Two charges in lowest and highest layer of sphere with highest and lowest charge possible.

Parameter	Analytic	Algorithm
q_+ $(\frac{1}{nm^3})$	$7.2 \cdot 10^{-3}$	$7.2 \cdot 10^{-3}$
$\lvert\mathbf{MR_{neg}}\rvert$ (nm)	4500	4500
$\lvert\mathbf{MR_{pos}}\rvert$ (nm)	4500	4500
$\lvert\mathbf{R_{pos}R_{neg}}\rvert$ (nm)	9000	9000
n_{pos}	1	1
R_{n1}	0.9	0.9
R_{n2}	0.9	0.91
q_{n1}	$3.5 \cdot 10^{-5}$	$3.5 \cdot 10^{-5}$
q_{n2}	1	1
q_{n3}	1	1
q_{n4}	$3.5 \cdot 10^{-5}$	$3.5 \cdot 10^{-5}$
(q_{n5})	$3.5 \cdot 10^{-5}$	$3.5 \cdot 10^{-5}$
q_{n6}	not practical	Inf
P_{n11}	$3.11 \cdot 10^{-5}$	$3.11 \cdot 10^{-5}$
P_{n21}	$3.11 \cdot 10^{-5}$	$3.14 \cdot 10^{-5}$
P_{n12}	0.9	0.9
P_{n22}	0.9	0.91
P_{n13}	0.9	0.9
P_{n23}	0.9	0.91
P_{n14}	$3.11 \cdot 10^{-5}$	$3.11 \cdot 10^{-5}$
P_{n24}	$3.11 \cdot 10^{-5}$	$3.14 \cdot 10^{-5}$
(P_{n15})	$3.11 \cdot 10^{-5}$	$3.11 \cdot 10^{-5}$
(P_{n25})	$3.11 \cdot 10^{-5}$	$3.14 \cdot 10^{-5}$
P_{n16}	not practical	Inf
P_{n26}	not practical	Inf

All in all scenario a) shows good agreement between analytic and algorithm solution. Deviation is seen due to translation from metric to ordinal scale (R_{n2}) leading to deviations in all P_n values starting with 2 indicative of R_{n2} being multiplied with the normalized charge. q_{n6} is not practical as we look at the artificial setting of starting with a total intensity of 0, leading to P_n values with P_{nx6} being at infinity.

b)
Slice 1 in middle: 2^{15}, $R_{pos}(7755/7755/375)$
Slice 13 in middle: -2^{15}, $R_{neg}(7755/7755/9375)$
Middle: M(7755/7755/4875)
Expected: Only difference in charge, which is reduced by factor 2. Thus, normalized dipole moment's value is expected to half.
The situation can be seen visualized in Fig. S35 b).

Table S9 *

Scenario b) Two charges in lowest and highest layer of sphere with $+2^{15}$ and -2^{15} as charges.

Parameter	Analytic	Algorithm
q_+ $(\frac{1}{nm^3})$	$3.6 \cdot 10^{-3}$	$3.6 \cdot 10^{-3}$
$\lvert\mathbf{MR_{neg}}\rvert$ (nm)	4500	4500
$\lvert\mathbf{MR_{pos}}\rvert$ (nm)	4500	4500
$\lvert\mathbf{R_{pos}R_{neg}}\rvert$ (nm)	9000	9000
n_{pos}	1	1
R_{n1}	0.9	0.9
R_{n2}	0.9	0.91
q_{n1}	$1.7 \cdot 10^{-5}$	$1.7 \cdot 10^{-5}$
q_{n2}	0.5	0.5
q_{n3}	1	1
q_{n4}	$3.5 \cdot 10^{-5}$	$3.5 \cdot 10^{-5}$
(q_{n5})	$3.5 \cdot 10^{-5}$	$3.5 \cdot 10^{-5}$
q_{n6}	not practical	Inf
P_{n11}	$1.56 \cdot 10^{-5}$	$1.56 \cdot 10^{-5}$
P_{n21}	$1.56 \cdot 10^{-5}$	$1.57 \cdot 10^{-5}$
P_{n12}	0.45	0.45
P_{n22}	0.45	0.45
P_{n13}	0.9	0.9
P_{n23}	0.9	0.91
P_{n14}	$3.11 \cdot 10^{-5}$	$3.11 \cdot 10^{-5}$
P_{n24}	$3.11 \cdot 10^{-5}$	$3.14 \cdot 10^{-5}$
(P_{n15})	$3.11 \cdot 10^{-5}$	$3.11 \cdot 10^{-5}$
(P_{n25})	$3.11 \cdot 10^{-5}$	$3.14 \cdot 10^{-5}$
P_{n16}	not practical	Inf
P_{n26}	not practical	Inf

All in all scenario b) shows good agreement between analytic and algorithm solution. Deviation is seen due to translation from metric to ordinal scale (R_{n2}) leading to deviations in all P_n values starting with 2 indicative of R_{n2} being multiplied with the normalized charge. q_{n6} is not practical as we look at the artificial setting of starting with a total intensity of 0, leading to P_n values with P_{nx6} being at infinity.

c)
Slice 7: $2^{16} - 1$, $R_{pos}(8855/7755/4875)$
(+10 voxels from M)
Slice 7: $-(2^{16} - 1)$, $R_{neg}(6655/7755/4875)$
(-10 voxels from M)
Middle: M(7755/7755/4875)
The situation can be seen visualized in Fig. S35 c).

Table S10 *

Scenario c) Two charges in distance of 10 voxels to the middle **M** opposite each other

Parameter	Analytic	Algorithm
q_+ $(\frac{1}{nm^3})$	$7.2 \cdot 10^{-3}$	$7.2 \cdot 10^{-3}$
$\lvert\mathbf{MR_{neg}}\rvert$ (nm)	1100	1100
$\lvert\mathbf{MR_{pos}}\rvert$ (nm)	1100	1100
$\lvert\mathbf{R_{pos}R_{neg}}\rvert$ (nm)	2200	2200
n_{pos}	1	1
R_{n1}	0.22	0.22
R_{n2}	0.22	0.22
q_{n1}	$3.5 \cdot 10^{-5}$	$3.5 \cdot 10^{-5}$
q_{n2}	1	1
q_{n3}	1	1
q_{n4}	$3.5 \cdot 10^{-5}$	$3.5 \cdot 10^{-5}$
(q_{n5})	$3.5 \cdot 10^{-5}$	$3.5 \cdot 10^{-5}$
q_{n6}	not practical	Inf
P_{n11}	$7.61 \cdot 10^{-6}$	$7.61 \cdot 10^{-6}$
P_{n21}	$7.61 \cdot 10^{-6}$	$7.68 \cdot 10^{-6}$
P_{n12}	0.22	0.22
P_{n22}	0.22	≈ 0.22
P_{n13}	0.22	0.22
P_{n23}	0.22	≈ 0.22
P_{n14}	$7.61 \cdot 10^{-6}$	$7.61 \cdot 10^{-6}$
P_{n24}	$7.61 \cdot 10^{-6}$	$7.68 \cdot 10^{-6}$
(P_{n15})	$7.61 \cdot 10^{-6}$	$7.61 \cdot 10^{-6}$
(P_{n25})	$7.61 \cdot 10^{-6}$	$7.68 \cdot 10^{-6}$
P_{n16}	not practical	Inf
P_{n26}	not practical	Inf

All in all scenario c) shows good agreement between analytic and algorithm solution. Deviation is seen due to translation from metric to ordinal scale (R_{n1} and R_{n2}) leading to deviations in all P_n values starting with 1 and 2 indicative of R_{n1}/R_{n2} being multiplied with the normalized charge. q_{n6} is not practical as we look at the artificial setting of starting with a total intensity of 0, leading to P_n values with P_{nx6} being at infinity.

d)
Slice 7: $2^{16} - 1$, $R_{pos}(12705/7755/4875)$
(+45 voxels from M)
Slice 7: $-(2^{16} - 1)$, $R_{neg}(2805/7755/4875)$
(-45 voxels from M)
Middle: M(7755/7755/4875)
The situation can be seen visualized in Fig. S35 d).

Table S11 *

Scenario d) Two charges in distance of 45 voxels to the middle **M** opposite each other.

Parameter	Analytic	Algorithm
q_+ $(\frac{1}{nm^3})$	$7.2 \cdot 10^{-3}$	$7.2 \cdot 10^{-3}$
$\lvert\mathbf{MR_{neg}}\rvert$ (nm)	4950	4950
$\lvert\mathbf{MR_{pos}}\rvert$ (nm)	4950	4950
$\lvert\mathbf{R_{pos}R_{neg}}\rvert$ (nm)	9900	9900
n_{pos}	1	1
R_{n1}	0.99	0.99
R_{n2}	0.99	1.0
q_{n1}	$3.5 \cdot 10^{-5}$	$3.5 \cdot 10^{-5}$
q_{n2}	1	1
q_{n3}	1	1
q_{n4}	$3.5 \cdot 10^{-5}$	$3.5 \cdot 10^{-5}$
(q_{n5})	$3.5 \cdot 10^{-5}$	$3.5 \cdot 10^{-5}$
q_{n6}	not practical	Inf
P_{n11}	$3.43 \cdot 10^{-5}$	$3.43 \cdot 10^{-5}$
P_{n21}	$3.43 \cdot 10^{-5}$	$3.45 \cdot 10^{-5}$
P_{n12}	0.99	0.99
P_{n22}	0.99	1.0
P_{n13}	0.99	0.99
P_{n23}	0.99	1.0
P_{n14}	$3.43 \cdot 10^{-5}$	$3.43 \cdot 10^{-5}$
P_{n24}	$3.43 \cdot 10^{-5}$	$3.45 \cdot 10^{-5}$
(P_{n15})	$3.43 \cdot 10^{-5}$	$3.43 \cdot 10^{-5}$
(P_{n25})	$3.43 \cdot 10^{-5}$	$3.45 \cdot 10^{-5}$
P_{n16}	not practical	Inf
P_{n26}	not practical	Inf

All in all scenario d) shows good agreement between analytic and algorithm solution. Deviation is seen due to translation from metric to ordinal scale (R_{n1} and R_{n2}) leading to deviations in all P_n values starting with 1 and 2 indicative of R_{n1}/R_{n2} being multiplied with the normalized charge. q_{n6} is not practical as we look at the artificial setting of starting with a total intensity of 0, leading to P_n values with P_{nx6} being at infinity.

e)
Slice 7: 2^{15}, $R_{pos}(12705/7755/4875)$
(+45 voxels from M)
Slice 7: -2^{15}, $R_{neg}(2805/7755/4875)$
(-45 voxels from M)
Middle: M(7755/7755/4875)
The situation can be seen visualized in Fig. S35 e).

Table S12 *

Scenario e) Two charges in distance of 45 voxels to the middle **M** opposite each other, with 2^{15} and -2^{15} as value per charge

Parameter	Analytic	Algorithm
q_+ $(\frac{1}{nm^3})$	$3.6 \cdot 10^{-3}$	$3.6 \cdot 10^{-3}$
$\lvert\mathbf{MR_{neg}}\rvert$ (nm)	4950	4950
$\lvert\mathbf{MR_{pos}}\rvert$ (nm)	4950	4950
$\lvert\mathbf{R_{pos}R_{neg}}\rvert$ (nm)	9900	9900
n_{pos}	1	1
R_{n1}	0.99	0.99
R_{n2}	0.99	1.0
q_{n1}	$1.7 \cdot 10^{-5}$	$1.7 \cdot 10^{-5}$
q_{n2}	0.5	0.5
q_{n3}	1	1
q_{n4}	$3.5 \cdot 10^{-5}$	$3.5 \cdot 10^{-5}$
(q_{n5})	$3.5 \cdot 10^{-5}$	$3.5 \cdot 10^{-5}$
q_{n6}	not practical	Inf
P_{n11}	$1.71 \cdot 10^{-5}$	$1.71 \cdot 10^{-5}$
P_{n21}	$1.71 \cdot 10^{-5}$	$1.73 \cdot 10^{-5}$
P_{n12}	0.50	0.50
P_{n22}	0.50	0.50
P_{n13}	0.99	0.99
P_{n23}	0.99	1.00
P_{n14}	$3.43 \cdot 10^{-5}$	$3.43 \cdot 10^{-5}$
P_{n24}	$3.43 \cdot 10^{-5}$	$3.45 \cdot 10^{-5}$
(P_{n15})	$3.43 \cdot 10^{-5}$	$3.43 \cdot 10^{-5}$
(P_{n25})	$3.43 \cdot 10^{-5}$	$3.45 \cdot 10^{-5}$
P_{n16}	not practical	Inf
P_{n26}	not practical	Inf

All in all scenario e) shows good agreement between analytic and algorithm solution. Deviation is seen due to translation from metric to ordinal scale (R_{n1} and R_{n2}) leading to deviations in all P_n values starting with 1 and 2 indicative of R_{n1}/R_{n2} being multiplied with the normalized charge. q_{n6} is not practical as we look at the artificial setting of starting with a total intensity of 0, leading to P_n values with P_{nx6} being at infinity.

f)
Slice 7: $(2^{16}-1)/4.5$, $R_{pos}(12705/7755/4875)$
(+45 voxels from M)
Slice 7: $-(2^{16}-1)/4.5$, $R_{neg}(2805/7755/4875)$
(-45 voxels from M)
Middle: M(7755/7755/4875)
The situation can be seen visualized in Fig. S35 f).

Table S13 *

Scenario f) Two charges in distance of 45 voxels to the middle **M** opposite each other, with $(2^{16}-1)/4.5$ and $(-2^{16}-1)/4.5$ as value per charge

Parameter	Analytic	Algorithm
q_+ $(\frac{1}{nm^3})$	$1.6 \cdot 10^{-3}$	$1.6 \cdot 10^{-3}$
$\lvert\mathbf{MR_{neg}}\rvert$ (nm)	4950	4950
$\lvert\mathbf{MR_{pos}}\rvert$ (nm)	4950	4950
$\lvert\mathbf{R_{pos}R_{neg}}\rvert$ (nm)	9900	9900
n_{pos}	1	1
R_{n1}	0.99	0.99
R_{n2}	0.99	1.00
q_{n1}	$7.69 \cdot 10^{-6}$	$\approx 7.69 \cdot 10^{-6}$
q_{n2}	0.22	0.22
q_{n3}	1	1
q_{n4}	$3.5 \cdot 10^{-5}$	$3.5 \cdot 10^{-5}$
(q_{n5})	$3.5 \cdot 10^{-5}$	$3.5 \cdot 10^{-5}$
q_{n6}	not practical	Inf
P_{n11}	$7.61 \cdot 10^{-6}$	$7.61 \cdot 10^{-6}$
P_{n21}	$7.61 \cdot 10^{-6}$	$7.68 \cdot 10^{-6}$
P_{n12}	0.22	0.22
P_{n22}	0.22	0.22
P_{n13}	0.99	0.99
P_{n23}	0.99	1.00
P_{n14}	$3.43 \cdot 10^{-5}$	$3.43 \cdot 10^{-5}$
P_{n24}	$3.43 \cdot 10^{-5}$	$3.45 \cdot 10^{-5}$
(P_{n15})	$3.43 \cdot 10^{-5}$	$3.43 \cdot 10^{-5}$
(P_{n25})	$3.43 \cdot 10^{-5}$	$3.45 \cdot 10^{-5}$
P_{n16}	not practical	Inf
P_{n26}	not practical	Inf

All in all scenario f) shows good agreement between analytic and algorithm solution. Deviation is seen due to translation from metric to ordinal scale (R_{n1} and R_{n2}) leading to deviations in all P_n values starting with 1 and 2 indicative of R_{n1}/R_{n2} being multiplied with the normalized charge. q_{n6} is not practical as we look at the artificial setting of starting with a total intensity of 0, leading to P_n values with P_{nx6} being at infinity.

J.1.4 Multiple charges

Calculating R_{pos} and R_{neg} analytically is at some part not possible any more for multiple charges. One needs to trust the program after previous results. Exact values are not possible as we move from metric scale to ordinal scale. Thus, there is little variation of exact analytical value. The same holds true for q_+.

g)
Slice 1-6: $2^{16} - 1$
Slice 7: 0
Slice 8-13: $-(2^{16} - 1)$
$R_{pos}(\approx 7755/\approx 7755/\approx 2772.6)$
$R_{neg}(\approx 7755/\approx 7755/\approx 6977.4)$
Middle: M(7755/7755/4875)
The situation can be seen visualized in Fig. S35 g).

Table S14 *

Scenario g) Slices 1-6 with charge value $2^{16} - 1$, Slice 7: 0 charge, Slice 8-13 with charge value $-(2^{16} - 1)$

Parameter	Analytic	Algorithm
q_+ ($\frac{1}{nm^3}$)		185.2
$\lvert\mathbf{MR_{neg}}\rvert$ (nm)	2102.4	2102.4
$\lvert\mathbf{MR_{pos}}\rvert$ (nm)	2102.4	2102.4
$\lvert\mathbf{R_{pos}R_{neg}}\rvert$ (nm)	4204.8	4204.7
n_{pos}		32151
R_{n1}	0.42	0.42
R_{n2}	0.42	0.42
q_{n1}	0.89	0.89
q_{n2}	0.80	0.80
q_{n3}	0.80	0.80
q_{n4}	0.89	0.89
(q_{n5})	$1.11 \cdot 10^{-5}$	$1.11 \cdot 10^{-5}$
q_{n6}	not practical	$-4.3 \cdot 10^5$
P_{n11}	0.37	0.37
P_{n21}	0.37	0.38
P_{n12}	0.34	0.34
P_{n22}	0.34	0.34
P_{n13}	0.34	0.34
P_{n23}	0.34	0.34
P_{n14}	0.37	0.37
P_{n24}	0.37	0.38
(P_{n15})	0.47	0.47
(P_{n25})	0.47	0.47
P_{n16}	not practical	$-1.8 \cdot 10^5$
P_{n26}	not practical	$-1.8 \cdot 10^5$

All in all scenario g) shows good agreement between analytic and algorithm solution. Deviation is seen due to translation from metric to ordinal scale (R_{n1} and R_{n2}) leading to deviations in all P_n values starting with 1 and 2 indicative of R_{n1}/R_{n2} being multiplied with the normalized charge. q_{n6} is not practical

as we look at the artificial setting of starting with a total intensity of roughly $\approx$-3864.98 , leading to P_n values with P_{nx6} being at high negative value.

h)
Slice 1-13: surface $2^{16}-1$
Slice 7 middle: negative sum of $2^{16}-1$s
R_{pos}(≈7755/≈7755/≈4875)
R_{neg}(7755/7755/4875)
Middle: M(7755/7755/4875)
The situation can be seen visualized in Fig. S35 h).

Table S15 *

Scenario h) Surface filled with charges of value $2^{16}-1$, middle of slice 7: negative sum of all positive charge values.

Parameter	Analytic	Algorithm
q_+ $(\frac{1}{nm^3})$		110.1
$\|\mathbf{MR_{neg}}\|$ (nm)	0	0
$\|\mathbf{MR_{pos}}\|$ (nm)	0	$3.7 \cdot 10^{-9}$
$\|\mathbf{R_{pos}R_{neg}}\|$ (nm)	0	$3.7 \cdot 10^{-9}$
n_{pos}		15242
R_{n1}	0	$3.75 \cdot 10^{-13}$
R_{n2}	0	$3.78 \cdot 10^{-13}$
q_{n1}	0.53	0.53
q_{n2}	1	1
q_{n3}	1	1
q_{n4}	0.53	0.53
(q_{n5})	0.53	0.53
q_{n6}	not practical	Inf
P_{n11}	0	$1.6 \cdot 10^{-13}$
P_{n21}	0	$1.6 \cdot 10^{-13}$
P_{n12}	0	$3.0 \cdot 10^{-13}$
P_{n22}	0	$3.1 \cdot 10^{-13}$
P_{n13}	0	$3.1 \cdot 10^{-13}$
P_{n23}	0	$3.1 \cdot 10^{-13}$
P_{n14}	0	$1.6 \cdot 10^{-13}$
P_{n24}	0	$1.6 \cdot 10^{-13}$
(P_{n15})	0	$1.6 \cdot 10^{-13}$
(P_{n25})	0	$1.6 \cdot 10^{-13}$
P_{n16}	not practical	Inf
P_{n26}	not practical	Inf

All in all scenario h) shows good agreement between analytic and algorithm solution. Deviation is seen due to translation from metric to ordinal scale (R_{n1} and R_{n2}) leading to deviations in all P_n values starting with 1 and 2 indicative of R_{n1}/R_{n2} being multiplied with the normalized charge. q_{n6} is not practical as we look at the artificial setting of starting with a total intensity of 0 , leading to P_n values with P_{nx6} being at infinity.

i)
Slice 1-13: surface $2^{16}-1$
Slice 7 middle: negative sum of $2^{16}-1$s distributed on 11 voxels
$R_{pos}(\approx 7755/\approx 7755/\approx 4875)$
$R_{neg}(\approx 7755/\approx 7755/\approx 4875)$
Middle: M(7755/7755/4875)

The situation can be seen visualized in Fig. S35 i).

Table S16 *

Scenario i) Surface filled with charges of value $2^{16}-1$, middle of slice 7, distributed on 11 voxels: negative sum of all positive charge values.

Parameter	Analytic	Algorithm
q_+ $(\frac{1}{nm^3})$		110
$\lvert \mathbf{MR_{neg}} \rvert$ (nm)	0	
$\lvert \mathbf{MR_{pos}} \rvert$ (nm)	0	
$\lvert \mathbf{R_{pos}R_{neg}} \rvert$ (nm)	0	
n_{pos}		15242
R_{n1}	0	$3.6 \cdot 10^{-13}$
R_{n2}	0	$3.7 \cdot 10^{-13}$
q_{n1}	0.53	0.53
q_{n2}	1	1
q_{n3}	1	1
q_{n4}	0.53	0.53
(q_{n5})	0.53	0.53
q_{n6}	not practical	$10 \cdot 10^{8}$
P_{n11}	0	$1.9 \cdot 10^{-13}$
P_{n21}	0	$1.9 \cdot 10^{-13}$
P_{n12}	0	$3.6 \cdot 10^{-13}$
P_{n22}	0	$3.7 \cdot 10^{-13}$
P_{n13}	0	$3.6 \cdot 10^{-13}$
P_{n23}	0	$3.7 \cdot 10^{-13}$
P_{n14}	0	$1.9 \cdot 10^{-13}$
P_{n24}	0	$1.9 \cdot 10^{-13}$
(P_{n15})	0	$1.9 \cdot 10^{-13}$
(P_{n25})	0	$1.9 \cdot 10^{-13}$
P_{n16}	not practical	$3.6 \cdot 10^{-4}$
P_{n26}	not practical	$3.7 \cdot 10^{-4}$

All in all scenario i) shows good agreement between analytic and algorithm solution. Deviation is seen due to translation from metric to ordinal scale (R_{n1} and R_{n2}) leading to deviations in all P_n values starting with 1 and 2 indicative of R_{n1}/R_{n2} being multiplied with the normalized charge. q_{n6} is not practical as we look at the artificial setting of starting with a total intensity of slightly positive value, leading to P_n values with P_{nx6} being at high positive value.

j)

Table S17 *

Scenario j) Surface filled with charges of decreasing value of $2^{16} - 1$ (still positive) per increasing slice number, middle of slice 7: negative sum of all positive charge values.

Slice 1: surface $2^{16} - 1$	Slice 8: surface $(2^{16} - 1)/13 * 6$
Slice 2: surface $(2^{16} - 1)/13 * 12$	Slice 9: surface $(2^{16} - 1)/13 * 5$
Slice 3: surface $(2^{16} - 1)/13 * 11$	Slice 10: surface $(2^{16} - 1)/13 * 4$
Slice 4: surface $(2^{16} - 1)/13 * 10$	Slice 11: surface $(2^{16} - 1)/13 * 3$
Slice 5: surface $(2^{16} - 1)/13 * 9$	Slice 12: surface $(2^{16} - 1)/13 * 2$
Slice 6: surface $(2^{16} - 1)/13 * 8$	Slice 13: surface $(2^{16} - 1)/13 * 1$
Slice 7: surface $(2^{16} - 1)/13 * 7$	

Slice 7 middle: negative sum of $2^{16} - 1$s
R_{pos}($\approx$ 7743.8/ $\approx$7743.8/$\approx$3082.8)
R_{neg}($\approx$7755/$\approx$7755/$\approx$4875)
Middle: M(7755/7755/4875)
$\mathbf{MR_{pos}}$ and $\mathbf{R_{pos}R_{neg}}$ calculated with rounded values taken from algorithm. Thus, there is already a difference of analytical solution to algorithm.
The situation can be seen visualized in Fig. S36 j).

All in all scenario j) shows good agreement between analytic and algorithm solution. Deviation is seen due to translation from metric to ordinal scale (R_{n1} and R_{n2}) leading to deviations in all P_n values starting with 1 and 2 indicative of R_{n1}/R_{n2} being multiplied with the normalized charge. q_{n6} is not practical as we look at the artificial setting of starting with a total intensity of 0, leading to P_n values with P_{nx6} being at infinity.

Table S18 *

Scenario j) Surface filled with charges of decreasing value of $2^{16}-1$ (still positive) per increasing slice number, middle of slice 7: negative sum of all positive charge values.

Parameter	Analytic	Algorithm
q_+ $(\frac{1}{nm^3})$		
$\|\mathbf{MR_{neg}}\|$ (nm)		59.3
$\|\mathbf{MR_{pos}}\|$ (nm)	0	0
$\|\mathbf{R_{pos}R_{neg}}\|$ (nm)	1792.3	1792.3
n_{pos}		15242
R_{n1}	0.18	0.18
R_{n2}	0.18	0.18
q_{n1}	0.28	0.28
q_{n2}	0.54	0.54
q_{n3}	0.54	0.54
q_{n4}	0.28	0.28
(q_{n5})	0.53	0.53
q_{n6}	not practical	Inf
P_{n11}	0.05	0.05
P_{n21}	0.05	0.05
P_{n12}	0.10	0.10
P_{n22}	0.10	0.10
P_{n13}	0.10	0.10
P_{n23}	0.10	0.10
P_{n14}	0.05	0.05
P_{n24}	0.05	0.05
(P_{n15})	0.09	0.09
(P_{n25})	0.09	0.10
P_{n16}	not practical	Inf
P_{n26}	not practical	Inf

k)

Table S19 *

Scenario k) Surface filled with charges of decreasing value of $2^{16}-1$ (decreasing evenlty till $-(2^{16}-1)$) per increasing slice number, middle of slice 7: charge value for total charge obtaining value 0.

Slice 1: surface $2^{16}-1$	Slice 8: surface $-(2^{16}-1)/18*3$
Slice 2: surface $(2^{16}-1)/18*15$	Slice 9: surface $-(2^{16}-1)/18*6$
Slice 3: surface $(2^{16}-1)/18*12$	Slice 10: surface $-(2^{16}-1)/18*9$
Slice 4: surface $(2^{16}-1)/18*9$	Slice 11: surface $-(2^{16}-1)/18*12$
Slice 5: surface $(2^{16}-1)/18*6$	Slice 12: surface $-(2^{16}-1)/18*15$
Slice 6: surface $(2^{16}-1)/18*3$	Slice 13: surface $-(2^{16}-1)$
Slice 7: surface 0	

Slice 7 middle: negative sum of $2^{16}-1$s in 1 voxel
$R_{pos}(7734.1/7734.1/1514.5)$
$R_{neg}(7775.9/7775.9/8235.5)$
Middle: M(7755/7755/4875)
The situation can be seen visualized in Fig. S36 k).

All in all scenario k) shows good agreement between analytic and algorithm solution. Deviation is seen due to translation from metric to ordinal scale (R_{n1} and R_{n2}) leading to deviations in all P_n values starting with 1 and 2 indicative of R_{n1}/R_{n2} being multiplied with the normalized charge. q_{n6} is not practical as we look at the artificial setting of starting with a total intensity of 0, leading to P_n values with P_{nx6} being at infinity.

Table S20 *

Scenario k) Surface filled with charges of decreasing value of $2^{16} - 1$ (decreasing evenlty till $-(2^{16} - 1)$) per increasing slice number, middle of slice 7: charge value for total charge obtaining value 0.

Parameter	Analytic	Algorithm
$q_+ \ (\frac{1}{nm^3})$		34.2
$\|\mathbf{MR_{neg}}\| \ (nm)$	3360.6	3360.7
$\|\mathbf{MR_{pos}}\| \ (nm)$	3360.6	3360.7
$\|\mathbf{R_{pos}R_{neg}}\| \ (nm)$	6721.3	6721.3
n_{pos}		7386
R_{n1}	0.67	0.67
R_{n2}	0.67	0.68
q_{n1}	0.16	0.16
q_{n2}	0.64	0.64
q_{n3}	0.64	0.64
q_{n4}	0.16	0.16
(q_{n5})	0.26	0.26
q_{n6}	not practical	Inf
P_{n11}	0.11	0.11
P_{n21}	0.11	0.11
P_{n12}	0.43	0.43
P_{n22}	0.43	0.44
P_{n13}	0.43	0.43
P_{n23}	0.43	0.44
P_{n14}	0.11	0.11
P_{n24}	0.11	0.11
(P_{n15})	0.17	0.17
(P_{n25})	0.17	0.17
P_{n16}	not practical	Inf
P_{n26}	not practical	Inf

l)

Table S21 *

Scenario l) Surface filled with charges of decreasing value of $2^{16}-1$ per increasing slice number, middle of slice 7: charge value for receiving total charge value 0.

Slice 1: surface $(2^{16}-1)$	Slice 8: surface $-(2^{16}-1)/10*1$
Slice 2: surface $(2^{16}-1)/10*5$	Slice 9: surface $-(2^{16}-1)/10*2$
Slice 3: surface $(2^{16}-1)/10*4$	Slice 10: surface $-(2^{16}-1)/10*3$
Slice 4: surface $(2^{16}-1)/10*3$	Slice 11: surface $-(2^{16}-1)/10*4$
Slice 5: surface $(2^{16}-1)/10*2$	Slice 12: surface $-(2^{16}-1)/10*7$
Slice 6: surface $(2^{16}-1)/10*2$	Slice 13: surface $-(2^{16}-1)/10*8$
Slice 7: surface $(2^{16}-1)/10*1$	

Slice 7 middle: negative sum of $2^{16}-1$s in 1 voxel
$R_{pos}(\approx 7735.5/\approx 7735.5/\approx 1450.4)$
$R_{neg}(\approx 7773.1/\approx 7773.1/\approx 8340.0)$
Middle: M(7755/7755/4875)
The situation can be seen visualized in Fig. S36 l).

All in all scenario l) shows good agreement between analytic and algorithm solution. Deviation is seen due to translation from metric to ordinal scale (R_{n1} and R_{n2}) leading to deviations in all P_n values starting with 1 and 2 indicative of R_{n1}/R_{n2} being multiplied with the normalized charge. q_{n6} is not practical as we look at the artificial setting of starting with a total intensity of 0, leading to P_n values with P_{nx6} being at infinity.

Table S22 *

Scenario l) Surface filled with charges of decreasing value of $2^{16} - 1$ per increasing slice number, middle of slice 7: charge value for receiving total charge value 0.

Parameter	Analytic	Algorithm
q_+ ($\frac{1}{nm^3}$)		24.9
$\lvert\mathbf{MR_{neg}}\rvert$ (nm)	3465.1	3465.1
$\lvert\mathbf{MR_{pos}}\rvert$ (nm)	3424.7	3424.7
$\lvert\mathbf{R_{pos}R_{neg}}\rvert$ (nm)	6889.8	6889.8
n_{pos}		7856
R_{n1}	0.69	0.69
R_{n2}	0.69	0.69
q_{n1}	0.12	0.12
q_{n2}	0.44	0.44
q_{n3}	0.44	0.44
q_{n4}	0.12	0.12
(q_{n5})	0.27	0.27
q_{n6}	not practical	Inf
P_{n11}	0.08	0.08
P_{n21}	0.08	0.08
P_{n12}	0.30	0.30
P_{n22}	0.30	0.31
P_{n13}	0.30	0.30
P_{n23}	0.30	0.31
P_{n14}	0.08	0.08
P_{n24}	0.08	0.08
(P_{n15})	0.19	0.19
(P_{n25})	0.19	0.19
P_{n16}	not practical	Inf
P_{n26}	not practical	Inf

m)

Table S23 *

Scenario m) Surface filled with charges of decreasing value of $2^{16}-1$ per increasing slice number, middle of slice 7: charge value for receiving total charge value 0.

Slice 1: surface $(2^{16}-1)$	Slice 8: surface $-(2^{16}-1)/10*1$
Slice 2: surface $(2^{16}-1)$	Slice 9: surface $-(2^{16}-1)/10*2$
Slice 3: surface $(2^{16}-1)/10*4$	Slice 10: surface $-(2^{16}-1)/10*3$
Slice 4: surface $(2^{16}-1)/10*3$	Slice 11: surface $-(2^{16}-1)/10*4$
Slice 5: surface $(2^{16}-1)/10*2$	Slice 12: surface $-(2^{16}-1)/10*7$
Slice 6: surface $(2^{16}-1)/10*2$	Slice 13: surface $-(2^{16}-1)/10*8$
Slice 7: surface $(2^{16}-1)/10*1$	

Slice 7 middle: negative sum of $2^{16}-1$s in 1 voxel
$R_{pos}(\approx 7737.8/\approx 7737.8/\approx 1385.2)$
$R_{neg}(\approx 7769.4/\approx 7769.4/\approx 7645.9)$
Middle: M(7755/7755/4875)
The situation can be seen visualized in Fig. S36 m).

All in all scenario m) shows good agreement between analytic and algorithm solution. Deviation is seen due to translation from metric to ordinal scale (R_{n1} and R_{n2}) leading to deviations in all P_n values starting with 1 and 2 indicative of R_{n1}/R_{n2} being multiplied with the normalized charge. q_{n6} is not practical as we look at the artificial setting of starting with a total intensity of 0, leading to P_n values with P_{nx6} being at infinity.

Table S24 *

Scenario m) Surface filled with charges of decreasing value of $2^{16} - 1$ per increasing slice number, middle of slice 7: charge value for receiving total charge value 0.

Parameter	Analytic	Algorithm
q_+ ($\frac{1}{nm^3}$)		31.18
$\lvert\mathbf{MR_{neg}}\rvert$ (nm)	2771.0	2770.9
$\lvert\mathbf{MR_{pos}}\rvert$ (nm)	3489.9	3489.9
$\lvert\mathbf{R_{pos}R_{neg}}\rvert$ (nm)	6260.9	6260.8
n_{pos}		7856
R_{n1}	0.63	0.63
R_{n2}	0.63	0.63
q_{n1}	0.15	0.15
q_{n2}	0.55	0.55
q_{n3}	0.55	0.55
q_{n4}	0.15	0.15
(q_{n5})	0.27	0.27
q_{n6}	not practical	Inf
P_{n11}	0.09	0.09
P_{n21}	0.09	0.09
P_{n12}	0.34	0.34
P_{n22}	0.34	0.35
P_{n13}	0.34	0.34
P_{n23}	0.34	0.35
P_{n14}	0.09	0.09
P_{n24}	0.09	0.09
(P_{n15})	0.17	0.17
(P_{n25})	0.17	0.17
P_{n16}	not practical	Inf
P_{n26}	not practical	Inf

n)

Table S25 *

Scenario n) Surface filled with charges of decreasing value of $2^{16}-1$ per increasing slice number, middle of slice 7: charge value for receiving total charge value 0.

Slice 1: surface $(2^{16}-1)$	Slice 8: surface $-(2^{16}-1)/10*1$
Slice 2: surface $(2^{16}-1)/10*5$	Slice 9: surface $-(2^{16}-1)/10*2$
Slice 3: surface $(2^{16}-1)/10*4$	Slice 10: surface $-(2^{16}-1)/10*3$
Slice 4: surface $(2^{16}-1)/10*3$	Slice 11: surface $-(2^{16}-1)/10*4$
Slice 5: surface $(2^{16}-1)/10*2$	Slice 12: surface $-(2^{16}-1)/10*7$
Slice 6: surface $(2^{16}-1)/10*4$	Slice 13: surface $-(2^{16}-1)/10*8$
Slice 7: surface $(2^{16}-1)/10*1$	

Slice 7 middle: negative sum of $2^{16}-1$s in 1 voxel
$R_{pos}(\approx 7731.9/\approx 7731.9/\approx 1553.6)$
$R_{neg}(\approx 7772.4/\approx 7772.4/\approx 8206.3)$
Middle: M(7755/7755/4875)
The situation can be seen visualized in Fig. S37 n).

All in all scenario n) shows good agreement between analytic and algorithm solution. Deviation is seen due to translation from metric to ordinal scale (R_{n1} and R_{n2}) leading to deviations in all P_n values starting with 1 and 2 indicative of R_{n1}/R_{n2} being multiplied with the normalized charge. q_{n6} is not practical as we look at the artificial setting of starting with a total intensity of 0, leading to P_n values with P_{nx6} being at infinity.

Table S26 *

Scenario n) Surface filled with charges of decreasing value of $2^{16} - 1$ per increasing slice number, middle of slice 7: charge value for receiving total charge value 0.

Parameter	Analytic	Algorithm
q_+ $(\frac{1}{nm^3})$		25.9
$\lvert \mathbf{MR_{neg}} \rvert$ (nm)	3331.4	3331.4
$\lvert \mathbf{MR_{pos}} \rvert$ (nm)	3321.6	3321.6
$\lvert \mathbf{R_{pos}R_{neg}} \rvert$ (nm)	6652.9	6652.9
n_{pos}		7856
R_{n1}	0.67	0.67
R_{n2}	0.67	0.67
q_{n1}	0.12	0.12
q_{n2}	0.46	0.46
q_{n3}	0.46	0.46
q_{n4}	0.12	0.12
(q_{n5})	0.27	0.27
q_{n6}	not practical	Inf
P_{n11}	0.08	0.08
P_{n21}	0.08	0.08
P_{n12}	0.30	0.30
P_{n22}	0.30	0.31
P_{n13}	0.30	0.30
P_{n23}	0.30	0.31
P_{n14}	0.08	0.08
P_{n24}	0.08	0.08
(P_{n15})	0.18	0.18
(P_{n25})	0.18	0.18
P_{n16}	not practical	Inf
P_{n26}	not practical	Inf

J.1.5 Displacement

o)
Slice 7: $2^{16} - 1$, $R_{pos}(8305/7755/4875)$
(+5, 0 voxels from M)
Slice 7: $-(2^{16} - 1)$, $R_{neg}(7205/7755/4875)$
(-5, 0 voxels from M)
Middle: M(7755/7755/4875)
The situation can be seen visualized in Fig. S37 o).

Table S27 *

Scenario o) Two charges in distance of 5 voxels to the middle **M** opposite each other

Parameter	Analytic	Algorithm
q_+ $(\frac{1}{nm^3})$	$\approx 7.2 \cdot 10^{-3}$	$\approx 7.2 \cdot 10^{-3}$
$\|\mathbf{MR_{neg}}\|$ (nm)	550	550
$\|\mathbf{MR_{pos}}\|$ (nm)	550	550
$\|\mathbf{R_{pos}R_{neg}}\|$ (nm)	1100	1100
n_{pos}	1	1
R_{n1}	0.11	0.11
R_{n2}	0.11	0.11
q_{n1}	$\approx 3.5 \cdot 10^{-5}$	$\approx 3.5 \cdot 10^{-5}$
q_{n2}	1	1
q_{n3}	1	1
q_{n4}	$\approx 3.5 \cdot 10^{-5}$	$\approx 3.5 \cdot 10^{-5}$
(q_{n5})	$\approx 3.5 \cdot 10^{-5}$	$\approx 3.5 \cdot 10^{-5}$
q_{n6}	not practical	Inf
P_{n11}	$3.8 \cdot 10^{-6}$	$3.8 \cdot 10^{-6}$
P_{n21}	$3.8 \cdot 10^{-6}$	$3.8 \cdot 10^{-6}$
P_{n12}	0.11	0.11
P_{n22}	0.11	0.11
P_{n13}	0.11	0.11
P_{n23}	0.11	0.11
P_{n14}	$3.8 \cdot 10^{-6}$	$3.8 \cdot 10^{-6}$
P_{n24}	$3.8 \cdot 10^{-6}$	$3.8 \cdot 10^{-6}$
(P_{n15})	$3.8 \cdot 10^{-6}$	$3.8 \cdot 10^{-6}$
(P_{n25})	$3.8 \cdot 10^{-6}$	$3.8 \cdot 10^{-6}$
P_{n16}	not practical	Inf
P_{n26}	not practical	Inf

All in all scenario o) shows good agreement between analytic and algorithm solution. Deviation is seen due to translation from metric to ordinal scale (R_{n1} and R_{n2}) leading to deviations in all P_n values starting with 1 and 2 indicative of R_{n1}/R_{n2} being multiplied with the normalized charge. q_{n6} is not practical as we look at the artificial setting of starting with a total intensity of 0, leading to P_n values with P_{nx6} being at infinity.

p)
Slice 7: $2^{16} - 1$, $R_{pos}(8305/7755/4875)$
(+5, 0 voxels from M)
Slice 7: $-(2^{16} - 1)$, $R_{neg}(8305/6655/4875)$
(+5, -10 voxels from M)
Middle: M(7755/7755/4875)
The situation can be seen visualized in Fig. S37 p).

Table S28 *

Scenario p) Two charges in distance of 5 and -10/+5 voxels to the middle **M**

Parameter	Analytic	Algorithm
q_+ $(\frac{1}{nm^3})$	$7.2 \cdot 10^{-3}$	$7.2 \cdot 10^{-3}$
$\lvert\mathbf{MR_{neg}}\rvert$ (nm)	1229.8	1229.8
$\lvert\mathbf{MR_{pos}}\rvert$ (nm)	550	550
$\lvert\mathbf{R_{pos}R_{neg}}\rvert$ (nm)	1100	1100
n_{pos}	1	1
R_{n1}	0.11	0.11
R_{n2}	0.11	0.11
q_{n1}	$3.5 \cdot 10^{-5}$	$3.5 \cdot 10^{-5}$
q_{n2}	1	1
q_{n3}	1	1
q_{n4}	$3.5 \cdot 10^{-5}$	$3.5 \cdot 10^{-5}$
(q_{n5})	$3.5 \cdot 10^{-5}$	$3.5 \cdot 10^{-5}$
q_{n6}	not practical	Inf
P_{n11}	$3.8 \cdot 10^{-6}$	$3.8 \cdot 10^{-6}$
P_{n21}	$3.8 \cdot 10^{-6}$	$3.8 \cdot 10^{-6}$
P_{n12}	0.11	0.11
P_{n22}	0.11	0.11
P_{n13}	0.11	0.11
P_{n23}	0.11	0.11
P_{n14}	$3.8 \cdot 10^{-6}$	$3.8 \cdot 10^{-6}$
P_{n24}	$3.8 \cdot 10^{-6}$	$3.8 \cdot 10^{-6}$
(P_{n15})	$3.8 \cdot 10^{-6}$	$3.8 \cdot 10^{-6}$
(P_{n25})	$3.8 \cdot 10^{-6}$	$3.8 \cdot 10^{-6}$
P_{n16}	not practical	Inf
P_{n26}	not practical	Inf

All in all scenario p) shows good agreement between analytic and algorithm solution. Deviation is seen due to translation from metric to ordinal scale (R_{n1} and R_{n2}) leading to deviations in all P_n values starting with 1 and 2 indicative of R_{n1}/R_{n2} being multiplied with the normalized charge. q_{n6} is not practical as we look at the artificial setting of starting with a total intensity of 0, leading to P_n values with P_{nx6} being at infinity.

q)
Slice 7: $2^{16} - 1$, $R_{pos}(8305/7755/4875)$
(+5, 0 voxels from M)
Slice 7: $-(2^{16} - 1)$, $R_{neg}(9405/7755/4875)$
(+15, 0 voxels from M)
Middle: M(7755/7755/4875)
The situation can be seen visualized in Fig. S37 q).

Table S29 *

Scenario q) Two charges in distance of 5 and 15 voxels to the middle **M**

Parameter	Analytic	Algorithm
q_+ $(\frac{1}{nm^3})$	$7.2 \cdot 10^{-3}$	$7.2 \cdot 10^{-3}$
$\lvert\mathbf{MR_{neg}}\rvert$ (nm)	550	550
$\lvert\mathbf{MR_{pos}}\rvert$ (nm)	550	550
$\lvert\mathbf{R_{pos}R_{neg}}\rvert$ (nm)	1100	1100
n_{pos}	1	1
R_{n1}	0.11	0.11
R_{n2}	0.11	0.11
q_{n1}	$3.5 \cdot 10^{-5}$	$3.5 \cdot 10^{-5}$
q_{n2}	1	1
q_{n3}	1	1
q_{n4}	$3.5 \cdot 10^{-5}$	$3.5 \cdot 10^{-5}$
(q_{n5})	$3.5 \cdot 10^{-5}$	$3.5 \cdot 10^{-5}$
q_{n6}	not practical	Inf
P_{n11}	$3.8 \cdot 10^{-6}$	$3.8 \cdot 10^{-6}$
P_{n21}	$3.8 \cdot 10^{-6}$	$3.8 \cdot 10^{-6}$
P_{n12}	0.11	0.11
P_{n22}	0.11	0.11
P_{n13}	0.11	0.11
P_{n23}	0.11	0.11
P_{n14}	$3.8 \cdot 10^{-6}$	$3.8 \cdot 10^{-6}$
P_{n24}	$3.8 \cdot 10^{-6}$	$3.8 \cdot 10^{-6}$
(P_{n15})	$3.8 \cdot 10^{-6}$	$3.8 \cdot 10^{-6}$
(P_{n25})	$3.8 \cdot 10^{-6}$	$3.8 \cdot 10^{-6}$
P_{n16}	not practical	Inf
P_{n26}	not practical	Inf

All in all scenario q) shows good agreement between analytic and algorithm solution. Deviation is seen due to translation from metric to ordinal scale (R_{n1} and R_{n2}) leading to deviations in all P_n values starting with 1 and 2 indicative of R_{n1}/R_{n2} being multiplied with the normalized charge. q_{n6} is not practical as we look at the artificial setting of starting with a total intensity of 0, leading to P_n values with P_{nx6} being at infinity.

r)
Slice 7: $2^{16} - 1$, $R_{pos}(8305/9955/4875)$
(+5, +20 voxels from M)
Slice 7: $-(2^{16} - 1)$, $R_{neg}(7205/9955/4875)$
(-5, +20 voxels from M)
Middle: M(7755/7755/4875)
The situation can be seen visualized in Fig. S37 r).

Table S30 *

Scenario r) Two charges in distance of 20/5 and 20/-5 voxels to the middle **M**

Parameter	Analytic	Algorithm
q_+ $(\frac{1}{nm^3})$	$7.2 \cdot 10^{-3}$	$7.2 \cdot 10^{-3}$
$\lvert\mathbf{MR_{neg}}\rvert$ (nm)	2267.7	2267.7
$\lvert\mathbf{MR_{pos}}\rvert$ (nm)	2267.7	2267.7
$\lvert\mathbf{R_{pos}R_{neg}}\rvert$ (nm)	1100	1100
n_{pos}	1	1
R_{n1}	0.11	0.11
R_{n2}	0.11	0.11
q_{n1}	$3.5 \cdot 10^{-5}$	$3.5 \cdot 10^{-5}$
q_{n2}	1	1
q_{n3}	1	1
q_{n4}	$3.5 \cdot 10^{-5}$	$3.5 \cdot 10^{-5}$
(q_{n5})	$3.5 \cdot 10^{-5}$	$3.5 \cdot 10^{-5}$
q_{n6}	not practical	Inf
P_{n11}	$3.8 \cdot 10^{-6}$	$3.8 \cdot 10^{-6}$
P_{n21}	$3.8 \cdot 10^{-6}$	$3.8 \cdot 10^{-6}$
P_{n12}	0.11	0.11
P_{n22}	0.11	0.11
P_{n13}	0.11	0.11
P_{n23}	0.11	0.11
P_{n14}	$3.8 \cdot 10^{-6}$	$3.8 \cdot 10^{-6}$
P_{n24}	$3.8 \cdot 10^{-6}$	$3.8 \cdot 10^{-6}$
(P_{n15})	$3.8 \cdot 10^{-6}$	$3.8 \cdot 10^{-6}$
(P_{n25})	$3.8 \cdot 10^{-6}$	$3.8 \cdot 10^{-6}$
P_{n16}	not practical	Inf
P_{n26}	not practical	Inf

All in all scenario r) shows good agreement between analytic and algorithm solution. Deviation is seen due to translation from metric to ordinal scale (R_{n1} and R_{n2}) leading to deviations in all P_n values starting with 1 and 2 indicative of R_{n1}/R_{n2} being multiplied with the normalized charge. q_{n6} is not practical as we look at the artificial setting of starting with a total intensity of 0, leading to P_n values with P_{nx6} being at infinity.

s)
Slice 7: $2^{16} - 1$, P(8305/9955/4875)
(+5, +20 voxels from M)
Slice 7: $-(2^{16} - 1)$, P(9405/9955/4875)
(+15,+20 voxels from M)
Middle: P(7755/7755/4875)
The situation can be seen visualized in Fig. S37 s).

Table S31 Scenario s) Two charges in distance of 20/5 and 20/15 voxels to the middle **M**

Parameter	Analytic	Algorithm
q_+ ($\frac{1}{nm^3}$)	$7.2 \cdot 10^{-3}$	$7.2 \cdot 10^{-3}$
$\lvert\mathbf{MR_{neg}}\rvert$ (nm)	2750	2750
$\lvert\mathbf{MR_{pos}}\rvert$ (nm)	2267.7	2267.7
$\lvert\mathbf{R_{pos}R_{neg}}\rvert$ (nm)	1100	1100
n_{pos}	1	1
R_{n1}	0.11	0.11
R_{n2}	0.11	0.11
q_{n1}	$3.5 \cdot 10^{-5}$	$3.5 \cdot 10^{-5}$
q_{n2}	1	1
q_{n3}	1	1
q_{n4}	$3.5 \cdot 10^{-5}$	$3.5 \cdot 10^{-5}$
(q_{n5})	$3.5 \cdot 10^{-5}$	$3.5 \cdot 10^{-5}$
q_{n6}	not practical	Inf
P_{n11}	$3.8 \cdot 10^{-6}$	$3.8 \cdot 10^{-6}$
P_{n21}	$3.8 \cdot 10^{-6}$	$3.8 \cdot 10^{-6}$
P_{n12}	0.11	0.11
P_{n22}	0.11	0.11
P_{n13}	0.11	0.11
P_{n23}	0.11	0.11
P_{n14}	$3.8 \cdot 10^{-6}$	$3.8 \cdot 10^{-6}$
P_{n24}	$3.8 \cdot 10^{-6}$	$3.8 \cdot 10^{-6}$
(P_{n15})	$3.8 \cdot 10^{-6}$	$3.8 \cdot 10^{-6}$
(P_{n25})	$3.8 \cdot 10^{-6}$	$3.8 \cdot 10^{-6}$
P_{n16}	not practical	Inf
P_{n26}	not practical	Inf

All in all scenario s) shows good agreement between analytic and algorithm solution. Deviation is seen due to translation from metric to ordinal scale (R_{n1} and R_{n2}) leading to deviations in all P_n values starting with 1 and 2 indicative of R_{n1}/R_{n2} being multiplied with the normalized charge. q_{n6} is not practical as we look at the artificial setting of starting with a total intensity of 0, leading to P_n values with P_{nx6} being at infinity.

Supplementary References

[1] Yan Gong, Rachel Varnau, Eva-Sophie Wallner, Raghav Acharya, Dominique C. Bergmann, and Lily S. Cheung. Quantitative and dynamic cell polarity tracking in plant cells. *New Phytologist*, 230(2):867–877, 2021. ISSN 0028-646X.

[2] Ani Grigoryan, Novella Guidi, Katharina Senger, Thomas Liehr, Karin Soller, Gina Marka, Angelika Vollmer, Yolanda Markaki, Heinrich Leonhardt, Christian Buske, Daniel B. Lipka, Christoph Plass, Yi Zheng, Medhanie A. Mulaw, Hartmut Geiger, and Maria Carolina Florian. Lamina/c regulates epigenetic and chromatin architecture changes upon aging of hematopoietic stem cells. *Genome Biology*, 19(1):189, 2018. ISSN 1474-760X. doi: 10.1186/s13059-018-1557-3. URL https://doi.org/10.1186/s13059-018-1557-3.

[3] Su Ee Tan, Weijie Tan, Katherine H. Fisher, and David Strutt. Quantify-polarity, a new tool-kit for measuring planar polarized protein distributions and cell properties in developing tissues. *Development*, 148(18):dev198952, 2021. ISSN 0950-1991.
