# Supplementary material for: Quantitative determination of the spatial distribution of components in single cells with CellDetail
Source: Nat Commun. 2024 Nov 26;15:10250. doi: 10.1038/s41467-024-54638-8 (PMC11599593; doi:10.1038/s41467-024-54638-8)
Supplement: Supplementary file 2 — Description of Additional Supplementary Files [file 41467_2024_54638_MOESM2_ESM.pdf]

Description of Additional Supplementary Files:

Supplementary Movie 1: HSC polar for Cdc42 and Tubulin, confocal image data (red: Cdc42, green: Tubulin, blue: DAPI)

Supplementary Movie 2: HSC apolar for Cdc42 and Tubulin, confocal image data (red: Cdc42, green: Tubulin, blue: DAPI)
