# Supplementary material for: Quantitative determination of the spatial distribution of components in single cells with CellDetail
Source: Nat Commun. 2024 Nov 26;15:10250. doi: 10.1038/s41467-024-54638-8 (PMC11599593; doi:10.1038/s41467-024-54638-8)
Supplement: Supplementary file 5 — Reporting Summary [file 41467_2024_54638_MOESM5_ESM.pdf]

nature portfolio

Corresponding author(s): Hartmut Geiger
Tanja Schuster

Last updated by author(s): Tanja Schuster, 18.10.2024

Reporting Summary

Nature Portfolio wishes to improve the reproducibility of the work that we publish. This form provides structure for consistency and transparency in reporting. For further information on Nature Portfolio policies, see our Editorial Policies and the Editorial Policy Checklist.

Statistics

For all statistical analyses, confirm that the following items are present in the figure legend, table legend, main text, or Methods section.

n/a	Confirmed	
☐	☒	The exact sample size (n) for each experimental group/condition, given as a discrete number and unit of measurement
☐	☒	A statement on whether measurements were taken from distinct samples or whether the same sample was measured repeatedly
☐	☒	The statistical test(s) used AND whether they are one- or two-sided<br>*Only common tests should be described solely by name; describe more complex techniques in the Methods section.*
☒	☐	A description of all covariates tested
☒	☐	A description of any assumptions or corrections, such as tests of normality and adjustment for multiple comparisons
☐	☒	A full description of the statistical parameters including central tendency (e.g. means) or other basic estimates (e.g. regression coefficient) AND variation (e.g. standard deviation) or associated estimates of uncertainty (e.g. confidence intervals)
☐	☒	For null hypothesis testing, the test statistic (e.g. F, t, r) with confidence intervals, effect sizes, degrees of freedom and P value noted<br>*Give P values as exact values whenever suitable.*
☒	☐	For Bayesian analysis, information on the choice of priors and Markov chain Monte Carlo settings
☒	☐	For hierarchical and complex designs, identification of the appropriate level for tests and full reporting of outcomes
☐	☒	Estimates of effect sizes (e.g. Cohen's d, Pearson's r), indicating how they were calculated

Our web collection on statistics for biologists contains articles on many of the points above.

Software and code

Policy information about availability of computer code

Data collection	Zeiss ZEN Software was used for measuring immunofluorescent images.
Data analysis	custom code CellDetail software, version 37. https://github.com/xyq91/CellDetail-TS, DOI: 10.5281/zenodo.13860319

For manuscripts utilizing custom algorithms or software that are central to the research but not yet described in published literature, software must be made available to editors and reviewers. We strongly encourage code deposition in a community repository (e.g. GitHub). See the Nature Portfolio guidelines for submitting code & software for further information.

Data

Policy information about availability of data

All manuscripts must include a data availability statement. This statement should provide the following information, where applicable:

- Accession codes, unique identifiers, or web links for publicly available datasets
- A description of any restrictions on data availability
- For clinical datasets or third party data, please ensure that the statement adheres to our policy

The authors declare that the data supporting the findings of this study are available within the paper and its Supplementary Information files. Source data are provided with this paper. The data generated in this study are provided in the Source Data file.

Research involving human participants, their data, or biological material

Policy information about studies with human participants or human data. See also policy information about sex, gender (identity/presentation), and sexual orientation and race, ethnicity and racism.

Reporting on sex and gender	Research involving human research participants has not been performed.
Reporting on race, ethnicity, or other socially relevant groupings	*Please specify the socially constructed or socially relevant categorization variable(s) used in your manuscript and explain why they were used. Please note that such variables should not be used as proxies for other socially constructed/relevant variables (for example, race or ethnicity should not be used as a proxy for socioeconomic status).<br>Provide clear definitions of the relevant terms used, how they were provided (by the participants/respondents, the researchers, or third parties), and the method(s) used to classify people into the different categories (e.g. self-report, census or administrative data, social media data, etc.)<br>Please provide details about how you controlled for confounding variables in your analyses.*
Population characteristics	*Describe the covariate-relevant population characteristics of the human research participants (e.g. age, genotypic information, past and current diagnosis and treatment categories). If you filled out the behavioural & social sciences study design questions and have nothing to add here, write "See above."*
Recruitment	*Describe how participants were recruited. Outline any potential self-selection bias or other biases that may be present and how these are likely to impact results.*
Ethics oversight	*Identify the organization(s) that approved the study protocol.*

Note that full information on the approval of the study protocol must also be provided in the manuscript.

Field-specific reporting

Please select the one below that is the best fit for your research. If you are not sure, read the appropriate sections before making your selection.

☒ Life sciences ☐ Behavioural & social sciences ☐ Ecological, evolutionary & environmental sciences

For a reference copy of the document with all sections, see nature.com/documents/nr-reporting-summary-flat.pdf

Life sciences study design

All studies must disclose on these points even when the disclosure is negative.

Sample size	HSCs: Sample sizes were chosen as the minimal number of measured cells obtained per mouse, for pooling to total cell number in equivalent weighting. The reached sample size is sufficient for obtaining a distribution overview. Cdc42/Tubulin confocal: 217 young HSCs, 372 old HSCs, widefield: 630 young HSCs, 1370 old HSCs, Septins 330 young HSCs, 440 old HSCs. FF95: Sample sizes were chosen as the number of cells measured for analysis. The reached sample size is sufficient for obtaining a distribution overview. (50 control FF95, 68 senescent FF95)
Data exclusions	HSCs: no image data were excluded from analysis<br>FF95: if cells were overlapping with protrusions, they were not taken for analysis with CellDetail due to single cell requirement of CellDetail.
Replication	The focus of this manuscript is on the software CellDetail and its algorithm.<br>HSCs: The variability of mice is included in equivalent weighting by taking the same cell number from each repetition, which is combined in the final data. FF95: data were included with the main focus to show CellDetail's versatility on other cell types. A replication was not performed.
Randomization	Randomization was not relevant to this study due to objectiveness of CellDetail. Image data is analyzed without subjectiveness of the user.
Blinding	Blinding was not relevant to our study due to objectiveness of CellDetail. Image data is analyzed without subjectiveness of the user.

Reporting for specific materials, systems and methods

We require information from authors about some types of materials, experimental systems and methods used in many studies. Here, indicate whether each material, system or method listed is relevant to your study. If you are not sure if a list item applies to your research, read the appropriate section before selecting a response.

Materials & experimental systems

n/a	Involved in the study	
☐	☒	Antibodies
☐	☒	Eukaryotic cell lines
☒	☐	Palaeontology and archaeology
☐	☒	Animals and other organisms
☒	☐	Clinical data
☒	☐	Dual use research of concern
☒	☐	Plants

Methods

n/a	Involved in the study	
☒	☐	ChIP-seq
☐	☒	Flow cytometry
☒	☐	MRI-based neuroimaging

Antibodies

Antibodies used

anti-B220 Biotin (eBioscience, Clone 53-7.3)
anti-CD5 Biotin (eBioscience, Clone 53-7.3)
anti-CD8a Biotin (eBioscience, Clone53-6.7)
anti-CD11b Biotin (eBioscience, Clone M1/70)
anti-Gr-1 Biotin (eBioscience, Clone RB6-8C5)
anti-Terr-119 Biotin (eBioscience, Clone TER-119)
anti-CD34-FITC (eBioscience, Clone RAM34)
anti-ckit-APC (eBioscience, Clone ACK2)
anti-Flt3-PE (eBioscience, Clone A2F10)
anti-SA-e450 (eBioscience, Streptavidin eFluor 450)
anti-Sca-1-PeCy7 (eBioscience, anti-Mo-Ly-6A/E, Clone D7)
anti-Cdc42 (Abcam, ab64533)
anti-Septin1-AF790 (Santa Cruz Biotechnology, sc-373925AF790)
anti-Septin2 (Abcam, ab58657)
anti-Septin6-AF546 (Santa Cruz Biotechnology, sc-514781 AF546)
anti-Septin7 (Proteintech, 13818-1-AP)
anti-Septin9 (Proteintech, 10769-1-AP)
anti-Septin11 (Proteintech, 14672-1-AP)
anti-Tubulin (Abcam, ab6160)
anti-rat AF488 (Jackson ImmunoResearch, 712-545-153)
anti-rb AF594 (Jackson ImmunoResearch, 711-585-152)
anti-Cdc42 (Abcam, ab41429)
anti-mouse AF488 (Jackson ImmunoResearch, 715-545-150)

Validation

anti-B220 Biotin (eBioscience, Clone 53-7.3): manufacterer relative expression validated, 192 references, flow cytometry
anti-CD5 Biotin (eBioscience, Clone 53-7.3): 24 references, flow cytometry
anti-CD8a Biotin (eBioscience, Clone53-6.7): 99 references, flow cytometry
anti-CD11b Biotin (eBioscience, Clone M1/70): 647 references, flow cytometry
anti-Gr-1 Biotin (eBioscience, Clone RB6-8C5): 360 references, flow cytometry
anti-Terr-119 Biotin (eBioscience, Clone TER-119): 144 references, flow cytometry
anti-CD34-FITC (eBioscience, Clone RAM34): 155 references, flow cytometry
anti-ckit-APC (eBioscience, Clone ACK2): 31 references, flow cytometry
anti-Flt3-PE (eBioscience, Clone A2F10): 71 references, flow cytometry
anti-Sca-1-PeCy7 (eBioscience, anti-Mo-Ly-6A/E, Clone D7): 105 references, flow cytometry
anti-Cdc42 (Abcam, ab64533): manufacterer KO-validated, 40 references, IF
anti-Septin1-AF790 (Santa Cruz Biotechnology, sc-373925AF790): 1 references, IF
anti-Septin2 (Abcam, ab58657): 2 references, IF
anti-Septin6-AF546 (Santa Cruz Biotechnology, sc-514781 AF546): 1 reference, IF
anti-Septin7 (Proteintech, 13818-1-AP): 21 references, IF
anti-Septin9 (Proteintech, 10769-1-AP): 26 references, IF
anti-Septin11 (Proteintech, 14672-1-AP): 3 references, IF
anti-Tubulin (Abcam, ab6160): 500 references, IF
anti-rat AF488 (Jackson ImmunoResearch, 712-545-153): 162 references
anti-rb AF594 (Jackson ImmunoResearch, 711-585-152): 188 references
anti-Cdc42 (Abcam, ab41429): 16 references
anti-mouse AF488 (Jackson ImmunoResearch, 715-545-150): 761 references

Eukaryotic cell lines

Policy information about cell lines and Sex and Gender in Research

Cell line source(s)

Primary human dermal fibroblasts (HDF) FF95 were previously established from foreskin of a one year old healthy male having undergone circumcision as described in Raul Fleischmajer et al., Variability in collagen and fibronectin synthesis by scleroderma fibroblasts in primary culture (1981) and Lale Naderi-Hachtroudi et al., Induction of manganese superoxide dismutase in human dermal fibroblasts: a uv-b-mediated paracrine mechanism with the release of epidermal interleukin 1 alpha, interleukin 1beta, and tumor necrosis factor alpha (2002).

Authentication	Céline Borlon et al., The gene expression profile of psoralen plus UVA-induced premature senescence in skin fibroblasts resembles a combined DNA-damage and stress-induced cellular senescence response phenotype, Elsevier, 2007
Mycoplasma contamination	Céline Borlon et al., The gene expression profile of psoralen plus UVA-induced premature senescence in skin fibroblasts resembles a combined DNA-damage and stress-induced cellular senescence respone phenotype, Elsevier, 2007
Commonly misidentified lines (See ICLAC register)	Céline Borlon et al., The gene expression profile of psoralen plus UVA-induced premature senescence in skin fibroblasts resembles a combined DNA-damage and stress-induced cellular senescence response phenotype, Elsevier, 2007

Animals and other research organisms

Policy information about studies involving animals; ARRIVE guidelines recommended for reporting animal research, and Sex and Gender in Research

Laboratory animals	mice
Wild animals	*Provide details on animals observed in or captured in the field; report species and age where possible. Describe how animals were caught and transported and what happened to captive animals after the study (if killed, explain why and describe method; if released, say where and when) OR state that the study did not involve wild animals.*
Reporting on sex	female
Field-collected samples	*For laboratory work with field-collected samples, describe all relevant parameters such as housing, maintenance, temperature, photoperiod and end-of-experiment protocol OR state that the study did not involve samples collected from the field.*
Ethics oversight	All experiments were performed in compliance with German Law for Welfare of Laboratory Animals and were approved by the Regierungspräsidium Tübingen.

Note that full information on the approval of the study protocol must also be provided in the manuscript.

Plants

Seed stocks	Research involving plants has not been performed.
Novel plant genotypes	*Describe the methods by which all novel plant genotypes were produced. This includes those generated by transgenic approaches, gene editing, chemical/radiation-based mutagenesis and hybridization. For transgenic lines, describe the transformation method, the number of independent lines analyzed and the generation upon which experiments were performed. For gene-edited lines, describe the editor used, the endogenous sequence targeted for editing, the targeting guide RNA sequence (if applicable) and how the editor was applied.*
Authentication	*Describe any authentication procedures for each seed stock used or novel genotype generated. Describe any experiments used to assess the effect of a mutation and, where applicable, how potential secondary effects (e.g. second site T-DNA insertions, mosiacism, off-target gene editing) were examined.*

Flow Cytometry

Plots

Confirm that:

☒ The axis labels state the marker and fluorochrome used (e.g. CD4-FITC).

☒ The axis scales are clearly visible. Include numbers along axes only for bottom left plot of group (a 'group' is an analysis of identical markers).

☒ All plots are contour plots with outliers or pseudocolor plots.

☒ A numerical value for number of cells or percentage (with statistics) is provided.

Methodology

Sample preparation	BM of mice, low-density gradient centrifugation, lineage depletion, stem cell markers
Instrument	BD FACS Aria II (BD Biosciences)
Software	FACSDiva Version 6.1.3
Cell population abundance	due to low cell number of LT-HSCs, no purity check of the samples was done

Gating strategy

LT-HSCs were gated by FSC and SSC based on size and granularity of single cells of interest, then further as lineage negative (e450 negative) Sca-1 positive c-kit-positive (LSK), CD34 negative and Flt3 negative cells, as described in Florian MC et al., A canonical to non-canonical Wnt signalling switch in haematopietic stem-cell ageing. Nature. 2013

☒ Tick this box to confirm that a figure exemplifying the gating strategy is provided in the Supplementary Information.
